# Hierarchical resource rationality explains human reading behaviour

**DOI:** 10.1038/s41562-026-02534-0

**Published:** 2026-08-10

**Authors:** Yunpeng Bai, Xiaofu Jin, Shengdong Zhao, Antti Oulasvirta

**Affiliations:** 1 Department of Computer Science, School of Computing, National University of Singapore, Singapore, Singapore; 2 Department of Information and Communications Engineering, School of Electrical Engineering, Aalto University, Espoo, Finland; 3 Computational Media and Arts Thrust, Information Hub, The Hong Kong University of Science and Technology, Guangzhou, China; 4 Academy of Interdisciplinary Studies, The Hong Kong University of Science and Technology, Hong Kong, China; 5 School of Creative Media, Department of Computer Science, City University of Hong Kong, Hong Kong, China; 6 ELLIS Institute Finland, Espoo, Finland

**Keywords:** Human behaviour, Decision making

## Abstract

Reading is a pervasive and cognitively demanding activity that underpins modern human culture. It is a prime instance of a class of tasks where eye movements are coordinated for the purpose of comprehension. Existing theories explain either eye movements or comprehension during reading, but the critical link between the two remains unclear. Here we propose resource-rational optimization as a unifying control principle governing adaptive reading behaviour. Eye movements are selected to maximize expected comprehension while minimizing cognitive and temporal costs, organized hierarchically across nested time scales: fixation decisions support word recognition; sentence-level integration guides skipping and regression; and text-level comprehension goals shape memory construction and rereading. A computational implementation successfully replicates an unprecedented range of findings in human reading, from lexical effects to comprehension outcomes. Together, these results suggest that resource rationality provides a general mechanism for coordinating perception, memory and action in knowledge-intensive human behaviours, offering a principled account of how complex cognitive skills adapt to limited resources.

## Abstract (web summary)

This work shows that human reading behaviour can be explained as hierarchical resource-rational control, unifying eye movements, memory and comprehension in one computational principle.

## Main

Humans routinely engage in complex cognitive activities that require coordinating perception, memory and action over time, under limited resources of attention, memory and available time^1–3^. Explaining how such behaviour remains adaptive when decisions are sequential and uncertain remains a central challenge for cognitive science^4,5^. Although theories of bounded cognition characterize optimal behaviour under constraints, how these principles operate in naturalistic, knowledge-intensive tasks is still poorly understood^1,2^.

Reading exemplifies this class of resource-constrained, sequential behaviours: a ubiquitous and cognitively demanding activity in which eye movements and memory must be coordinated over time to support comprehension^6^. Beyond its practical importance, reading provides a naturalistic setting in which to study how humans allocate attention and memory over time to support comprehension under resource constraints^6,7^. Computational approaches to explain reading have proceeded along two complementary paths. The first describes how cognition guides eye movements^8–13^, and the second focuses on how memory and comprehension are constructed^7,14,15^. Within the first path, word recognition is assumed to proceed serially with shifts of attention^8^, with lexical processing stages directly triggering eye movements, as being distributed across several words at once, with eye movements resulting from a stochastic competition between their activations^9^ or as an uncertainty-reduction problem: eye movements are chosen to minimize entropy over word identity^10,11^. These approaches focus on capturing local lexical control, leaving unclear how eye movements are guided by evolving comprehension and memory demands over time. Approaches using a machine learning technique called conditional generation^12,13^ treat fixations as a mapping from sentence word orders and semantic meanings. Although this approach shows high predictive accuracy, it lacks explicit cognitive assumptions about memory and control. The second path comprises theories of comprehension in reading^7,14–16^, which centre on the construction–integration process. It is assumed that a schema guides working memory in building a coherent propositional text base and reducing it into a gist. Although these theories capture the formation of meaning, they do not explain how this, in turn, influences eye-movement control. Über-Reader^17^ represents an important step towards integrating eye-movement control and comprehension processes within a unified framework, combining mechanisms for word identification, sentence processing and discourse representation. In this architecture, eye-movement behaviour is generated by interactions among processing stages governed by parameterized processing mechanisms. Although this approach provides a detailed and empirically grounded account of multilevel reading processes, control is implemented through predefined interactions among processing components. As a result, a general account of how high-level reading objectives (for example, comprehension) and low-level eye-movement control are jointly governed by a single control principle remains lacking.

In this work, we provide strong evidence for a unified computational basis underpinning reading as a joint optimization of eye movement and comprehension. We propose that resource-rational control^1,18,19^ provides a general theoretical account of how humans coordinate perception, memory and action to maximize long-term utility under limited resources during reading, and we instantiate this claim in a computational model. The key idea is that reading is adaptive decision-making: readers choose where to look next on the basis of uncertain and partial information, to efficiently build a coherent understanding of the text. Fundamentally, reading is a process of sequential information sampling: each fixation contributes evidence that updates memory and supports comprehension, whereas future eye movements are chosen in light of these evolving internal states. Moreover, evidence from eye-tracking and neuroscience further shows that cognition is organized hierarchically across multiple time scales^20–22^. Accordingly, the principle of resource rationality can be extended to derive hierarchical action representations that balance behavioural efficiency with flexibility^1,23^. These principles naturally link high-level cognition with low-level eye movements: fixations emerge as consequences of boundedly optimal actions selected to maximize comprehension goals. Sequential sampling under hierarchical control ensures that low-level eye movements strategically accumulate information for memory step by step, providing the foundation on which higher-level comprehension processes are built.

**Fig. 1:**
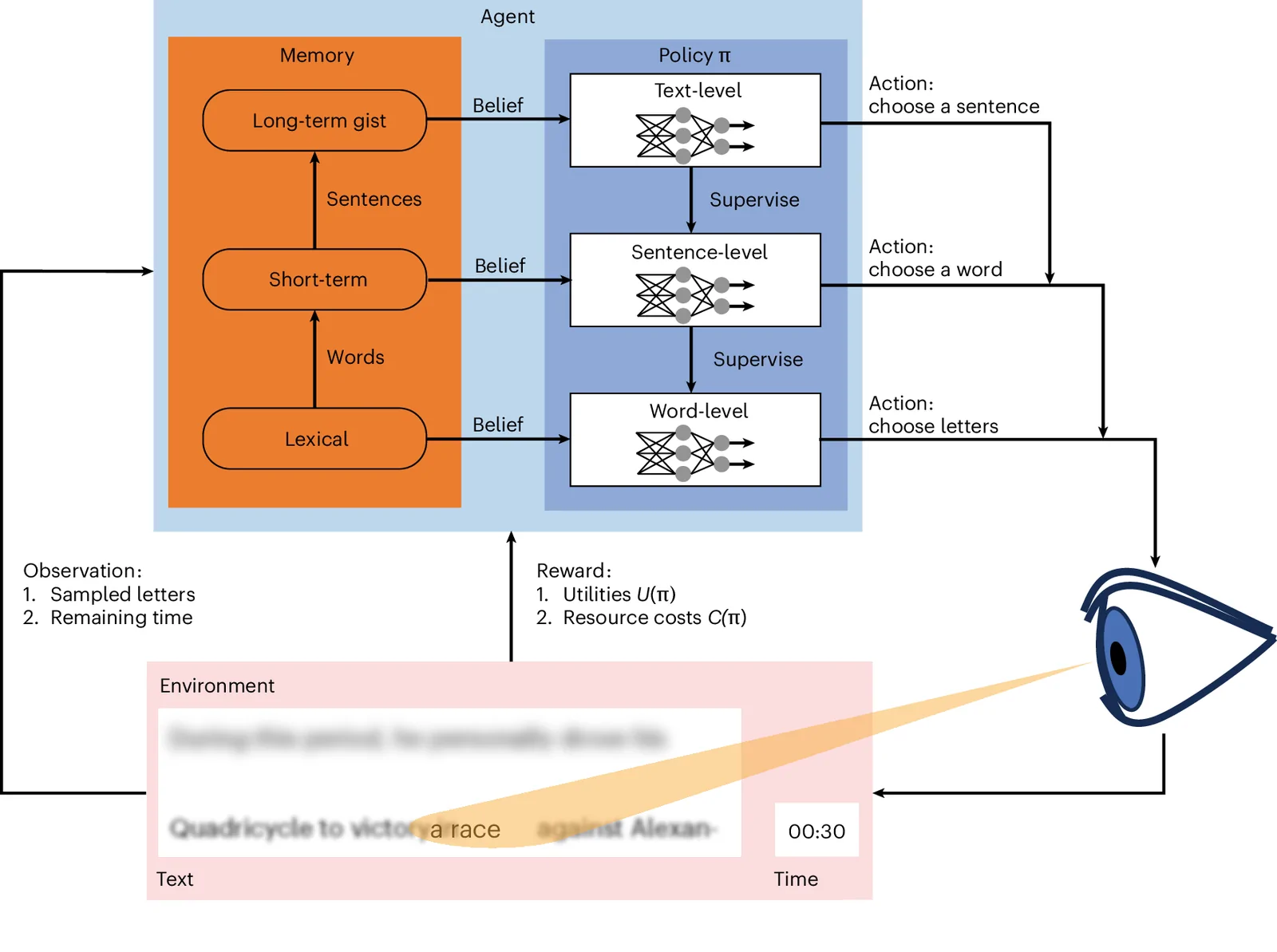
A resource-rational mechanism for reading. The reader is simulated as a resource-rational^1,18^ agent that maximizes text comprehension (goal) by optimizing fixations on a text display (external environment), under constraints of limited resources such as memory, visual perception and time. The agent is uncertain about the true states because its observations are partial. To handle such uncertainties, it maintains probabilistic beliefs over the word, sentence and text states through three memory stores—lexical, short-term and long-term. Decisions are made hierarchically: high-level text comprehension guide sentence-level readings, which then guides word recognition and eye-movement control. Overall, the model treats reading as a process of selecting where to look next on the basis of the current state of understanding and the expected informativeness of future observations.

**Fig. 2:**
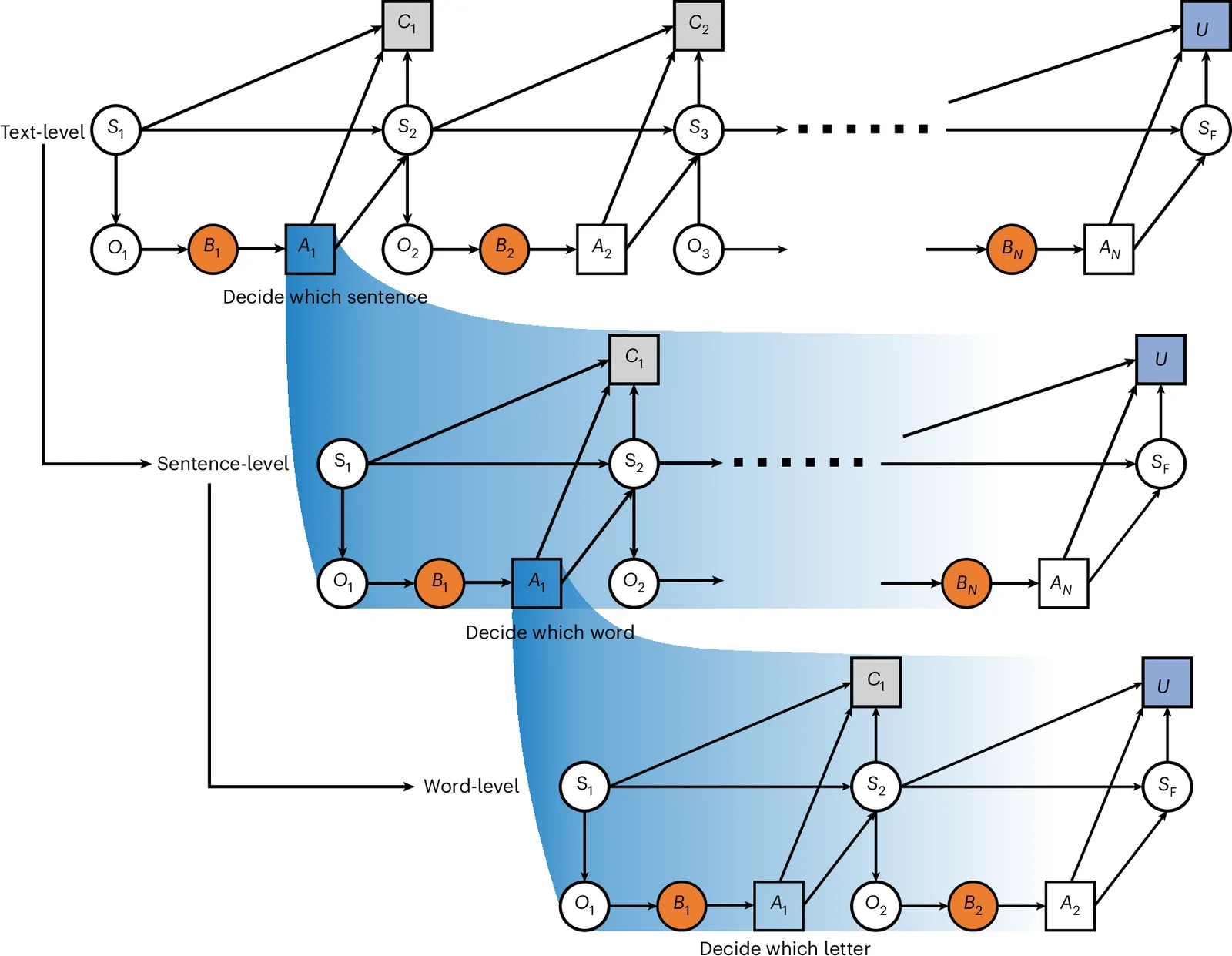
Hierarchical resource-rational control of reading formalized as POMDPs. Eye-movement control is organized into three nested levels: text, sentence and word, each operating on its own temporal scale. Higher-level controllers set coarse reading targets, and lower-level controllers execute them at finer scales. For instance, the text-level controller chooses the sentence, the sentence-level chooses the word within that sentence and the word-level controller recognizes that word by deciding how to sample the letters. At each level, the process is formalized as a POMDP. The state *S*_*N*_ describes the reading dynamics; observations *O*_*N*_ reflect noisy visual input sampled from the environment (text stimulus) owing to limited visual attention, forming partial observability from *S*_*N*_. As the true state *S*_*N*_ is not directly observable to the memory, the agent maintains a belief state *B*_*N*_ on the basis of *O*_*N*_ in the memory to handle uncertainties: encoding, for example, coherence appraisals across sentences, word predictions or word activation distributions. Actions *A*_*N*_ are selected on the basis of *B*_*N*_ to advance reading, which in turn updates the state *S*_*N*_ and produces new observations *O*_*N*_ for the next step. *S*_F_ represents the final step at given level. Decision-makings are resource-rational: at each level, the agent learns to select the action that maximizes expected reward, defined as comprehension utility *U* and eye-movement costs *C*_*N*_.

We develop a computational model grounded in these principles to simulate a resource-rational reader. The reader is formalized as an agent that optimizes comprehension through a sequence of decisions, trading off expected cumulative gains in understanding against the costs of eye-movement effort and time under limited visual, memory and temporal resources (Fig. 1). It features a hierarchical control architecture with three interacting levels: word-, sentence- and text-levels, each represented as a controller that contributes to adaptive reading behaviour (Fig. 2).

**Fig. 3:**
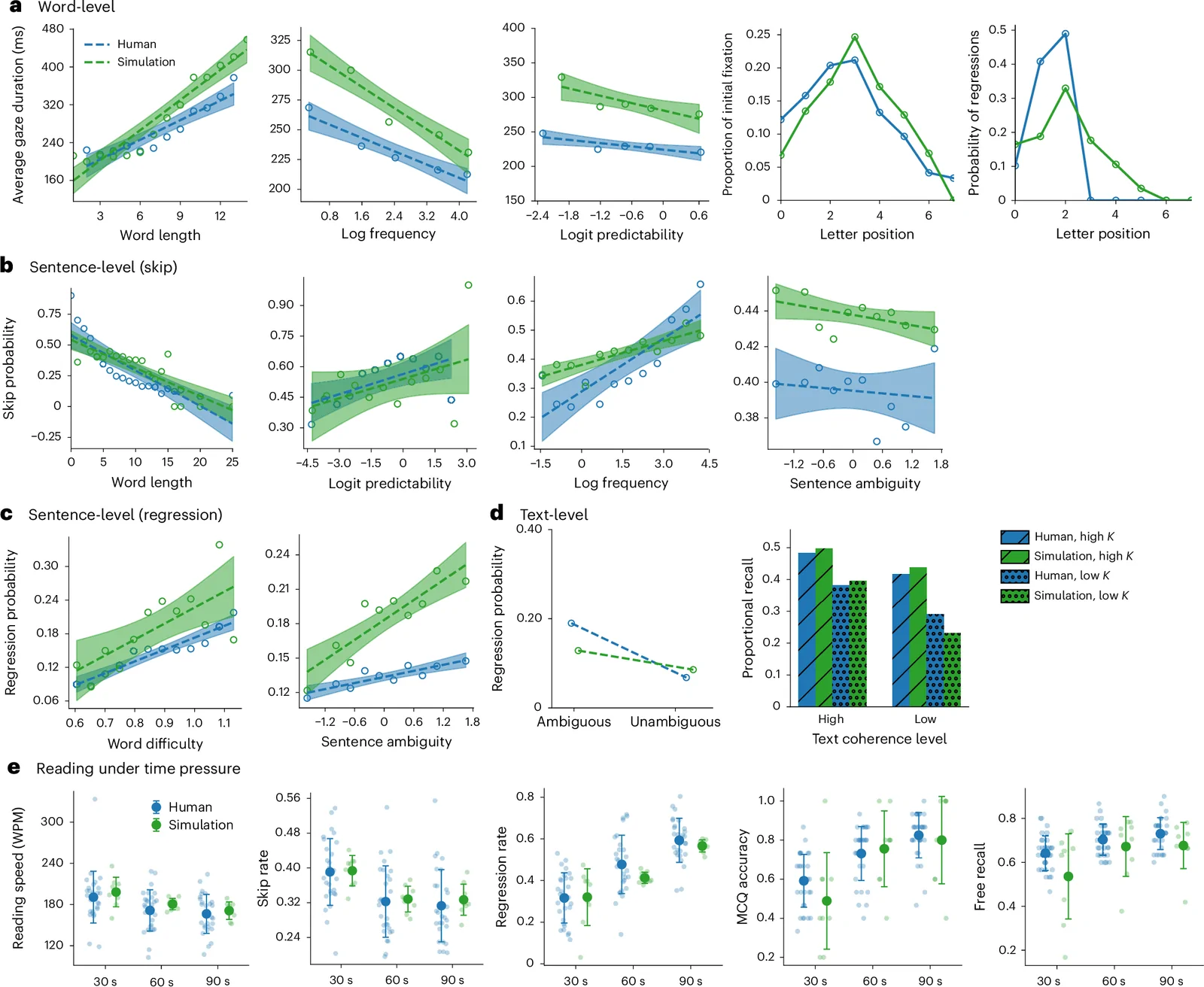
Model reproduces key empirical effects in eye movements and comprehension. **a**,The word-level. Both humans and the simulation show lexical influences on gaze duration: shorter, more frequent and more predictable words receive shorter gaze duration. In addition, the model reproduces spatial aspects of word-level eye movements: initial fixation locations follow the preferred viewing position and intraword regressions are biased towards earlier letter positions, consistent with human data. **b**, The sentence-level (skip). Short, frequent and predictable words are skipped more often. The skipping probability decreases as sentence ambiguity increases. **c**, The sentence-level (regression). Lexically or contextually difficult words elicit more regressions. The regression probability increases with sentence ambiguity. **d**, The text-level. The simulation reproduces comprehension-driven control, with more regressions for ambiguous sentences and improved recall with higher prior knowledge and text coherence. **e**, Reading under time pressure. Adaptive strategy shifts: increasing time pressure leads to faster reading with more skips and fewer regressions, accompanied by reduced multiple-choice accuracy and free recall. Human data are shown in blue and model predictions in green. In **e**, dots show individual observations, large circles show means and error bars indicate s.d. Human eye-movement metrics were computed from participant-level observations (*n* = 28); human comprehension metrics were computed from participant-level means across trials (*n* = 32); simulation metrics were computed from episode–stimulus observations (*n* = 9 per condition). These results demonstrate reading as a resource-rational process: readers adapt their eye movements to linguistic features and available time, producing corresponding adaptations in comprehension. These adaptations reflect a systematic trade-off between accuracy and effort to maximize overall utility across word-, sentence- and text-level processing.

Our model provides a hierarchical resource-rational account that unifies eye-movement control and comprehension under a single optimal-control principle. It accurately reproduces a broad range of known empirical effects of eye movements and comprehension in reading from the principle of resource rationality^1,18^. At the word level (Fig. 3a), the model captures established findings^6,24–28^: longer words prolong fixations, whereas higher frequency and predictability shorten them. It also reproduces the preferred viewing location effect and within-word regression patterns as a function of landing position. These results reflect that eye movements serve as rational control strategies for reducing uncertainty about lexical identity in memory. At the sentence level (Fig. 3b,c), it predicts skips and regressions as rational trade-offs between efficiency and integration accuracy in the short-term memory. At the text level (Fig. 3d), it explains how prior knowledge^29,30^ and coherence^29–31^ improve comprehension and trigger targeted regressions to repair failures in text-level comprehension^32,33^. We collected a new dataset of English reading under time pressure and compared our model’s simulations against it. The model successfully captured human adaptations in skipping, regressions and comprehension decline^34,35^ (Fig. 3e), revealing that readers strategically adjust eye-movement patterns to maximize comprehension under limited time and memory capacities. Crucially, these behaviours are not prescribed by fixed heuristics or task-specific rules but instead emerge from optimizing comprehension under hierarchical resource constraints, highlighting the explanatory necessity of a resource-rational account of reading.

## Computational simulation of reading

We formalize resource-rational control hierarchically to capture how human readers naturally decompose the complex task of understanding text into nested subtasks during reading. The hierarchy consists of three levels operating from low to high (Fig. 2). (1) Word level: the controller decides which letters to fixate for word recognition, formalized as reducing uncertainty in word identity within the lexical memory. (2) Sentence level: the controller selects which word to read next to support sentence comprehension, balancing reading speed against information gain and comprehension accuracy. This is reflected in the trade-off between skips and regressions, primarily involving short-term memory processes. (3) Text level: the controller determines which sentence to read or reread to achieve coherent understanding across the text, coordinating long-term memory construction and coherence monitoring. The three controllers operate at different temporal and informational scales but interact continuously. For example, the sentence-level controller instructs the lexical controller which word to process next. The lexical controller then directs visual attention to that location, deploying fixations to sample letters and identify the word. The recognized word is passed back as perceptual evidence to the sentence-level controller, which updates its representation of sentence meaning and, in turn, contributes to text-level comprehension governed by the higher controller.

Each controller’s control problem is represented as a partially observable Markov decision process (POMDP)^36^ (Fig. 2), a mathematical framework for modelling sequential decision-making under uncertainty. We adopt this formulation to describe reading’s resource-rational control because reading is inherently sequential: at each step, the agent decides where to look next on the basis of its current internal state, including what has been seen, remembered and understood so far and its progress towards comprehension. The POMDP also captures the uncertainty and partial observability intrinsic to reading. Readers cannot fully observe the text at once; letters and words outside fixation are only partially visible; thus, decisions must be made under uncertainty using imperfect sensory and memory information. Within this formalism, the belief state compactly summarizes ongoing comprehension and guides the next eye-movement action. Each controller is trained independently at its own level using deep reinforcement learning^37^, which learns boundedly optimal policies that maximize comprehension utility while minimizing the costs of eye-movement effort and time. Reinforcement learning is used here as a computational method for approximating optimal decision policies in partially observable, sequential environments where analytic solutions are intractable^36^. In reading, the agent must act under uncertainty about word identity, sentence meaning and text-level coherence, while balancing immediate costs against long-term comprehension gains. These properties make it difficult to specify behaviour using predefined rules, whereas reinforcement learning enables policies to emerge from optimizing these trade-offs directly. After training, the three controllers are integrated to function as a unified model capable of simulating natural reading behaviours.

Formally, each level is modelled as a POMDP defined by states (representing the current reading context), observations (noisy visual input and memory signals), actions (for example, fixation, skip and regress) and rewards (balancing comprehension gains against time and effort costs). As the true state is not directly observable, the agent maintains a belief state, that is, a probabilistic summary of its current understanding which guides action selection.

Intuitively, a POMDP formalizes reading as a sequence of decisions made under uncertainty. At any moment, the reader does not have direct access to the true state of the text (for example, exact word identity or sentence meaning) but instead must act on the basis of partial visual input and memory. These inputs are integrated into an internal belief, a probabilistic representation of the reader’s current understanding, which is updated as new evidence is acquired. Each fixation provides noisy information that refines this belief. On the basis of the current belief, the reader selects actions such as fixating, skipping or regressing to improve comprehension while minimizing time and effort. In this sense, reading can be understood as a process of actively sampling information to reduce uncertainty about meaning under resource constraints. For example, when encountering a highly predictable context (for example, ‘drink a cup of ___’), the reader’s belief over the upcoming word is already concentrated. In this case, the expected gain from fixating the word is low, and the optimal policy may skip it to save time. Conversely, when a word is unexpected or difficult to integrate, the belief remains uncertain, increasing the expected value of additional sampling. The model therefore predicts regressions or longer fixations in such cases. These behaviours arise from an optimized policy that evaluates the expected value of actions under uncertainty, rather than from fixed rules.

At each level of the hierarchy, actions are selected to maximize expected utility while minimizing associated costs (for example, time and eye-movement effort). Importantly, the model does not impose explicit stopping rules or predefined thresholds for terminating processing. Instead, termination emerges as part of the policy: the agent decides whether to continue sampling information or to stop on the basis of its current belief state and the expected value of further actions. Control is transferred across levels when the policy selects termination actions rather than being triggered by externally specified criteria. Coordination between levels does not rely on explicit communication about information requirements. Instead, it is mediated implicitly through belief states and value: higher-level policies shape lower-level behaviour by defining goals and reward functions, whereas lower-level processing updates beliefs that determine the expected utility of continuing or terminating. In this sense, both the type and amount of information required are encoded in the agent’s value function, rather than communicated through separate control signals. For example, during sentence reading, the agent processes the current word while forming predictions about upcoming words on the basis of contextual information (stored in the short-term memory) and parafoveal preview. These beliefs guide action selection, including whether to fixate, skip or regress. When uncertainty about comprehension remains high, the agent may regress to earlier words to acquire additional information; when the expected value of further processing is low, it proceeds to the next word or sentence. At the text level, the same principle governs decisions about rereading versus advancing. Thus, coordination between levels arises from policy-driven control under a shared resource-rational principle, rather than from explicit communication protocols or fixed stopping rules. The importance of these design choices is supported by ablation results (Fig. 5), where removing hierarchical structure or altering long-term value sensitivity leads to degraded and less human-like performance, indicating that effective interaction between levels is essential for reproducing human reading behaviour.

## Results

We compare our model’s simulation results against a set of established empirical effects at the word^6,24,27,28^, sentence^6,24,32,38^ and text levels^7,29,33^; as well as reading under time constraints^35^. These well-established effects have long been used to characterize the adaptive dynamics of human reading, reflecting how eye movements and linguistic processing interact under resource constraints. Successfully reproducing them provides a rigorous test of whether the model captures the hierarchical resource-rational mechanisms in reading.

### Deciding when and where to fixate in a word

Word-level eye-movement behaviour reflects how readers manage uncertainty during lexical identification under limited visual, attentional and memory resources. Empirical findings show that gaze duration, defined as the sum of all fixation durations on a word during its first pass, varies systematically with word length, frequency and contextual predictability^6,24^. Our simulations reproduced the direction of the human lexical effects on gaze duration (Fig. 3a and Table 1). Across the aggregate values plotted in the figure, gaze duration increased with word length and decreased with log frequency in both human and simulated data. Gaze duration also showed a negative aggregate trend with logit predictability in both human and simulated data, although neither trend reached significance in the aggregate regressions over the five plotted bins.

**Table 1:** Summary of inferential statistical analyses

Analysis	Outcome	Predictor/contrast	Dataset	Estimate	Test statistic	*P* value, effect size (95% CI)
Word recognition	Gaze duration	Word length	Human	*β* = 13.85	*t*(10) = 7.45	*P* < 0.001, *R*^2^ = 0.85 (9.71 to 18.00)
Word recognition	Gaze duration	Word length	Simulation	*β* = 21.31	*t*(12) = 11.61	*P* < 0.001, *R*^2^ = 0.92 (17.31 to 25.31)
Word recognition	Gaze duration	Log frequency	Human	*β* = −14.01	*t*(3) = − 5.89	*P* = 0.010, *R*^2^ = 0.92 (−21.58 to -6.43)
Word recognition	Gaze duration	Log frequency	Simulation	*β* = −22.27	*t*(3) = −7.09	*P* = 0.006, *R*^2^ = 0.94 (−32.26 to −12.27)
Word recognition	Gaze duration	Logit predictability	Human	*β* = −7.83	*t*(3) = −2.72	*P* = 0.072, *R*^2^ = 0.71 (−16.99 to 1.33)
Word recognition	Gaze duration	Logit predictability	Simulation	*β* = −17.68	*t*(3) = −2.71	*P* = 0.073, *R*^2^ = 0.71 (−38.45 to 3.08)
Sentence reading	Skip probability	Word length	Human	*β* = −0.029	*t*(18) = −6.44	*P* < 0.001, *R*^2^ = 0.70 (−0.038 to −0.019)
Sentence reading	Skip probability	Word length	Simulation	*β* = −0.023	*t*(18) = −6.92	*P* < 0.001, *R*^2^ = 0.73 (−0.030 to −0.016)
Sentence reading	Skip probability	Logit predictability	Human	*β* = 0.033	*t*(10) = 2.62	*P* = 0.025, *R*^2^ = 0.41 (0.005 to 0.061)
Sentence reading	Skip probability	Logit predictability	Simulation	*β* = 0.032	*t*(10) = 1.59	*P* = 0.144, *R*^2^ = 0.20 (−0.013 to 0.076)
Sentence reading	Skip probability	Log frequency	Human	*β* = 0.061	*t*(10) = 4.87	*P* < 0.001, *R*^2^ = 0.70 (0.033 to 0.089)
Sentence reading	Skip probability	Log frequency	Simulation	*β* = 0.027	*t*(10) = 5.33	*P* < 0.001, *R*^2^ = 0.74 (0.016 to 0.039)
Sentence reading	Regression probability	Word difficulty	Human	*β* = 0.212	*t*(10) = 9.30	*P* < 0.001, *R*^2^ = 0.90 (0.161 to 0.262)
Sentence reading	Regression probability	Word difficulty	Simulation	*β* = 0.285	*t*(10) = 3.23	*P* = 0.009, *R*^2^ = 0.51 (0.088 to 0.482)
Sentence reading	Regression probability	Sentence ambiguity	Human	*β* = 0.0088	*t*(8) = 5.13	*P* < 0.001, *R*^2^ = 0.77 (0.0049 to 0.0128)
Sentence reading	Regression probability	Sentence ambiguity	Simulation	*β* = 0.0289	*t*(8) = 5.39	*P* < 0.001, *R*^2^ = 0.78 (0.0166 to 0.0413)
Sentence reading	Skip probability	Sentence ambiguity	Human	*β* = −0.0025	*t*(8) = −0.46	*P* = 0.656, *R*^2^ = 0.03 (−0.0150 to 0.0100)
Sentence reading	Skip probability	Sentence ambiguity	Simulation	*β* = −0.0049	*t*(8) = −1.81	*P* = 0.107, *R*^2^ = 0.29 (−0.0110 to 0.0013)
Text reading	Regression probability	Unambiguous versus ambiguous sentences	Simulation	OR = 1.57	*χ*^2^(1) = 25.97	*P* < 0.001 (1.32 to 1.87)
Time pressure	Reading speed	Available reading time	Human	*β* = −13.25	*t*(114) = −2.87	*P* = 0.005, *R*^2^ = 0.067 (−22.38 to −4.12)
Time pressure	Reading speed	Available reading time	Simulation	*β* = −13.52	*t*(24) = −3.77	*P* < 0.001, *R*^2^ = 0.372 (−20.92 to −6.12)
Time pressure	Skip rate	Available reading time	Human	*β* = −0.041	*t*(114) = −4.09	*P* < 0.001, *R*^2^ = 0.128 (−0.061 to −0.021)
Time pressure	Skip rate	Available reading time	Simulation	*β* = −0.033	*t*(24) = −4.23	*P* < 0.001, *R*^2^ = 0.427 (−0.050 to −0.017)
Time pressure	Regression rate	Available reading time	Human	*β* = 0.136	*t*(114) = 8.15	*P* < 0.001, *R*^2^ = 0.368 (0.103 to 0.169)
Time pressure	Regression rate	Available reading time	Simulation	*β* = 0.123	*t*(24) = 6.36	*P* < 0.001, *R*^2^ = 0.628 (0.083 to 0.163)
Time pressure	MCQ accuracy	Available reading time	Human	*β* = 0.117	*t*(861) = 14.51	*P* < 0.001, *R*^2^ = 0.196 (0.101 to 0.132)
Time pressure	MCQ accuracy	Available reading time	Simulation	*β* = 0.156	*t*(24) = 2.96	*P* = 0.007, *R*^2^ = 0.267 (0.047 to 0.264)
Time pressure	Free recall	Available reading time	Human	*β* = 0.038	*t*(861) = 6.67	*P* < 0.001, *R*^2^ = 0.049 (0.027 to 0.049)
Time pressure	Free recall	Available reading time	Simulation	*β* = 0.070	*t*(24) = 1.99	*P* = 0.058, *R*^2^ = 0.142 (−0.003 to 0.143)

Each row reports the corresponding test statistic, degrees of freedom, *P* value, effect size and 95% CI where applicable. For linear regression analyses, *β* denotes the estimated slope, and *R*^2^ is reported as the effect size. For the chi-squared analysis, the OR is reported as the effect size.

Our model also replicates where to fixate. Human readers exhibit a preferred viewing position, with initial fixations landing between the beginning and the middle of the word^8,27^. Our model reproduces this spatial pattern, matching the McConkie dataset^28^, where first-fixation probabilities peak in this region. In addition, human readers exhibit within-word (intraword) regressions that return to earlier letter positions. Our model captures this effect in comparison with Rayner’s data^27^, showing higher regression probabilities towards earlier positions within the word. Both effects are illustrated for word length equals 8 in Fig. 3a. Together, the simulated initial fixation and regression distributions closely match human patterns, demonstrating that the model captures not only when but also where visual attention is allocated during word recognition.

**Extended Data Fig. 1:**
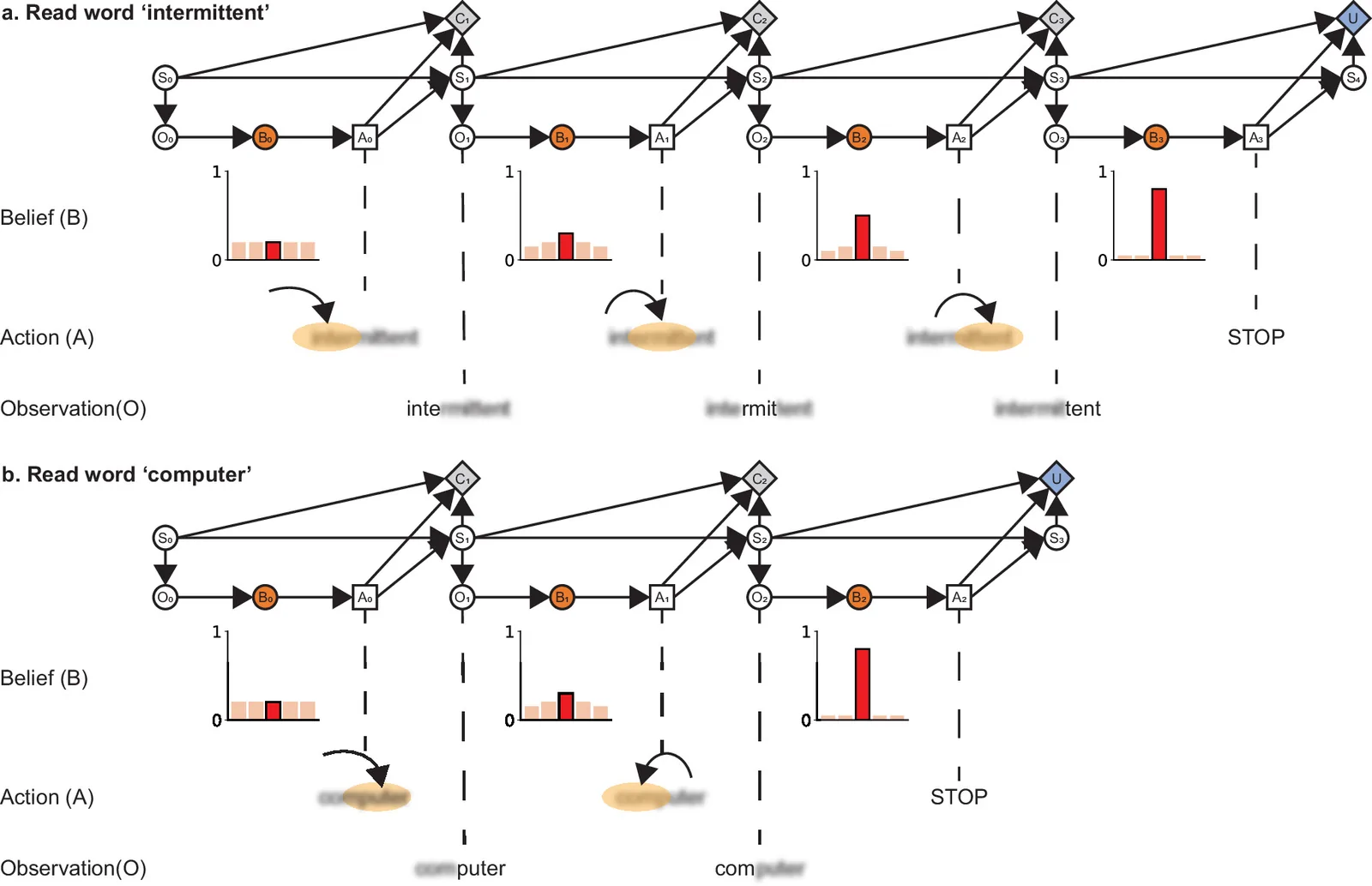
Illustration of word-recognition dynamics in the resource-rational POMDP model. At each fixation, the agent receives a noisy visual observation *O*_*t*_ (letters within the current foveal region), which updates a belief distribution over candidate words in lexical memory *B*_*t*_ (orange nodes). The bar plots show these evolving belief states: as more evidence is sampled, the probability of the correct word (red bar) increases and becomes discriminable from candidates. Based on the current belief, the agent selects an eye-movement action *A*_*t*_, either continuing to sample additional letters (and where to fixate) or terminating recognition. Frequent or predictable words require fewer fixations because their beliefs converge more quickly. Together, word recognition emerges as a boundedly optimal process of reducing uncertainty while balancing eye-movement costs *C*_*t*_ and recognition utility *U*. **a**. Reading the word intermittent: fixations proceed sequentially from left to right, and the belief over candidate words progressively sharpens, with the target word becoming increasingly dominant as more informative letters are sampled. **b**. Reading the word computer: when the initial fixation lands on a suboptimal (for example, lateral) position, the agent initiates an intraword regression to acquire additional diagnostic information, leading to a subsequent increase in confidence in the target word.

We model word recognition as a resource-rational control problem framed as a POMDP (Extended Data Fig. 1). As visual attention is limited, human readers often require multiple fixations on different parts of a word to identify it accurately. Resource rationality explains this as uncertainty reduction: for both human readers and the agent reader, the partial visual input of letters from each fixation activates several candidate words in lexical memory, creating uncertainty about the word’s identity. To reduce this uncertainty, the reader selects fixation locations that maximize expected information gain for accurate word recognition under resource constraints. We model the evolution of uncertainty using Bayesian belief updating^11^. The agent maintains a distribution over candidate words, updated after each fixation on the basis of sampled letters. Lexical frequency and contextual predictability define priors, whereas visual input provides likelihood evidence, together determining posterior beliefs. Fixation duration is modelled as proportional to uncertainty reduction: when multiple candidates remain active, fixations lengthen to resolve competition^11^. At each step, the agent chooses actions (fixation locations and termination decisions) that balance recognition accuracy against the effort and time cost of eye movements.

Compared with traditional models of eye-movement control such as E-Z Reader^8^ and the Über-Reader^17^, our approach differs in how lexical factors are mapped onto eye-movement control. In these models, variables such as word frequency, predictability and length are explicitly linked to fixation timing and control decisions through predefined processing stages or parameterized functions. By contrast, our model does not specify fixed mappings from these variables to eye movements a priori. Instead, they enter as components of the agent’s belief state (for example, priors and likelihoods over word identity), and their influence on fixation behaviour emerges from optimizing a resource-rational control policy. As a result, classic effects arise from the objective of reducing uncertainty under resource constraints, rather than being encoded as predefined control rules.

In contrast to the Bayesian reader^11^, which models word recognition as thresholded evidence accumulation with fixed information acquisition, our model treats word recognition as a sequential decision-making problem. Although Bayesian inference governs belief updating in both approaches, our model additionally optimizes action selection: at each step, the agent selects fixation locations and termination decisions to maximize expected recognition utility under cognitive and temporal constraints. As a result, both when and where to fixate are determined by a unified control objective, rather than specified by predefined rules or thresholds. Under restrictive assumptions, such as fixed sampling and threshold-based stopping, the Bayesian reader can be interpreted as a restricted instance of this formulation.

This model explains classic word-level eye-movement effects as consequences of rational uncertainty reduction during lexical identification. Longer words yield greater lexical uncertainty, requiring more and longer fixations for identification. By contrast, frequent or predictable words begin with sharper priors, which lead to more peaked belief distributions and thus reach confident recognition more quickly, resulting in shorter and fewer fixations. Notably, fixation locations are selected to maximize expected information gain under perceptual constraints, leading to a preferred viewing position slightly left of centre, consistent with human data. Within-word regressions arise when uncertainty remains after initial fixation, directing attention to earlier letter positions that are often more diagnostic for disambiguating competing candidates. In summary, word recognition is a boundedly optimal process in which fixation timing and location reflect rational trade-offs between information gain, accuracy and eye-movement costs.

### Deciding where to fixate in a sentence

At the sentence level, readers must decide not only how long to fixate on words but also whether to skip ahead or regress to earlier text to maintain coherent comprehension. Lexical properties systematically shape eye-movement control at the sentence level: shorter, more frequent and more predictable words are skipped more; difficult words elicit regressions^6,24,32,38–41^. Here, we derive word difficulty as integration difficulty quantified by surprisal (negative log probability of a word given its preceding context)^42,43^, which jointly captures frequency and contextual predictability. These effects make skipping and regression behaviour informative readouts of how readers allocate attention during sentence comprehension. We use the Task 2 of the ZuCo 1.0 dataset, which provides detailed eye-movement records during natural sentence reading^44^. We derived two complementary measures to capture these effects: skipping probability, defined as the proportion of trials in which a word received no first-pass fixation, and regression probability, defined as the proportion of trials in which a word was refixated after the gaze had already moved past it. Owing to the granularity of the available eye-tracking annotations, our analyses focus on interword (between-word) regressions; intraword regressions are not reliably distinguished in the dataset and are therefore not included in the sentence-level evaluation.

Our model reproduces these effects (Fig. 3b,c). Skipping decreases with word length (human, *β* = −0.03, *R*^2^ = 0.70; simulation, *β* = −0.02, *R*^2^ = 0.73) and increases with log frequency (human, *β* = 0.06, *R*^2^ = 0.70; simulation, *β* = 0.02, *R*^2^ = 0.74) and with logit predictability (human, *β* = 0.03, *R*^2^ = 0.41; simulation, *β* = 0.03, *R*^2^ = 0.20). Regression probabilities rise with word difficulty (human, *β* = 0.21, *R*^2^ = 0.90; simulation, *β* = 0.29, *R*^2^ = 0.51). Our model reproduced the direction of the lexical effects on sentence-level eye-movement control (Fig. 3b,c and Table 1). Across the aggregate values plotted in the figure, skipping probability decreased with word length and increased with log frequency in both human and simulated data. Skipping probability also increased with logit predictability in both datasets. Regression probability increased with word difficulty in both human and simulated data. Together, these results show that the model captures the major directional effects of lexical variables on skipping and regression behaviour during sentence reading.

Beyond lexical effects during sentence reading, we also examined sentence-level integration difficulty by analysing how sentence ambiguity influences eye-movement behaviour. Sentence ambiguity was estimated using an uncertainty-based measure reflecting the predictability of sentence continuations in context^45^ (see Supplementary Information section 1.4.2 for details). For each sentence, we computed the average skipping and regression probabilities across its words and examined how these vary with sentence ambiguity. Regression probability increased significantly with sentence ambiguity in both human and simulated data (Fig. 3b,c and Table 1), indicating that more ambiguous sentences trigger additional rereading during integration. By contrast, skipping probability showed a weak negative trend with sentence ambiguity in both datasets, but neither trend reached significance. These results suggest that the model captures the qualitative influence of sentence-level ambiguity on eye-movement control.

**Extended Data Fig. 2:**
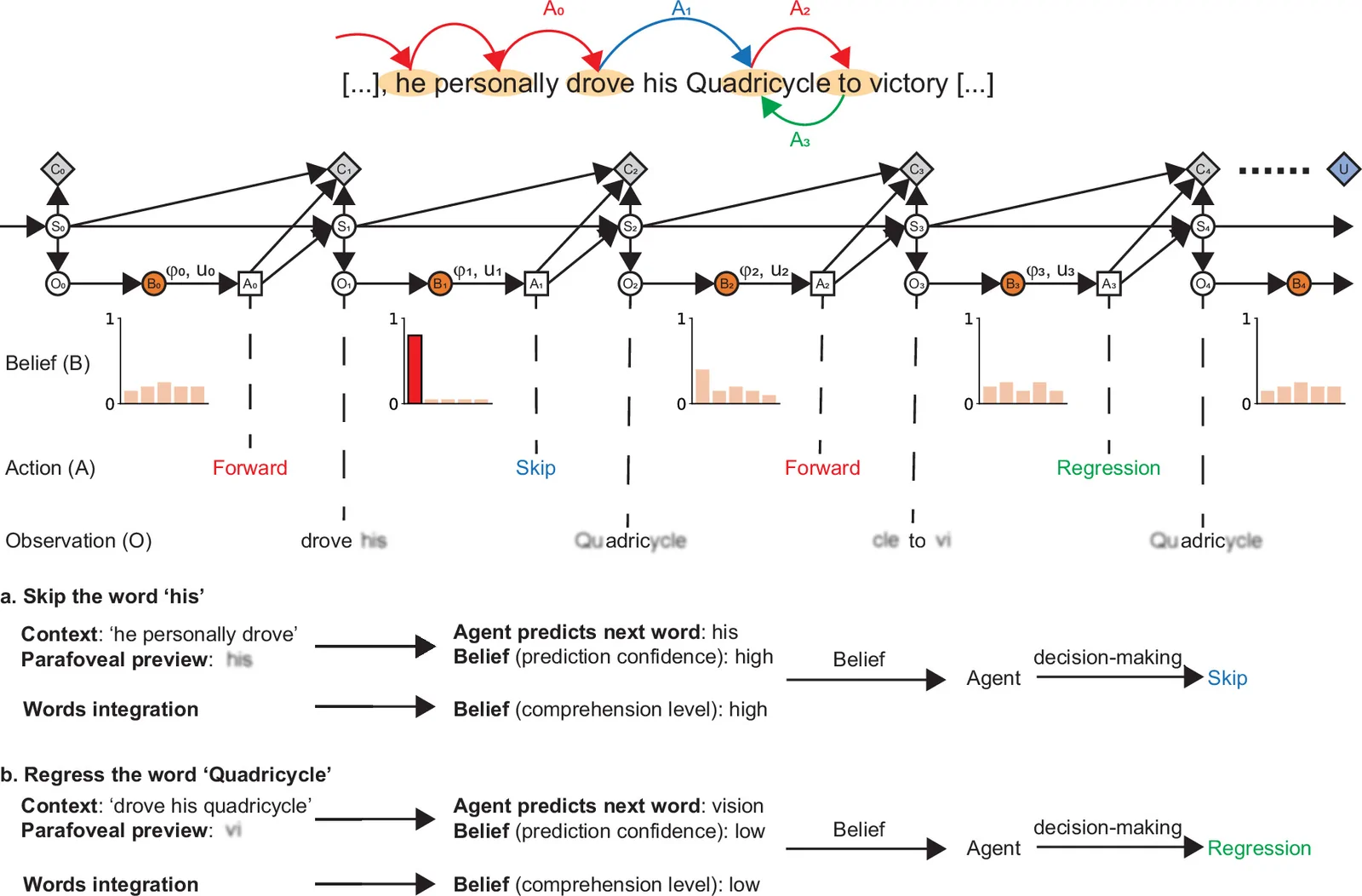
Illustration of sentence-level reading dynamics in the resource-rational POMDP model. The agent reads sentences through a combination of forward movements, skipping, regressions, and termination decisions. When reading the given sentence, it minimizes eye-movement effort and time cost *C*_*t*_ while maximizing sentence-level comprehension utility *U*, defined as *u*_*t*_, which is the geometric mean of all word appraisals *φ*_*t*_. Words that are highly predictable are skipped to save time, whereas difficult or incoherent words trigger regressions to restore comprehension. Visual input *O*_*t*_, sampled by word recognition agent, includes both foveal and parafoveal information: letters at fixation are seen clearly, while adjacent words are perceived with lower acuity. This parafoveal preview, combined with contextual cues in short-term memory, allows the agent to predict upcoming words and maintain a belief distribution over possible candidates (shown as bar charts; higher confidence indicated by red bars). Based on these beliefs, the agent strategically decides whether to skip or make a forward fixation. Regression decisions, in contrast, are guided by the estimated comprehension of the text read so far. When comprehension drops, the agent evaluates whether the expected gain from rereading outweighs the additional time and effort costs, and will regress if doing so improves overall understanding. Together, these processes demonstrate how sentence reading emerges as a boundedly optimal control of visual sampling and memory integration under cognitive and temporal constraints. **a**. Skipping the word his: given the preceding context and parafoveal preview, the agent assigns a high-probability belief to the upcoming word. Because both prediction confidence and current comprehension belief are high, skipping is expected to incur minimal risk to overall understanding while reducing time and effort costs. **b**. Regressing the word Quadricycle: when the word is difficult to integrate into the evolving sentence representation, the agent’s comprehension belief decreases. Under this low-confidence state, the expected value of re-reading the word outweighs the additional cost, leading the agent to initiate a regression to recover comprehension.

Sentence-level eye-movement control can be understood as strategic action selection under uncertainty, where readers balance comprehension goals against the costs of eye movements and time. We formalize eye-movement in sentence reading as resource-rational control under partial observability (Extended Data Fig. 2). Rather than a linear or reflexive sequence^8,46^, sentence reading is modelled as a strategic control problem in which the reader chooses among actions: moving forward, skipping, regressing or terminating to optimize expected comprehension under visual attention and memory constraints. In this formulation, the agent’s decisions are directly guided by two internal beliefs that summarize its partially observable state. A comprehension belief tracks how well previously read words have been integrated into a coherent representation, whereas a prediction belief estimates the likelihood of upcoming words on the basis of contextual information and parafoveal preview. When the comprehension belief is low, indicating poor semantic integration among previously read words (Methods), the agent increases regressions (and reduces skipping) to improve comprehension, trading additional time and eye-movement costs for information gain. This explains why ambiguous sentences and difficult words, which disrupt semantic integration, trigger more regressions. Conversely, when the prediction belief for upcoming words is high, the agent skips these words to save time with minimal expected information loss. This accounts for higher skipping probability for short, frequent or predictable words, which are more easily inferred from context and preview. Skipping is also more likely in less ambiguous sentences, where greater comprehension certainty reduces the need for verification through fixations or regressions. Importantly, although eye-movement behaviour is strongly modulated by local lexical properties, these factors do not directly determine control decisions. Instead, they influence the agent’s internal beliefs, which in turn guide action selection. Thus, lexical influences on eye movements emerge as a consequence of belief-driven control under a unified comprehension objective rather than as independent determinants of behaviour.

In contrast to models that map eye-movement behaviour to predefined control rules^8,9^, structured processing mechanisms^17^ or fixed decision thresholds^47^, our model formulates fixation control as an optimization problem. At each step, the agent selects among forward movement, skipping and regression by optimizing expected sentence-level comprehension utility under resource constraints. Lexical properties (for example, frequency and predictability) influence behaviour indirectly through the belief state, shaping action selection via the resulting control policy.

### Text comprehension and deciding where to read in text

At the level of extended text, readers must decide not only how to process incoming information but also when to revisit earlier material to maintain a coherent mental representation. Prior work has found that both text coherence (the explicit connectedness of ideas within and across sentences) and prior knowledge (readers’ existing understanding of the text domain) strongly shape comprehension and recall^7,29^. Using the dataset of McNamara et al.^29^, we replicated these effects and matched their magnitudes (Fig. 3d): participants recalled a greater proportion of propositions when they possessed higher prior knowledge (human, *M* = 0.45 ± 0.03; simulation, *M* = 0.47 ± 0.03) compared with lower prior knowledge (human, *M* = 0.34 ± 0.05; simulation, *M* = 0.31 ± 0.08). Likewise, coherent texts—those with stronger local and global connections—were remembered better (human, *M* = 0.43 ± 0.05; simulation, *M* = 0.45 ± 0.05) than low-coherence texts (human, *M* = 0.35 ± 0.06; simulation, *M* = 0.33 ± 0.10). Across the four condition-level recall means, the simulation closely matched the human pattern (mean absolute error (MAE) of 0.027, root mean squared error (RMSE) of 0.033, *r* = 0.983). Beyond reproducing mean recall scores, the model also generates adaptive eye-movement behaviour during text reading. Empirical studies show that readers initiate more regressions when sentences are temporarily misanalysed or syntactically ambiguous^32,33^. To evaluate whether the model reproduces this effect, we compared regression probabilities between ambiguous and unambiguous texts using the same stimuli and condition distinctions reported by Staub and Clifton^33^. Consistent with the empirical pattern, the agent exhibited higher regression probabilities when processing ambiguous material than unambiguous material (simulation, *M* = 0.13 ± 0.01 versus *M* = 0.09 ± 0.01; *χ*^2^(1) = 25.97, *P* < 0.001 OR 1.57, 95% CI 1.32 to 1.87), paralleling the human data^33^ (human, *M* = 0.19 ± 0.05 versus *M* = 0.07 ± 0.03), as shown in Fig. 3d and Table 1.

**Extended Data Fig. 3:**
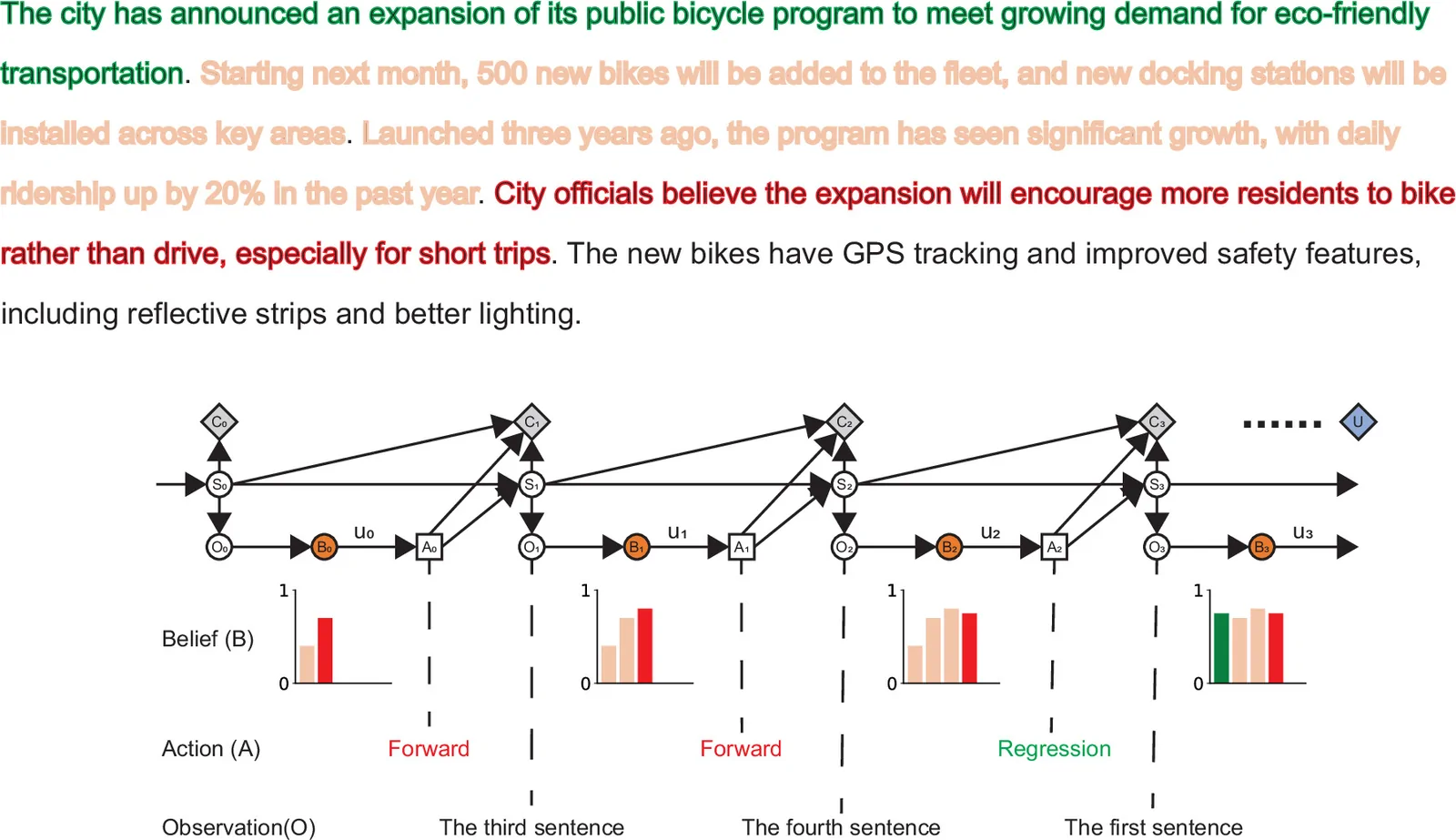
Illustration of text-level reading dynamics in the resource-rational POMDP model. When reading multi-sentence text, the agent decides whether to proceed to the next sentence (red), regress to an earlier sentence (green), or terminate reading. These decisions are governed by two interacting components. (1) Sentence appraisals *B*_*t*_, quantify how well each sentence integrates into the evolving schema-guided long-term memory; bar charts show the belief distribution over appraisals, with higher values indicating stronger contributions to coherence. (2) Text-level comprehension *u*_*t*_, is computed as the geometric mean of all processed sentence appraisals. When comprehension of a newly read sentence falls below expectation, the agent strategically regresses to reread earlier sentences. Such revisits trigger additional processing cycles that strengthen relevant propositions in memory, raising their appraisal scores and improving global coherence. These dynamics illustrate how reading decisions arise from the continuous optimization of overall comprehension utility u against the time and effort costs of eye movements *C*_*t*_, it also demonstrates the mutual effect between eye-movement control and memory construction in text comprehension.

At the text level, comprehension is governed by resource-rational control over an evolving, partially observable text state (Extended Data Fig. 3). As the agent cannot directly observe the full underlying state of the text, it must rely on partial and memory-limited observations, derived from noisy letter sampling, capacity-constrained sentence integration and compressed long-term representations. Thus, the agent must rely on a belief state that tracks the reader’s evolving interpretation of the text. At each step, the agent strategically chooses from the action space of continue reading, regress to an earlier sentence or terminate to optimize comprehension. Memory construction is modelled to parallel human processes inspired by the framework of Kintsch et al.^7^: readers build coherence across sentences by coordinating short-term and long-term memory, but because working memory is capacity-limited, only a subset of propositions can be retained. Readers therefore prioritize propositions that are coherent or important while activating relevant knowledge from long-term memory to support integration. Propositions that persist across cycles are gradually consolidated into compressed ‘gist’ representations, forming the backbone of text-level understanding. The effect of prior knowledge is captured through the model’s activation threshold, which determines how relevant a proposition must be to be integrated into long-term memory. Higher thresholds indicate greater difficulty of activation, corresponding to readers with lower prior knowledge. In contrast to prior work, the effect of text coherence does not require a threshold parameter: when the agent parses texts of varying coherence, the computed proposition-to-proposition relevance naturally differs: coherent texts yield higher internal consistency, whereas incoherent texts produce lower relevance scores. Thus, coherence effects emerge intrinsically from the text structure itself.

Compared with prior models of text comprehension and integrative reading architectures, such as Über-Reader^17^ and the construction–integration framework^7^, our model treats comprehension as an explicit control objective that directly guides eye-movement decisions. In these models, comprehension is constructed through interactions among memory representations and processing mechanisms but is not used to explicitly select actions. By contrast, eye movements in our model are selected by optimizing a unified objective over expected comprehension outcomes under memory constraints. Consistent with the regression patterns observed in both human readers and the model (Fig. 3d), when integration difficulty or ambiguity arises, readers deploy targeted regressions as compensatory control^32,38^, strategically revisiting earlier text to restore coherence. In our model, such regressions are not reactive noise but rational investments, where costly actions are taken only when the expected gain in comprehension outweighs the time and effort cost. This mechanism naturally explains why rereading increases under low prior knowledge or low coherence: when memory integration falters, the optimal policy favours revisiting earlier material to recover coherence. Unlike comprehension-only frameworks^7,14,15^, which assume input is already encoded, our account links gaze allocation directly to memory integration, providing a principled computational explanation of how attention control and text-level comprehension are bidirectionally coordinated under resource constraints.

### Speed-accuracy trade-off when reading under time pressure

**Fig. 4:**
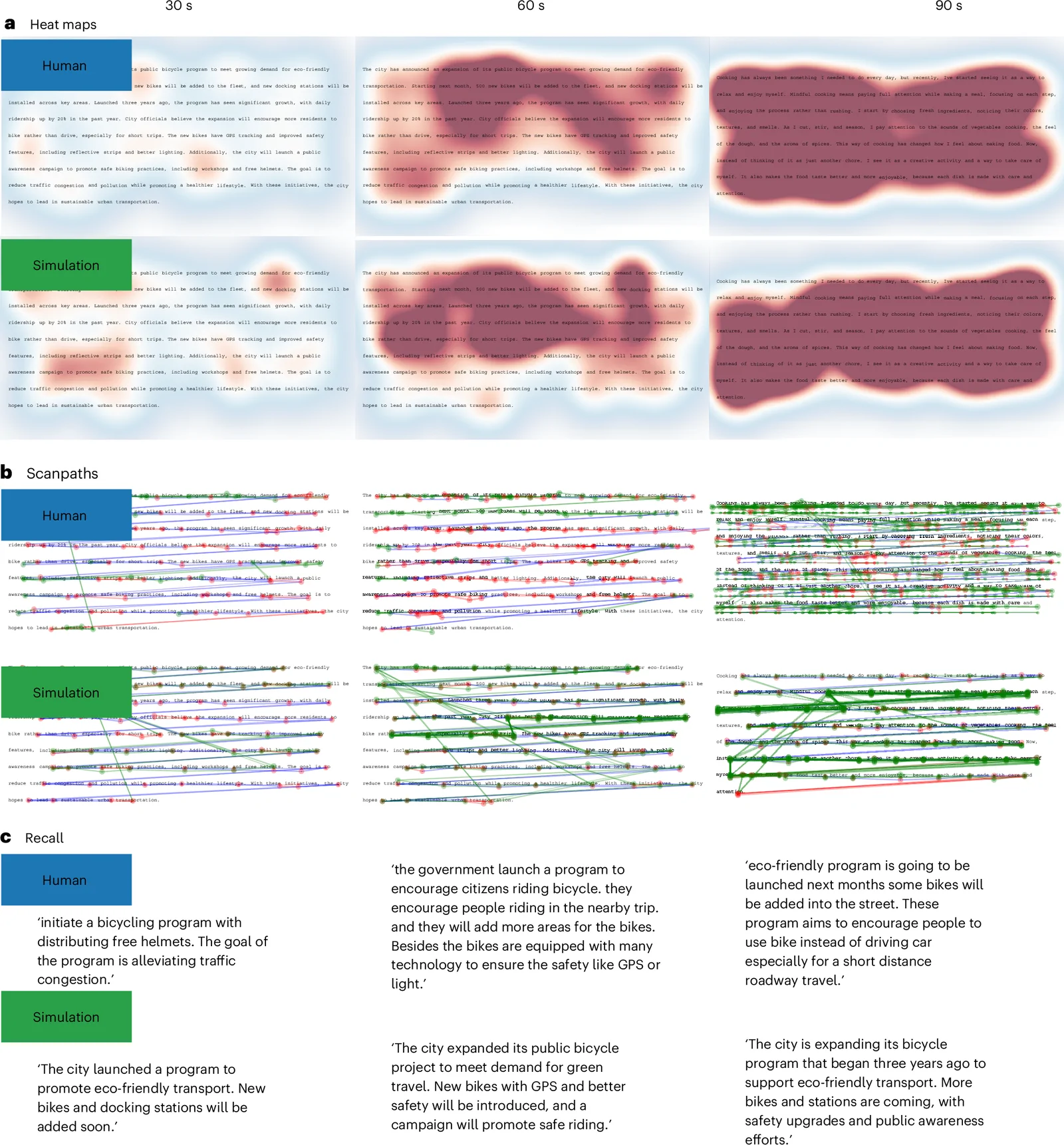
Reading behaviour under different time constraints (30 s, 60 s and 90 s). **a**, Heat maps. Both humans and the simulation broaden the coverage of fixations and allocate more fixation time as available reading time increases. **b**, Scanpaths. Eye-movement sequences adapt to time pressure: limited time causes faster reading with more skips (blue) and fewer regressions (green), whereas abundant time allows more careful inspection and targeted regressions. Red dots and lines represent normal (first-pass) fixation points and saccades, respectively. **c**, The recall. Free-recall responses reflect the same trade-off: shorter durations yield coarse recall, whereas longer durations support more complete and detailed memory. Together, these patterns illustrate reading as a resource-rational control process that flexibly adapts to time resources: when time is limited, readers optimize overall comprehension by prioritizing coverage over detail—skipping more and regressing less; when time is abundant, they shift towards higher-accuracy comprehension by investing additional fixations and regressions.

**Extended Data Fig. 4:**
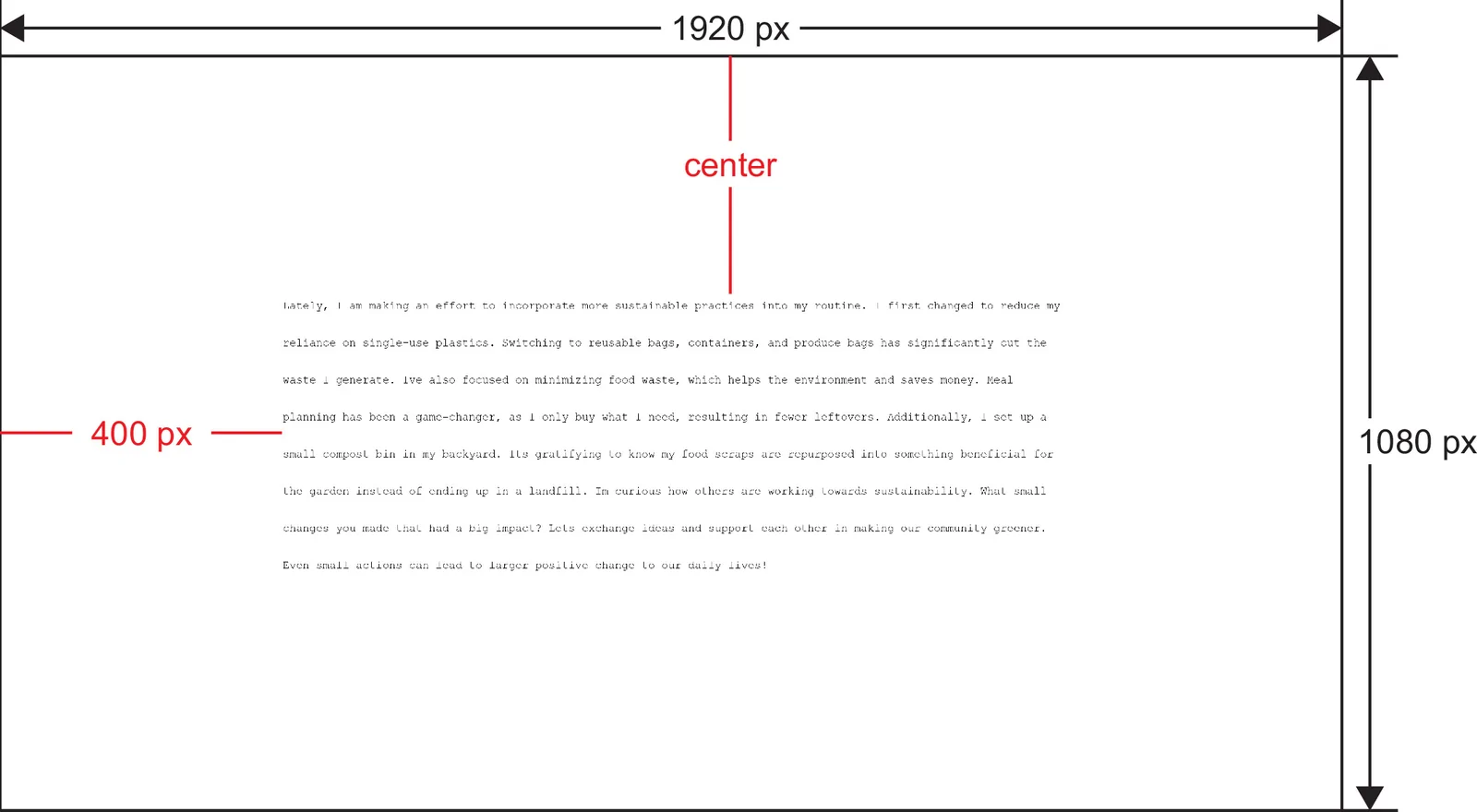
Stimulus presentation. Reading stimuli were displayed at the Tobii eye-tracker monitor. Centering the text minimizes distortions and reduces measurement error in marginal regions, thereby improving eye-movement data quality.

Time pressure fundamentally alters how humans read, forcing readers to trade comprehension accuracy against speed. Prior studies have established that when readers face time constraints, they adjust reading strategies: reading speeds increase, skipping becomes more frequent, regressions are reduced and comprehension performance declines^35^. However, most existing datasets either do not manipulate time directly or fail to capture the joint dynamics of gaze behaviour and memory formation during English reading. To address this gap, we collected a dedicated *Reading Under Time Pressure* dataset, in which 39 participants read texts (stimulus exemplified in Extended Data Fig. 4) under three time conditions (30 s, 60 s and 90 s) whereas both eye movements and comprehension outcomes were simultaneously recorded. Human readers exhibited systematic adaptations across time conditions (Fig. 3e). As available reading time increased from 30 s to 90 s, reading speed decreased from 191 ± 38 to 166 ± 28 words per minute (WPM), skipping rate declined from 0.39 ± 0.08 to 0.31 ± 0.08 and regression rate increased from 0.32 ± 0.12 to 0.59 ± 0.11. Comprehension performance also improved with more time, with multiple-choice question (MCQ) accuracy increasing from 0.59 ± 0.14 to 0.83 ± 0.12 and free recall increasing from 0.64 ± 0.08 to 0.73 ± 0.07. Example scanpaths and heat maps under the three time conditions are shown in Fig. 4a,b, and example free-recall responses are shown in Fig. 4c. The simulation reproduced the same directional adaptations across reading speed, skip rate, regression rate and MCQ accuracy, with the free-recall trend in the same positive direction but not reaching conventional significance (Table 1). Across the 15 metric-by-time-condition means, human–simulation agreement was high (MAE of 1.460, RMSE of 3.305, *r* = 0.9999). From a resource-rational perspective, these adjustments reflect a strategic trade-off of comprehension gains against time costs. When time is abundant, readers can afford to allocate more fixations to difficult texts and to regress to repair comprehension. As time becomes scarce, however, the value of additional fixations diminishes: forward saccades accelerate, regressions are suppressed and attention shifts towards selectively sampling more informative regions.

To capture these empirical adaptations, we extended the model to incorporate time constraints, framing comprehension as a resource-rational control problem where speed must be traded against comprehension performance. Unlike natural, unconstrained reading, time-pressured reading forces agents to prioritize task completion within strict temporal limits, often sacrificing local (sentence-level) or global (text-level) coherence. Our model incorporates time directly into the observation space, allowing the agent to track remaining time and adjust its behaviour accordingly. Final rewards are computed on the basis of comprehension score, which is the geometric mean of appraisals across sentences, shaped by time-sensitive factors that influence the agent’s priorities under pressure (see the Methods for details). This enables the agent to learn adaptive reading strategies, such as modulating skipping, regressions and termination, on the basis of available time. The enhanced model reproduces and explains key empirical effects (Fig. 3e): with ample time (90 s), the agent allocated more fixations to difficult or low-confidence regions, often revisiting earlier sentences to strengthen integration. Under high time pressure (30 s), both humans and the model adopt a quantity-over-quality strategy, characterized by rapid forward saccades, minimal rereading and selective sampling of informative regions. This shift mirrors human adaptation across conditions and reflects a strategic reweighting of expected comprehension gains against time costs: as time becomes scarce, completing the text efficiently is prioritized over further refinement of understanding.

**Extended Data Fig. 5:**
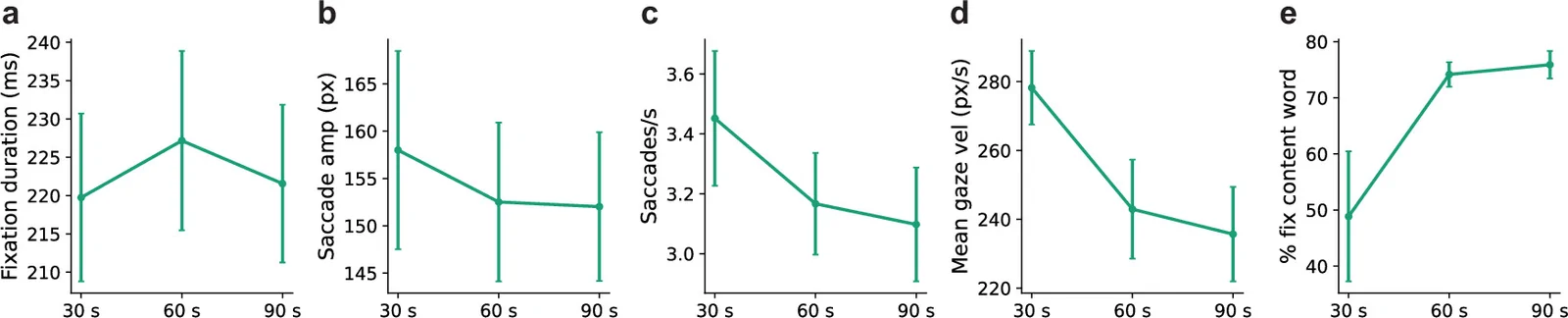
Simulation effects on fixation and saccade-related metrics. a–e, Mean fixation duration, saccade amplitude, number of saccades per second, mean gaze velocity and percentage of fixations landing on content words. Each panel shows mean values standard deviation across three time-constraint conditions (30 s, 60 s, 90 s). Error bars represent between-episode variability within the simulated data. As time pressure decreased, fixation duration slightly lengthened, saccade amplitude and gaze velocity decreased, and the proportion of fixations landing on content words increased. These trends mirror the qualitative patterns reported in the new dataset, even though our model was evaluated on different English stimuli and time constraints. The comparison therefore concerns the direction and relative shape of the effects, not the specific dataset or materials, demonstrating that the simulated control policies reproduce key signatures of human reading adaptation under varying time constraints.

Apart from matching these empirical metrics, our model also captured the direction of effects reported in an independent eye-tracking study of reading under time constraints on a French corpus^35^. That study reported human adaptations consistent with our findings: as time pressure decreases, readers show longer mean fixation durations and a higher proportion of directly fixated content words, whereas saccade amplitude, saccade rate and left-to-right gaze velocity decline. Our model reproduced all of these time-pressure-dependent patterns to a high level of accuracy (Extended Data Fig. 5).

### Validating the necessity of hierarchical resource rationality

**Fig. 5:**
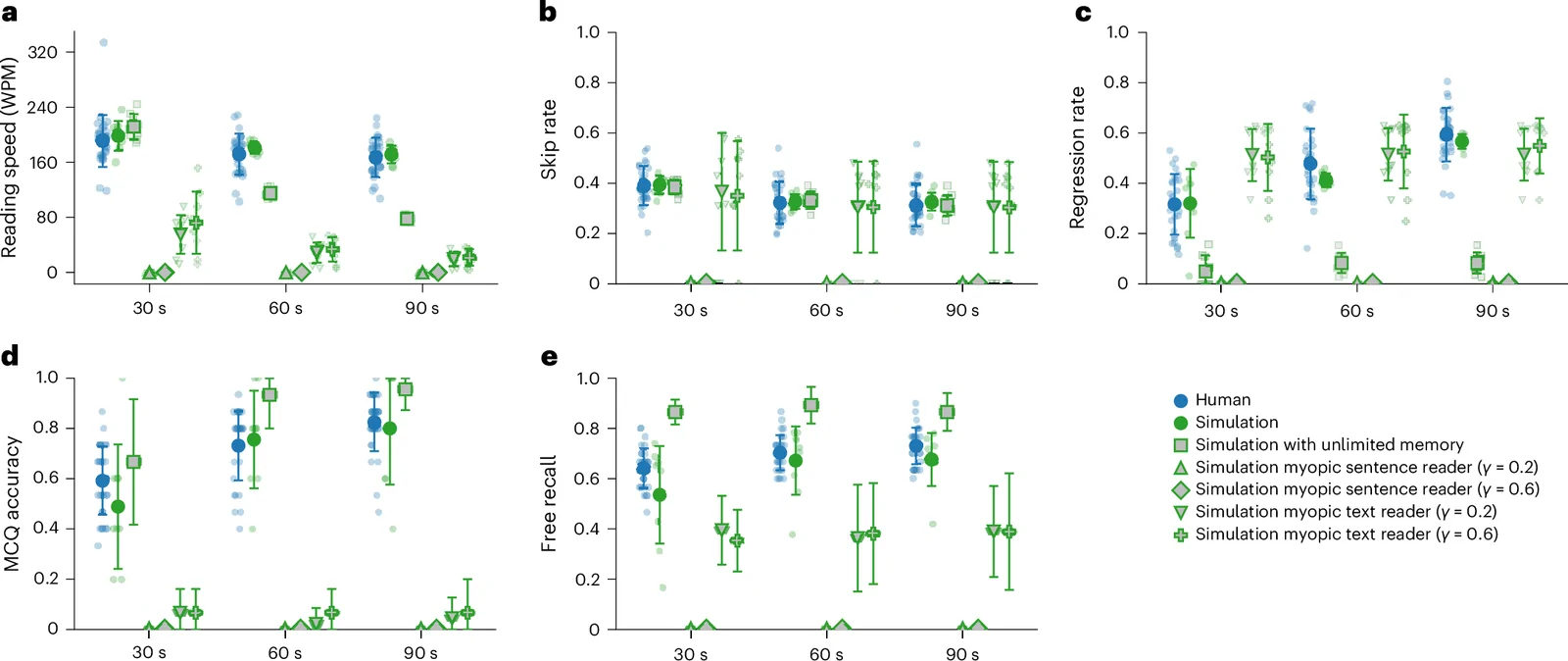
Effects of memory limits, hierarchical integrity and long-term comprehension utility. **a**–**c**, Eye movements (reading speed (**a**), skip rate (**b**) and regression rate (**c**)) under three time limits (30 s, 60 s and 90 s). **d**,**e**, The comprehension (MCQ accuracy (**d**) and free recall (**e**)) under three time limits (30 s, 60 s and 90 s). Human data are shown in blue; the full model simulation is shown in green; baseline simulations are shown in grey with green outlines/hatches. Dots show individual observations, with human eye-movement measures plotted at the participant level (*n* = 28), human comprehension measures plotted as participant-level means across trials (*n* = 32) and simulation eye-movement and comprehension measures plotted at the episode–stimulus level for the full model (*n* = 9 per time condition). Baseline eye-movement measures are plotted at the episode–stimulus level (*n* = 9 per time condition). Baseline comprehension summaries were computed over (*n* = 27) trials per time condition. Large markers show means, and error bars denote standard deviations; for bounded measures (skip rate, regression rate, MCQ accuracy ND free recall), error bars are truncated to the feasible range ([0, 1]). The unlimited-memory model achieves markedly superhuman MCQ and free-recall scores while showing fewer regressions and faster reading. The myopic sentence reader (*γ* = 0.2, 0.6) collapses into local behaviour, repeatedly fixating early words and failing to advance through the text, producing near-zero comprehension. The myopic text reader ((*γ* = 0.2)) preserves lower-level word and sentence processing but fails to proceed reading over sentences, yielding markedly reduced comprehension; a milder myopia ((*γ* = 0.6)) improves performance but still falls far below humans. By contrast, the full agent, combining bounded memory with non-myopic optimization of future comprehension, most closely matches human eye movements and comprehension. These results show that human-like reading requires realistic resource limits, sensitivity to long-term comprehension utility and an intact hierarchical control structure.

To further validate the memory-construction module design and necessity of long-term comprehension utility optimization, we compared the full hierarchical model with five baseline models (Fig. 5). To quantify model adequacy, each model was compared with the human data across the 15 metric-by-time-condition means shown in Fig. 5: reading speed, skip rate, regression rate, MCQ accuracy and free recall across 30 s, 60 s and 90 s. The full hierarchical model showed the closest agreement with human behaviour across these measures (Pearson *r* = 0.9999, MAE of 1.46, RMSE of 3.31), whereas all ablated models produced substantially larger deviations from human behaviour (rRMSE of 27.52–78.96).

The first baseline, the unlimited-memory agent, stores all parsed propositions in long-term memory without strategic selection. It achieved superhuman comprehension performance, with higher mean MCQ accuracy and free recall than humans across time conditions (MCQ, 0.852 versus 0.716; free recall, 0.874 versus 0.692), while producing markedly fewer regressions (0.072 versus 0.462). Although its overall pattern remained positively correlated with human data (*r* = 0.9324), it deviated much more strongly from human behaviour than the full model (MAE of 11.12, RMSE of 27.52), indicating that realistic memory limits are essential for generating human-like reading behaviour. The next two baselines were myopic sentence readers (*γ* = 0.2, 0.6), which plan only locally within each sentence and fail to optimize longer-term sentence- and text-level comprehension. These agents failed to learn forward progress, producing near-zero comprehension and abnormal eye-movement profiles. Their outputs therefore showed the largest deviations from human behaviour (MAE of 35.69, RMSE of 78.96 for both variants), consistent with stalling at local states rather than pursuing a longer-term reading goal. The final two baselines were myopic text readers (*γ* = 0.2, 0.6), which preserve lower-level reading mechanisms but lack efficient high-level planning over the text. These agents read only the initial portions of the text and failed to decide where to read next to maximize overall comprehension. As a result, they showed substantially slower reading than humans (mean reading speed, 34.36 and 42.31 WPM, respectively, versus 176.25 WPM in humans) and markedly reduced comprehension (mean MCQ accuracy, 0.044 and 0.067, respectively, versus 0.716 in humans; mean free recall: 0.383 and 0.375, respectively, versus 0.692 in humans). Both myopic text-reader variants therefore deviated much more strongly from human behaviour than the full model (MAE of 28.60, RMSE of 63.49 for (*γ* = 0.2); MAE of 27.00, RMSE of 60.11 for (*γ* = 0.6)).

Across all conditions, only the full hierarchical model, combining bounded memory capacity with long-term value estimation, reproduces both human eye-movement patterns and comprehension performance. Ablation results show that removing higher-level planning leads to inefficient reading behaviour, such as stalling or premature termination, because lower-level processes lack guidance about future consequences. By contrast, the full model selects actions on the basis of their expected contribution to overall comprehension, allowing higher-level objectives to guide eye-movement decisions without requiring explicit coordination mechanisms.

## Discussion

This Article establishes that human reading behaviour arises from a general resource-rational control mechanism—one that coordinates perception, memory and sequential action under uncertainty. Although deep reinforcement learning has been advanced as a powerful method for instantiating computational theories of the human mind^48–53^, what has been missing is an account of how hierarchical processing emerges in knowledge-intensive tasks involving eye movements. Readers allocate fixations through strategic, sequential decisions that maximize comprehension while minimizing effort and time, under the uncertainties imposed by limited cognitive resources. Our model represents this principle as a hierarchical POMDP solved with deep reinforcement learning, enabling policies to emerge under realistic perceptual and memory uncertainty that faithfully capture human-like resource limits. This resource-rational hierarchical architecture bridges a long-standing divide among theories of memory, perception and control, not only in reading research but also across behavioural and cognitive sciences. It proposes that high-level comprehension processes and lower-level motor-control processes can be understood as outcomes of a unified optimization process. Although integrative architectures such as the Über-Reader^17^ aim to link eye-movement control with comprehension across multiple levels, providing a detailed and empirically grounded account of interacting processes, control in these models is specified through the structure and interaction of processing components. By contrast, our model formulates reading as a sequential decision-making problem, in which eye-movement behaviour and comprehension emerge from learned policies that optimize a shared objective under uncertainty. Unlike earlier models^8,9^ that relied on handcrafted heuristics or treated gaze behaviour as reflexive, our approach treats reading as an adaptive, goal-directed process of active information sampling. As a result, the model reproduces canonical behavioural phenomena across scales—from word to sentence to text—including effects of word length, frequency and predictability, as well as skips, regressions and adaptive strategies under time pressure. More importantly, it provides direct computational validation that explaining reading mechanisms through the lens of resource rationality offers a unified and general account of human reading behaviour. The same trade-off between immediate and delayed informational gain appears broadly in human decision-making, suggesting that reading reveals a general cognitive strategy for allocating effort and information under uncertainty, rather than a task-specific mechanism.

Resource rationality explains the remarkable adaptability observed in human reading. When we relaxed resource limits in simulation by granting the agent unlimited memory or full visual access to text, strategic eye-movement control disappeared. The agent no longer needed to decide where to look or when to move; comprehension could be achieved trivially without selective sampling. Conversely, when resources were bounded, the agent adapted by allocating fixations to maximize long-term comprehension utility while minimizing immediate effort. This trade-off was formalized through value estimation across future steps: when decision-making was biased towards short-term outcomes (*γ* = 0.2, 0.6), the agent became myopic and locally optimal, fixating repeatedly within a sentence without anticipating how present sampling supports later comprehension. These behavioural shifts mirror human tendencies to balance immediate versus delayed informational gain, reinforcing that reading is a goal-directed optimization process. The hierarchical architecture of our model was crucial for enabling such adaptive control. Human readers naturally decompose the complex task of understanding text into nested subtasks^6,8^—selecting regions, words and letters—each operating over distinct temporal and informational scales. Such hierarchical decomposition is a signature of many complex skills^54^: from language comprehension to motor planning, suggesting the mechanisms uncovered here extend beyond reading. When the hierarchical structure was disrupted (for example, by removing or collapsing any of the three levels), the agent failed to learn effective policies: long-term comprehension goals alone were too sparse and insufficient to guide moment-to-moment perceptual decisions without intermediate control levels. The need for intermediate controllers illustrates how hierarchical control provides both efficiency and learnability under bounded resources. This mechanism parallels neural evidence that reading engages fast hierarchical processing of orthographic and semantic information across distributed cortical regions^21,22^.

Our approach yields clear, testable predictions about how reading behaviour should vary across individuals, populations and contexts as a function of underlying resource constraints and valuation. Differences between native and non-native readers should primarily reflect weaker lexical and predictive knowledge, leading to reduced skipping and slower integration even for frequent or predictable words^55^, whereas dyslexic reading should instead reflect increased perceptual and decoding uncertainty, producing longer fixations and more regressions even when predictability is high^56^. Beyond group-level effects, stable individual differences in fixation durations, skipping and rereading should be explainable by continuous variation within a shared control architecture rather than distinct strategies. Situational factors such as time pressure^35^ or multitasking^49,52^ should modulate reading behaviour by reweighting the relative value of comprehension versus efficiency, yielding predictable person-by-situation interactions. These predictions can be evaluated by fitting reader-specific constraints within a computational model^57^, offering a unified approach to studying proficiency, impairment and adaptation in reading.

Beyond modelling differences across readers, our approach can also be extended to different reading tasks. In our model, task demands are expressed through the reward function that defines the agent’s objective. For comprehension-driven reading (the primary focus of this work), rewards are tied to comprehension quality. However, alternative tasks can be specified by redefining the reward function. For example, in proofreading, rewards would be associated with detecting linguistic errors, whereas in visual search tasks (for example, locating a specific word), rewards would depend on successfully identifying the target item. Importantly, the same resource-rational control principle applies across tasks: differences in behaviour arise not from distinct mechanisms but from differences in the reward function that defines what counts as success. This formulation allows different tasks to be modelled without introducing task-specific or handcrafted control rules, as behaviour adapts through changes in the objective rather than changes in the control mechanism. In addition, the relative importance assigned to comprehension can be modulated through weighting parameters in the reward function^49^. These parameters capture variations in task demands as well as intrinsic factors such as reader motivation. When comprehension is highly valued, the agent adopts more careful, information-seeking strategies; when its value is reduced, behaviour shifts towards faster, more effort-efficient reading that tolerates partial understanding.

This work has several limitations. First, the model is intended to explain general cognitive mechanisms of reading at the group level, rather than to provide a fully individualized account of every reader. Readers differ in linguistic background, reading proficiency, domain knowledge, goals, preferences and idiosyncratic strategies, and such differences may lead to systematic variation in fixation allocation, regression behaviour and comprehension outcomes. In the present work, these differences are captured only partially through model parameters. Future work could therefore fit parameters at the individual level and test whether the inferred parameters explain stable reader-specific traits and task-dependent strategies. Second, although the reading-under-time-pressure study provides a controlled test of how readers adapt under limited time, the empirical coverage remains limited. Future studies should evaluate the model across a wider range of genres, text lengths, difficulty levels, reading goals, populations and task contexts. Third, comprehension in the present evaluations is operationalized mainly through recall- and accuracy-based measures. These measures capture important aspects of reading comprehension, but real-world reading involves richer goals, including learning, aesthetic engagement, affective response, information search, decision-making and long-term retention. Future work could therefore combine eye-tracking with more diverse behavioural and cognitive measures.

Our computational basis offers a plausible blueprint for complex intelligent behaviour beyond reading. Many everyday activities, such as writing, sports and skill learning, all require the coordination of high-level reasoning and decision-making with low-level perceptual-motor execution. Hierarchical control supported by multilayer memory systems provides a computational solution to such multilevel interplay. By adjusting these memory systems and applying realistic perceptual-motor constraints, the same resource-rational principles can explain a wide range of intelligent activities, offering cognitive science with a unified computational basis for understanding human behaviours across these tasks. Deep reinforcement learning further implements and enhances these principles by enabling generalizable control policies in stochastic and uncertain environments. Earlier models relied on handcrafted rules that were brittle to context or agent changes, whereas amortized training using deep reinforcement learning across varied conditions yields adaptive policies that capture human-like variability without manual tuning. This flexibility opens new opportunities for systematic evaluation of cognitive and behavioural theories in fields such as human–computer interaction, education and psychology. For artificial intelligence, the model demonstrates how the learning of human-like behaviour can emerge from decision-making rather than the imitation of massive datasets. Grounding policy learning in hierarchical POMDPs integrated with cognitive theories provides a top-down route for building interpretable agents capable of reasoning, perceiving and acting with human-like efficiency and flexibility.

## Methods

### Datasets

The research complied with all relevant ethical regulations for research involving human participants. For the newly collected reading-under-time-pressure dataset, all participants were adults, provided informed consent before participation and consented to the use and sharing of anonymized data for scientific purposes. The study used a low-risk behavioural eye-tracking and reading-comprehension protocol and did not involve deception, clinical intervention, vulnerable populations, biological or genetic material or collection of personally identifying sensitive information. At the time of data collection at HKUST Guangzhou, the study was conducted in accordance with the applicable local institutional practice for low-risk behavioural research; a separate prospective review by an institutional review board or ethics committee was not obtained for this dataset. For previously published datasets, we used only data or summary statistics reported in the original publications or made publicly available by the original authors.

We validated our model across eight human reading datasets spanning word recognition, sentence reading, text comprehension, ambiguity-driven regressions and reading under time pressure. The word-recognition benchmarks included the Potsdam Sentence Corpus eye-tracking dataset of Kliegl et al.^24^, Rayner’s dataset on intraword regression locations^27^ and the dataset of McConkie et al.^28^ on initial fixation locations. Sentence-level reading was evaluated using ZuCo 1.0, Task 2 Normal Reading^44^. Text-level comprehension was evaluated using the text-coherence and prior-knowledge dataset described by McNamara et al.^29^. Regression behaviour during syntactic reanalysis was evaluated using Staub and Clifton’s syntactic ambiguity dataset^33^. Time-pressure effects were evaluated using both the French time-constrained reading dataset of Vibert et al.^35^ and a new English reading-under-time-pressure dataset collected for the present study.

For the previously published datasets, participant information is reported as described in the original publications. The Kliegl et al. dataset^24^ included 65 native German participants: 33 young adults (mean age of 21.9 years, s.d. of 2.2, range 19–28 years) and 32 older adults (mean age of 69.9 years, s.d. of 5.9, range 54–83 years); participant sex and informed-consent procedures were not reported, and participants were paid the equivalent of 5 € per hour or received course credit. Rayner’s dataset^27^ included eight college-age participants; participant sex, exact age, informed-consent procedures and compensation were not reported. The dataset of McConkie et al.^28^ included 66 college-student participants; participant sex, exact age, informed-consent procedures and compensation were not reported. ZuCo 1.0 included 12 healthy, right-handed adult native English speakers (five female, seven male; age range of 22–54 years, mean age of 38 years); participants gave written consent for participation and data re-use, the study was approved by the Ethics Commission of the University of Zurich and compensation was not reported^44^. The dataset of McNamara et al.^29^ was based on experiment 2 of the original publication and included 56 participants aged 10–15 years (mean age of 13 years 11 months); participant sex and informed-consent procedures were not reported, and participants were paid US$10. Staub and Clifton’s dataset^33^ included 24 University of Massachusetts Amherst students who were native speakers of American English; participant sex, age and informed-consent procedures were not reported and participants received course credit or were paid US$5. The dataset of Vibert et al.^35^ included 30 young adult university students (16 female, 14 male; mean age of 21.9 years, s.d. of 3.4 years); all participants gave written informed consent, the study was conducted in accordance with the Declaration of Helsinki and European General Data Protection Regulation under Université de Poitiers agreement number 202342, five participants received course credit and compensation for the remaining participants was not reported.

#### Reading under time pressure dataset (English)

To evaluate whether our model could reproduce human eye movements, memory and comprehension patterns under varying time constraints, we required an English reading dataset that extended beyond isolated sentences and included both eye-tracking and comprehension measures with explicit time manipulation. As no existing corpus met these requirements, we conducted a dedicated behavioural study that systematically varied available reading time (30 s, 60 s and 90 s) while recording both gaze behaviour and comprehension performance. Detailed descriptions of the datasets are provided in the Supplementary Information sections 1.1.1–1.18.

We recruited 39 adult participants. After data-quality exclusions, 32 participants were retained for comprehension analyses and 28 participants were retained for eye-movement analyses. The 32 participants retained for comprehension analyses included 19 female and 13 male participants (mean age 24.0 years, s.d. of 3.7, range 18–34 years). All participants provided informed consent before the experiment and received food coupons worth 50 RMB. The study used a within-subject design manipulating available reading time across three conditions: 30 s, 60 s and 90 s. On each trial, participants silently read a short text, whereas their eye movements were recorded using Tobii Pro Lab (v1.217.49450) with a Tobii Pro Spectrum eye tracker sampling at 1,200 Hz^58^. Participants then completed a 20-s arithmetic distractor followed by a comprehension test consisting of free recall and five multiple-choice questions. Texts were generated to minimize prior-knowledge confounds, and comprehension items were sanity-checked to avoid non-content cues. Fixation and saccade events were identified using Tobii’s I-VT filter^59^. We then applied a post hoc line-alignment correction using Fix8^60^, including time-warping and chain filters, to improve fixation-to-line mapping. Data preprocessing and analysis were conducted using Python (≥3.10) with standard scientific libraries, including NumPy, SciPy and pandas. From these data, we computed fixation-, saccade- and comprehension-based metrics used to evaluate the model under the same time constraints. Full details on data collection, preprocessing and metric definitions are provided in Supplementary Information sections 1.1.7 and 1.2, and all materials and prompts are provided for reproducibility.

### Model

The model is designed to investigate three core assumptions about human reading: (1) cognitive resources for perception, memory and time are limited; (2) reading unfolds under uncertainty about word identity, sentence meaning and text coherence; and (3) readers act to optimize comprehension utility subject to these constraints. Crucially, the model does not assume fixed heuristics, handcrafted decision rules or task-specific thresholds for skipping, regression or rereading. Instead, all eye-movement and rereading behaviours emerge from optimizing expected utility under partial observability and resource costs.

Specifically, human reading was modelled as a three-level resource-rational controller comprising a word recognizer, sentence reader and text reader. Each level implements a reinforcement learning policy that selects actions to maximize expected utility under cognitive and temporal costs. The controllers operate at distinct temporal scales: within-word fixations, sentence-level eye-movement control and text-level rereading decisions, which are composed using a call-and-return execution structure.

Each controller *ℓ* ∈ {*W*, *S*, *T*} is formalized as a POMDP, specified by a tuple 〈*S*_*ℓ*_, *A*_*ℓ*_, *T*_*ℓ*_, *O*_*ℓ*_, *r*_*ℓ*_, *γ*_*ℓ*_〉, where *S*_*ℓ*_ denotes the state representation, *A*_*ℓ*_ the action space, *T*_*ℓ*_ the transition dynamics, *O*_*ℓ*_ the observation model, *r*_*ℓ*_ the level-specific reward function and *γ*_*ℓ*_ the discount factor (default 0.99). Belief states for partially observable levels are updated using Bayesian update and memory construction. Higher-level actions (sentence- and text-level decisions) unfold over variable durations determined by the lower-level controller and are evaluated using a hierarchical value function. This structure allows each controller to optimize its own resource-rational objective while contributing to overall reading performance. Intuitively, at each level the model captures reading as a process of acting under uncertainty. The agent does not directly know the true identity of words or the correct interpretation of the text but instead maintains uncertain beliefs on the basis of noisy visual input and context. At each step, it must decide whether to gather more information (for example, by fixating or regressing) or to move forward, balancing the expected improvement in comprehension against the cost of time and effort. As a result, behaviours such as additional fixations, skipping or regressions emerge naturally depending on the level of uncertainty and the expected value of further processing. Detailed derivations and modelling assumptions are provided in Supplementary Information section 1.3, and full implementation details are available in the code repository.

#### Word recognizer

At the lowest time scale, the agent performs a word-recognition task. Given a word on the display, the agent must decide where to fixate within the word to acquire visual evidence. A fixation does not reveal the entire word: instead, it provides a limited, spatially localized observation determined by the foveal span. Specifically, when the agent fixates a position within the word, it observes a window of letters centred on that location (with a fixed foveal width), whereas letters outside this region remain uncertain. These observed letters are encoded and used to update the agent’s belief over candidate word identities, reducing lexical uncertainty as more informative letters are sampled. This means that when the agent is uncertain about a word, it tends to make additional fixations or choose more informative locations, whereas for highly predictable or easily identified words, it may terminate early with fewer fixations. Moreover, different fixation locations provide different diagnostic information. For example, fixating near the beginning of a word may reveal prefix information that distinguishes among candidates sharing similar suffixes. Thus, word recognition is not a passive process but an active information sampling problem: the agent must choose fixation locations that are informative for reducing uncertainty about the word’s identity under constraints on time and oculomotor effort.

We formalize this process as a resource-rational control problem. At each step, the agent maintains a belief distribution over candidate words and evaluates potential fixation locations in terms of their expected information gain, defined as the expected reduction in lexical entropy after observing letters at that location. Fixation actions are selected to maximize expected utility, which trades off uncertainty reduction against oculomotor and temporal costs. Through successive fixations, the agent integrates noisy observations to progressively reduce uncertainty until it decides to terminate and recognize the word.

- State *S*. *s* = (*x*, *I*, len, w^*^), where *x* is current fixation letter index; *I* is the letter information sampled so far. len is the word length; w^*^ is the ground-truth word identity. w^*^ is the ground-truth word (not directly observable to the agent).
- Action *A*. *a* = (*r*, *β*), where *r* denotes a coarse fixation region within the word (beginning, mid-left, mid-right or ending) and *β* ∈ {continue, stop}. When *β* = continue, the agent first selects a target location within the chosen region, and then, the actual landed fixation is sampled around this target owing to oculomotor noise. The final fixation position $x{\prime}$ therefore reflects both the agent’s decision and stochastic oculomotor variability, resulting in a distribution over landing positions rather than a deterministic choice. When *β* = stop, the agent terminates sampling and selects the most probable word under its current belief.
- Transition *T*. The environment is static; only the belief *b*(*w*) = *P*(*w*∣*I*) is updated. Beliefs follow ${b}^{{\prime} }(w)\propto P({I}^{^{\prime} }| w)b(w)$. The initial prior *P*(*w*) was proportional to corpus frequency (SUBTLEX-US^61^) or contextual predictability (GPT-2^62^). The likelihood *P*(*I*∣*w*) is computed with an ideal observer model (Supplementary Information section 1.4.1). To maintain a bounded lexical memory, only the top five candidates are retained after each update.
- Observation *O*. $o=({x}^{^{\prime} },{I}_{{x}^{^{\prime} }},\mathrm{len},\mathbf{b}^{\prime})$, where ${x}^{{\prime} }$ is the new fixation position, ${i}_{{x}^{{\prime} }}$ are letters revealed at ${x}^{{\prime} }$ and $\mathbf{b}^{\prime}$ is the updated belief.
- Reward *r*. *U* + *c*(*t*), where *U* is the final-step reward of correct (+100) or incorrect recognition (−100) and *c*(*t*) is the stepwise reward (−0.1), denotes the costs of eye movements.

Unlike heuristic word-recognition models that map lexical features directly to fixation durations, landing positions or stopping rules, our formulation frames word recognition as a control problem of uncertainty reduction under cost. Fixation timing, locations and termination decisions are derived from this objective rather than specified a priori. The resulting control policy is learned via reinforcement learning, rather than specified analytically, enabling fixation strategies to emerge from interaction with the word recognition task under resource constraints.

##### Fixation and gaze durations

The simulator produces fixation positions, which we convert into durations to enable comparison with human gaze data. Fixation time is modelled as a function of the lexical information gained during the fixation: greater reductions in lexical entropy Δ*H* correspond to longer processing times, modulated by a tunable parameter *κ*(refs. ^43,63^) and base fixation duration *d*_0_^6^. The mean lexical duration is

$${\mu}_{\mathrm{lex}}={d}_{0}+\kappa \,{\Delta }H,$$

clipped to the empirically observed range for single fixations of 200–250 ms (ref. ^6^). Observed durations are drawn from a Gamma distribution to reproduce the characteristic right-skew of human fixation times^64^. First-pass gaze duration for a word is computed as the sum of its first-pass fixation durations^6^. Saccades are assigned a fixed duration of 25 ms, following standard reading models^8^ and are excluded from gaze duration but included in total reading time under time-pressure evaluations. To account for non-fixation intervals (for example, blinks and tracking losses), we inflate each fixation duration by a constant overhead factor *ρ*^65^ estimated from human data. Total time for a word is therefore

$${D}_{\mathrm{word}}=\sum _{t\in \mathrm{first}\,{{-}}\,\mathrm{pass}}\left({d}_{\mathrm{lex}}^{\,t}(1+\rho )+{d}_{\mathrm{saccade}}\right).$$

Full derivations and details are provided in Supplementary Information section 1.4.1.

#### Sentence reader

At the medium time scale, the agent performs a sentence-reading control task. Given a sentence on the display, the agent selects eye-movement actions (fixate, skip or regress) to integrate word meanings in short-term memory and form a coherent sentence interpretation^66,67^. Sentence reading is formulated as a resource-rational control problem in which eye-movement policies are chosen to maximize expected sentence-level comprehension while minimizing time and oculomotor costs. The task is formalized as a POMDP in which the agent maintains and updates beliefs over both the current comprehension state and the identity of upcoming words under partial observability. Intuitively, sentence-level control determines how the reader moves through the text. When the current sentence is easy to integrate, or when upcoming words are predictable from context, the agent tends to move forward or skip words to save time. When uncertainty or integration difficulty increases, it becomes more likely to regress to earlier words to recover meaning before continuing.

- State *S*. *s* = (*j*, **c**, *ψ*_1:*j*_, *b*_next_), where *j* is the index of the currently fixated word, **c** is the short-term context buffer, *ψ*_1:*j*_ are appraisals of the words read so far (capturing their contribution to sentence-level integration) and *b*_next_ is the belief over the identity of the next word. The belief *b*_next_ summarizes uncertainty about upcoming input and corresponds to the prediction belief described in the main text.
- Action *A*. *a* = (*m*, *β*) with *m* ∈ {regress, next, skip} and *β* ∈ {continue, stop}. At each step, the agent selects the action that maximizes expected cumulative reward, balancing anticipated comprehension gains against time and eye-movement costs. Although the model can generate both interword and intraword regressions, where the latter arise from uncertainty during word recognition, our sentence-level evaluation focuses exclusively on between-word regressions.
- Transition *T*. Eye-movement transitions are deterministic. The belief over the next word is updated by combining parafoveal visual information with contextual predictions
  $${b}_{{\rm{next}}}^{{\prime} }(w)\propto P(w| \,\mathrm{letters}\,)\,{P}_{{\rm{LM}}}(w|c),$$
  followed by normalization. Here, *P*(*w*∣letters) encodes noisy parafoveal letter information (modulated by visual acuity and word length), whereas *P*_LM_(*w*∣*c*) captures contextual predictability from prior words using a pretrained language model (LM). Together, these terms incorporate lexical factors such as frequency, predictability and visual preview into the agent’s belief state.
- Observation *O*. $o=(j,\,{b}_{{\rm{next}}}^{{\prime} },\,{\psi }_{j},\,{u}_{t})$, where *ψ*_*j*_ is the appraisal of the currently fixated word and *u*_*t*_ is the running comprehension score. The comprehension signal *u*_*t*_ reflects how well the sentence has been integrated so far and corresponds to the comprehension belief described in the main text.
- Reward *r*. Each eye-movement step incurs an oculomotor cost of −0.1, scaled by a regression penalty *w*_reg_ for regress actions. When the agent chooses stop, a terminal reward is issued: a positive reward proportional to the final sentence-level comprehension score if the sentence has been fully read and a large negative reward otherwise. Full details are provided in Supplementary Information section 1.4.2.

Importantly, skipping and regression decisions are not triggered by lexical thresholds or structural rules. Instead, lexical information enters the model through the observation process and shapes the agent’s belief state, which is then used by the agent to evaluate the expected utility of alternative actions. As a result, eye-movement behaviour such as skipping predictable words or regressing under integration difficulty emerges from belief-guided control aimed at maximizing comprehension under resource constraints.

#### Text reader

At the text level, the agent maintains a capacity-limited, noisy representation of text meaning from the imperfect memory rather than a veridical record of all previously read content. The agent performs a text-reading control task. Given a multisentence passage, the agent selects which sentence to read or revisit to construct a coherent representation of the text. Text reading is formulated as a resource-rational control problem in which sentence-selection policies are chosen to maximize expected text-level comprehension while minimizing time, memory and rereading costs. As the agent does not have direct access to the true underlying text state and observes only its own memory appraisals of previously read sentences, the task is formalized as a POMDP. At each step, the agent selects a sentence to read or revisit to improve its belief about the evolving text representation. At the text level, the agent decides which parts of the text to revisit to build a coherent overall understanding. As memory is limited and noisy, the agent may be uncertain about how well earlier sentences have been integrated into the evolving representation of the text. For example, if earlier sentences were poorly understood or weakly integrated, the agent may choose to reread them to improve its memory representation and restore coherence. By contrast, when the text is well understood and uncertainty is low, it proceeds forward without revisiting earlier content. Thus, rereading behaviour reflects the agent’s attempt to reduce uncertainty about the overall meaning of the text under memory constraints.

- State *S*. *s* = (*k*, doc, *b*_text_), where *k* is the index of the current sentence, doc is the static document representation and *b*_text_ is the belief distribution over sentence-level appraisals accumulated so far.
- Action *A*. *a* = (*m*, *β*), where *m* ∈ {next, regress} and *β* ∈ {continue, stop}. For regress, the agent specifies the sentence index to revisit.
- Transition *T*. The text is static and eye movements land deterministically on the target sentence. After reading a sentence *j*, its appraisal *φ*_*j*_ is updated using a coherence score derived from a pretrained LM, normalized to [0, 1]. Details of the appraisal computation, integration mechanism and normalization appear in Supplementary Information section 1.4.3.
- Observation *O*. $o=(k,\,\mathbf{b}^{\prime}_{{\rm{text}}},\,{\varphi }_{k},\,{u}_{t})$, where $\mathbf{b}^{\prime}_{{\rm{text}}}$ is the updated belief over sentence appraisals, ${\varphi }_{k}$ is the appraisal of the currently visited sentence and ${u}_{t}$ is the running comprehension score.
- Reward *r*. Each reading step incurs a small oculomotor cost. When the agent chooses STOP, a terminal reward is issued: a positive comprehension-based score if the entire text has been read and a large negative penalty otherwise. The terminal score depends on the sentence-level appraisals accumulated during reading, with lower-appraisal sentences exerting disproportionately greater influence; full mathematical definitions and derivations are provided in Supplementary Information section 1.4.3.

##### Memory system

At the text level, the agent maintains a coarse representation of text meaning, which is updated after each sentence. The sentence is first mapped to a set of conceptual units, and a small subset is selected as gist elements based on their relevance and coherence with the evolving context. These selected elements update the agent’s long-term memory representation of the text, with repeated encounters strengthening their activation. Full details of the parsing procedure, schema-based selection mechanism and long-term memory update rules are provided in Supplementary Information section 1.4.3.

#### Reading under time pressure

To model the speed-accuracy trade-off imposed by time constraints, we retain the same hierarchical POMDP structure and introduce a small set of modifications at the sentence and text levels. These modifications control how time pressure alters the agent’s available steps, state representation and reward structure.

At the sentence level, the agent receives a time-derived budget of reading steps as state and observation signals, indicating the remaining allocation and whether the agent has exceeded this budget. Once the budget is exceeded, reading continues but incurs a small overtime penalty at each step. Skipping a word leads to a controlled degradation of its integration value, reflecting the information loss observed in human skipping behaviour. Early termination before all words are processed yields an additional penalty; otherwise, the usual sentence-comprehension reward is used. At the text level, both the state and observation are augmented with the global remaining time. Episodes terminate automatically when the available time expires or the agent issues the stop action. The terminal reward combines the text-level comprehension score with a progress-dependent bonus that encourages the agent to balance depth of processing with coverage under different time constraints. This leads to behaviour in which, under tighter time constraints, the agent reads more quickly by skipping more and regressing less, at the cost of reduced comprehension, reproducing the speed-accuracy trade-off observed in human reading. This pattern arises because the agent prioritizes actions that maximize expected comprehension under limited time, favouring faster but less thorough information sampling when resources are constrained. Implementation details are shown in Supplementary Information section 1.4.4.

### Training and simulation

#### Training

Each controller—the word recognizer, sentence reader and text reader—was trained independently in a reinforcement learning environment. Policies were parameterized by neural networks and optimized through trial-and-error interaction with their respective POMDP environments implemented in GYMNASIUM^68^ and trained using STABLE-BASELINES3^69^. This hierarchical training scheme avoids sparse-reward pathologies inherent in flat reinforcement learning by assigning each level a local objective with immediate feedback. Training stimuli consisted of synthetic or corpus-derived text materials. Critically, no human behavioural data (for example, gaze patterns or comprehension scores) were ever used during learning; all human data were reserved exclusively for post hoc evaluation.

#### Simulation and validation

After training, each controller was combined hierarchically to generate full reading simulations. Human behavioural datasets were used only for evaluation, enabling direct comparison between the model’s simulated eye-movement patterns and comprehension outcomes and those observed in readers. Validation followed the structure of the hierarchy: word-level simulations were assessed using gaze-duration effects, sentence-level simulations were evaluated through skip and regression behaviour and text-level simulations were assessed via recall and comprehension scores. Under time-pressure conditions, model predictions for both gaze dynamics and comprehension accuracy were compared against human adaptations to varying deadlines. Details of training and simulation can be found in Supplementary Information section 1.5.

### Parameters and fitting

Most model parameters *ϕ* were either anchored to established empirical findings or treated as tunable values optimized against behavioural benchmarks. Parameter definitions, literature justifications, ranges and final values are provided in Supplementary Information section 1.6 and summarized in Extended Data Table 1.

Model parameters *ϕ* were optimized to minimize a composite discrepancy between simulated and human data using a simulation-based, likelihood-free fitting procedure^1^ (see Supplementary Information section 1.6 for details). The discrepancy combined curve-level losses for conditional bin means (variance-aware reduced *χ*^2^), cell-mean losses when only few conditions were available (sum of squared errors) and distributional losses when full conditional distributions were available (Jensen–Shannon divergence^70^). Each metric subset was associated with its corresponding parameter(s): gaze-duration curves for *κ*, skipping/regression patterns for *w*_reg_, recall distributions for *τ*_H_ and *τ*_L_ and time-pressure adaptation metrics for *ρ*, *w*_TP_, *w*_IL_ and *w*_RP_. Fitting proceeded in stages: single-parameter subsets (*κ*, *w*_reg_, *τ*_H/L_) were tuned via exhaustive grid search over specified ranges, whereas the multiparameter time-pressure subset (*ρ*, *w*_TP_, *w*_IL_, *w*_RP_) was optimized using Bayesian optimization^71,72^ to efficiently explore the joint space with limited simulation-evaluation cycles. The staged procedure produced a final parameter set ϕ^*^ minimizing the composite objective and aligning simulated behaviour with human benchmarks; ϕ^*^ was shared across all analyses. All fitted parameters are theoretically grounded and optimized against aggregated human metrics, representing average rather than individual reading behaviour. Details of discrepancy function and tuning procedures can be found in Supplementary Information section 1.6.3.

### Reporting summary

Further information on research design is available in the Nature Portfolio Reporting Summary linked to this article.

## Supplementary information

Supplementary Information Supplementary Methods.

Reporting summary

Peer Review File

## Data Availability

All data supporting the findings of this study are available via the Open Science Framework (OSF) under accession https://osf.io/q2dm6/ at https://doi.org/10.17605/OSF.IO/Q2DM6. These include the newly collected eye-movement and comprehension data from the reading-under-time-pressure study, simulation data generated by the model and the processed datasets used for statistical analyses and figure plotting.

## Extended data

**Extended Data Table 1:** Assumptions and tunable parameters in the reading model

Type	Name	Explanation	Range	Value	Method
Visual perception	n_fov_	Number of high-acuity letters visible in foveal vision	[7, 9] letters	8 letters	L
Visual perception	n_parafov_	Number of previewable letters in the next word (parafoveal vision)	≤ 3 letters	2 letters	L
Eye movement	d_saccade_	Duration for executing a saccade	[20, 50] ms	25 ms	L
Eye movement	K	Processing time cost per bit of lexical information gain (entropy change)	(2, 4) ms/bit	3.50 ms/bit	O
Eye movement	ρ	Non-fixation overhead fraction	[0.1, 0.3]	0.30	O
Memory	C_STM_	Capacity of the short-term memory buffer	[5, 9]	5	L
Memory	τ_LTM_	Reader’s proposition activation threshold for integrating into the LTM	[0, 1]	0.32	O
Memory	τ_H_	High prior knowledge readers’ proposition activation threshold for merging into LTM	[0, 1]	0.838	O
Memory	τ_L_	Low prior knowledge readers’ activation threshold	[0, 1]	0.844	O
Information loss	w_IL_	Penalty weight for information loss due to skipped words	[0, 1]	0.70	O
Preferences	w_reg_	Weighting the cost of regressions	[0, 1]	0.80	O
Preferences	w_RP_	Weighting for forward reading progress over regressions	[0, 3]	1.50	O

L stands for Literature, O stands for Optimization.

## Code availability

The code used to implement the model, run the simulations and reproduce the analyses is available via GitHub at https://github.com/BaiYunpeng1949/hierarchical-resource-rational-reader. The repository also contains the analysis scripts and outputs used to generate the reported results and figures. The code is released under an open-source license.

## References

[1] Lieder, F. & Griffiths, T. L. Resource-rational analysis: Understanding human cognition as the optimal use of limited computational resources. *Behav. Brain Sci.* **43**, e1 (2020). 10.1017/S0140525X1900061X 30714890

[2] Simon, H. A. A behavioral model of rational choice. *Q. J. Econ.* **69**, 99–118 (1955).

[3] Simon, H. A. Rational choice and the structure of the environment. *Psychol. Rev.* **63**, 129–138 (1956). 10.1037/h0042769 13310708

[4] Anderson, J. R. *The Adaptive Character of Thought* (Psychology Press, 2013).

[5] Kahneman, D. *Thinking, Fast and Slow* (Macmillan, 2011).

[6] Rayner, K. Eye movements in reading and information processing: 20 years of research. *Psychol. Bull.* **124**, 372 (1998). 10.1037/0033-2909.124.3.372 9849112

[7] Kintsch, W. & Van Dijk, T. A. Toward a model of text comprehension and production. *Psychol. Rev.* **85**, 363 (1978).

[8] Reichle, E. D., Rayner, K. & Pollatsek, A. The E-Z Reader model of eye-movement control in reading: comparisons to other models. *Behav. Brain Sci.* **26**, 445–476 (2003). 10.1017/s0140525x03000104 15067951

[9] Salvucci, D. D. An integrated model of eye movements and visual encoding. *Cogn. Syst. Res.* **1**, 201–220 (2001).

[10] Legge, G. E., Klitz, T. S. & Tjan, B. S. Mr. chips: an ideal-observer model of reading. *Psychol. Rev.* **104**, 524 (1997). 10.1037/0033-295x.104.3.524 9243963

[11] Norris, D. The Bayesian reader: explaining word recognition as an optimal Bayesian decision process. *Psychol. Rev.* **113**, 327 (2006). 10.1037/0033-295X.113.2.327 16637764

[12] Bolliger, L. et al. ScanDL: a diffusion model for generating synthetic scanpaths on texts. In *Proc. 2023 Conference on Empirical Methods in Natural Language Processing* 15513–15538 (ACL, 2023).

[13] Bolliger, L. S., Reich, D. R. & Jäger, L. A. Scandl 2.0: a generative model of eye movements in reading synthesizing scanpaths and fixation durations. *Proc. ACM Hum. Comput. Interact.* **9**, 1–29 (2025).

[14] Van den Broek, P., Young, M., Tzeng, Y. & Linderholm, T. The landscape model of reading: inferences and the online construction of a memory representation. In *The Construction of Mental Representations During Reading* (eds van Oostendorp, H. & Goldman, S. R.) 71–98 (Lawrence Erlbaum Associates, 1999).

[15] Tapiero, I. *Situation Models and Levels of Coherence: Toward a Definition of Comprehension* (Routledge, 2007).

[16] Yeari, M. & van den Broek, P. A computational modeling of semantic knowledge in reading comprehension: integrating the landscape model with latent semantic analysis. *Behav. Res. Methods* **48**, 880–896 (2016). 10.3758/s13428-016-0749-6 27383752

[17] Reichle, E. D. *Computational Models of Reading: a Handbook* (Oxford Univ. Press, 2021).

[18] Gershman, S. J., Horvitz, E. J. & Tenenbaum, J. B. Computational rationality: a converging paradigm for intelligence in brains, minds, and machines. *Science* **349**, 273–278 (2015). 10.1126/science.aac6076 26185246

[19] Howes, A., Lewis, R. L. & Vera, A. Rational adaptation under task and processing constraints: implications for testing theories of cognition and action. *Psychol. Rev.* **116**, 717 (2009). 10.1037/a0017187 19839682

[20] Simony, E. et al. Dynamic reconfiguration of the default mode network during narrative comprehension. *Nat. Commun.* **7**, 12141 (2016). 10.1038/ncomms12141 PMC4960303 27424918

[21] Wang, L., Frisson, S., Pan, Y. & Jensen, O. Fast hierarchical processing of orthographic and semantic parafoveal information during natural reading. *Nat. Commun.* **16**, 8893 (2025). 10.1038/s41467-025-63916-y PMC12504734 41057333

[22] Pallier, C., Devauchelle, A.-D. & Dehaene, S. Cortical representation of the constituent structure of sentences. *Proc. Natl Acad. Sci. USA* **108**, 2522–2527 (2011). 10.1073/pnas.1018711108 PMC3038732 21224415

[23] Bacon, P.-L., Harb, J. & Precup, D. The option-critic architecture. In *Proc. 31st AAAI Conference on Artificial Intelligence* 1726–1734 (AAAI, 2017).

[24] Kliegl, R., Grabner, E., Rolfs, M. & Engbert, R. Length, frequency, and predictability effects of words on eye movements in reading. *Eur. J. Cogn. Psychol.* **16**, 262–284 (2004).

[25] Rayner, K., Slattery, T. J., Drieghe, D. & Liversedge, S. P. Eye movements and word skipping during reading: effects of word length and predictability. *J. Exp. Psychol.Hum. Percept. Perform.* **37**, 514 (2011). 10.1037/a0020990 PMC3543826 21463086

[26] Staub, A. The effect of lexical predictability on eye movements in reading: critical review and theoretical interpretation. *Lang. Linguist. Compass* **9**, 311–327 (2015).

[27] Rayner, K. Eye guidance in reading: fixation locations within words. *Perception* **8**, 21–30 (1979). 10.1068/p080021 432075

[28] McConkie, G. W., Kerr, P. W., Reddix, M. D. & Zola, D. Eye movement control during reading: I. The location of initial eye fixations on words. *Vis. Res.* **28**, 1107–1118 (1988). 10.1016/0042-6989(88)90137-x 3257013

[29] McNamara, D. S., Kintsch, E., Songer, N. B. & Kintsch, W. Are good texts always better? Interactions of text coherence, background knowledge, and levels of understanding in learning from text. *Cogn. Instr.* **14**, 1–43 (1996).

[30] McNamara, D. S. Reading both high-coherence and low-coherence texts: effects of text sequence and prior knowledge. *Can. J. Exp. Psychol.* **55**, 51 (2001). 10.1037/h0087352 11301728

[31] Freebody, P. & Anderson, R. C. Effects of vocabulary difficulty, text cohesion, and schema availability on reading comprehension. *Read. Res. Q.* **18**, 277–294 (1983).

[32] Vasishth, S., von der Malsburg, T. & Engelmann, F. What eye movements can tell us about sentence comprehension. *WIREs Cogn. Sci.* **4**, 125–134 (2013). 10.1002/wcs.1209 26304190

[33] Staub, A. & Clifton Jr, C. Syntactic prediction in language comprehension: evidence from either… or. *J. Exp. Psychol. Learn. Mem. Cogn.* **32**, 425 (2006). 10.1037/0278-7393.32.2.425 PMC1479855 16569157

[34] Duggan, G. B. & Payne, S. J. Skim reading by satisficing: evidence from eye tracking. In *Proc. SIGCHI Conference on Human Factors in Computing Systems* 1141–1150 (ACM, 2011).

[35] Vibert, N., Colas, Z. & Danion, F. R. The impact of reading time constraints on text comprehension and eye movements. *Read. Writ.* **39**, 1211–1233 (2026).

[36] Sutton, R. S. et al. *Reinforcement Learning: an Introduction* Vol. 1 (MIT Press, 1998).

[37] Schulman, J., Wolski, F., Dhariwal, P., Radford, A. & Klimov, O. Proximal policy optimization algorithms. Preprint at 10.48550/arXiv.1707.06347 (2017).

[38] Staub, A. & Rayner, K. Eye movements and on-line comprehension processes. In *The Oxford Handbook of Psycholinguistics* (ed. Gaskell, M. G.) 327–342 (Oxford Univ. Press, 2007).

[39] Vitu, F., McConkie, G. W. & Zola, D. About regressive saccades in reading and their relation to word identification. In *Eye Guidance in Reading and Scene Perception* (ed. Underwood, G.) 101–124 (Elsevier, 1998).

[40] Drieghe, D., Brysbaert, M., Desmet, T. & De Baecke, C. Word skipping in reading: on the interplay of linguistic and visual factors. *Eur. J. Cogn. Psychol.* **16**, 79–103 (2004).

[41] Blanchard, H. E., Pollatsek, A. & Rayner, K. The acquisition of parafoveal word information in reading. *Atten. Percept. Psychophys.* **46**, 85–94 (1989). 10.3758/bf03208078 2755766

[42] Levy, R. Expectation-based syntactic comprehension. *Cognition* **106**, 1126–1177 (2008). 10.1016/j.cognition.2007.05.006 17662975

[43] Smith, N. J. & Levy, R. The effect of word predictability on reading time is logarithmic. *Cognition* **128**, 302–319 (2013). 10.1016/j.cognition.2013.02.013 PMC3709001 23747651

[44] Hollenstein, N. et al. Zuco, a simultaneous eeg and eye-tracking resource for natural sentence reading. *Sci. Data* **5**, 1–13 (2018). 10.1038/sdata.2018.291 PMC6289117 30531985

[45] Hale, J. A probabilistic Earley parser as a psycholinguistic model. In *Proc. 2nd Meeting of the North American Chapter of the Association for Computational Linguistics* 159–166 (ACL, 2001).

[46] Bicknell, K. & Levy, R. A rational model of eye movement control in reading. In *Proc. 48th Annual Meeting of the Association for Computational Linguistics* 1168–1178 (ACL, 2010).

[47] Duan, Y. & Bicknell, K. A rational model of word skipping in reading: ideal integration of visual and linguistic information. *Top. Cogn. Sci.* **12**, 387–401 (2020). 10.1111/tops.12485 31823454

[48] Dayan, P. & Daw, N. D. Decision theory, reinforcement learning, and the brain. *Cogn. Affect. Behav. Neurosci.* **8**, 429–453 (2008). 10.3758/CABN.8.4.429 19033240

[49] Bai, Y. et al. Heads-up multitasker: simulating attention switching on optical head-mounted displays. In *Proc. 2024 CHI Conference on Human Factors in Computing Systems* Article 79, 1–18 (ACM, 2024).

[50] Shi, D. et al. CRTypist: simulating touchscreen typing behavior via computational rationality. In *Proc. 2024 CHI Conference on Human Factors in Computing Systems* Article 514, 1–17 (ACM, 2024).

[51] Shi, D., Wang, Y., Bai, Y., Bulling, A. & Oulasvirta, A. Chartist: task-driven eye movement control for chart reading. In *Proc. 2025 CHI Conference on Human Factors in Computing Systems* Article 1167, 1–14 (ACM, 2025).

[52] Lingler, A., Talypova, D., Jokinen, J. P. P., Oulasvirta, A. & Wintersberger, P. Supporting task switching with reinforcement learning. In *Proc. 2024 CHI Conference on Human Factors in Computing Systems* Article 82, 1–18 (ACM, 2024).

[53] Oulasvirta, A., Jokinen, J. P. P. & Howes, A. Computational rationality as a theory of interaction. In *Proc. 2022 CHI Conference on Human Factors in Computing Systems* Article 359, 1–14 (ACM, 2022).

[54] Botvinick, M. M. Hierarchical models of behavior and prefrontal function. *Trends Cogn. Sci.* **12**, 201–208 (2008). 10.1016/j.tics.2008.02.009 PMC2957875 18420448

[55] Rayner, K. Eye movements and attention in reading, scene perception, and visual search. *Q. J. Exp. Psychol.* **62**, 1457–1506 (2009). 10.1080/17470210902816461 19449261

[56] De Luca, M., Borrelli, M., Judica, A., Spinelli, D. & Zoccolotti, P. Reading words and pseudowords: an eye movement study of developmental dyslexia. *Brain Lang.* **80**, 617–626 (2002). 10.1006/brln.2001.2637 11896661

[57] Kangasrääsiö, A., Jokinen, J. P. P., Oulasvirta, A., Howes, A. & Kaski, S. Parameter inference for computational cognitive models with approximate Bayesian computation. *Cogn. Sci.* **43**, e12738 (2019). 10.1111/cogs.12738 PMC6593436 31204797

[58] Tobii pro spectrum. *Tobii Technology AB* https://www.tobii.com/products/eye-trackers/screen-based/tobii-pro-spectrum. (2019, accessed 29 October 2025).

[59] Tobii pro lab gaze filter. *Tobii Pro* https://connect.tobii.com/s/article/Gaze-Filter-functions-and-effects?language=en_US. (2024, accessed 29 October 2025).

[60] Al Madi, N., Torra, B., Li, Y. & Tariq, N. Combining automation and expertise: a semi-automated approach to correcting eye-tracking data in reading tasks. *Behav. Res. Methods* **57**, 72 (2025). 10.3758/s13428-025-02597-3 PMC11762023 39856463

[61] Brysbaert, M., New, B. & Keuleers, E. Adding part-of-speech information to the SUBTLEX-US word frequencies. *Behav. Res. Methods* **44**, 991–997 (2012). 10.3758/s13428-012-0190-4 22396136

[62] Radford, A. et al. Language models are unsupervised multitask learners. *OpenAI Blog* **1**, 9 (2019).

[63] Wilcox, E. G., Pimentel, T., Meister, C., Cotterell, R. & Levy, R. P. Testing the predictions of surprisal theory in 11 languages. *Trans. Assoc. Comput. Linguist.* **11**, 1451–1470 (2023).

[64] Staub, A., White, S. J., Drieghe, D., Hollway, E. C. & Rayner, K. Distributional effects of word frequency on eye fixation durations. *J. Exp. Psychol.Hum. Percept. Perform.* **36**, 1280 (2010). 10.1037/a0016896 PMC2948239 20873939

[65] Rayner, K. & Fischer, M. H. Mindless reading revisited: eye movements during reading and scanning are different. *Atten. Percept. Psychophys.* **58**, 734–747 (1996). 10.3758/bf03213106 8710452

[66] Lewis, R. L. & Vasishth, S. An activation-based model of sentence processing as skilled memory retrieval. *Cogn. Sci.* **29**, 375–419 (2005). 10.1207/s15516709cog0000_25 21702779

[67] Kuperberg, G. R. & Jaeger, T. F. What do we mean by prediction in language comprehension? *Lang. Cogn. Neurosci.* **31**, 32–59 (2016). 10.1080/23273798.2015.1102299 PMC4850025 27135040

[68] Towers, M. et al. Gymnasium: a standard interface for reinforcement learning environments. Preprint at 10.48550/arXiv.2407.17032 (2024).

[69] Raffin, A. et al. STABLE-BASELINES3: reliable reinforcement learning implementations. *J. Mach. Learn. Res.* **22**, 1–8 (2021).

[70] Lin, J. Divergence measures based on the Shannon entropy. *IEEE Trans. Inf. Theory* **37**, 145–151 (2002).

[71] Shahriari, B., Swersky, K., Wang, Z., Adams, R. P. & De Freitas, N. Taking the human out of the loop: a review of Bayesian optimization. *Proc. IEEE* **104**, 148–175 (2015).

[72] Snoek, J., Larochelle, H. & Adams, R. P. Practical Bayesian optimization of machine learning algorithms. In *Advances in Neural Information Processing Systems* (eds Pereira, F. et al.) Vol. 25, 2951–2959 (Curran Associates, 2012).

