# Supplementary material for: Hierarchical resource rationality explains human reading behaviour
Source: Nat Hum Behav. 2026 Aug 10;10(10):2029–45. doi: 10.1038/s41562-026-02534-0 (PMC13683046; doi:10.1038/s41562-026-02534-0)
Supplement: Supplementary file 1 [file 41562_2026_2534_MOESM1_ESM.pdf]

nature human behaviour

Supplementary information https://doi.org/10.1038/s41562-026-02534-0

Hierarchical resource rationality explains human reading behaviour

In the format provided by the authors and unedited

Contents

1 Supplementary Methods **3**

1.1 Datasets . . . 3

1.1.1 Kliegl et al. dataset: word recognition . . . 3

1.1.2 Rayner dataset: intraword regression location . . . 4

1.1.3 McConkie et al. dataset: initial fixation location . . . 4

1.1.4 ZuCo dataset: sentence reading . . . 5

1.1.5 McNamara et al. dataset: text reading . . . 6

1.1.6 Staub and Clifton dataset: regressions due to text ambiguity . . . 6

1.1.7 Our dataset: reading under time constraints . . . 7

1.1.8 Vibert et al. dataset: French reading under time constraints . . . 10

1.2 Validation metrics and simulation . . . 11

1.2.1 Word recognition . . . 11

1.2.2 Sentence reading . . . 12

1.2.3 Sentence ambiguity and its effects on eye movements . . . 12

1.2.4 Text comprehension . . . 13

1.2.5 Regression probability under syntactic ambiguity . . . 14

1.2.6 Reading under time pressure . . . 15

1.3 Computational principles . . . 16

1.3.1 Resource rationality . . . 16

1.3.2 Sequentiality . . . 16

1.3.3 Hierarchical Control . . . 17

1.4 Model Implementation . . . 19

1.4.1 Word recognizer . . . 19

1.4.2 Sentence reader . . . 22

1.4.3 Text reader . . . 24

1.4.4 Reading under time pressure . . . 26

1.5 Training . . . 28

1.5.1 Word recognizer 28
1.5.2 Sentence reader 29
1.5.3 Text reader 29
1.5.4 Models with time-pressure awareness 29
1.5.5 Implementation details 29
1.6 Parameters optimization 30
1.6.1 Parameters determined from literature 30
1.6.2 Parameters optimized 30
1.6.3 Discrepancy function 31
1.6.4 Metric subsets and tuned parameters. 33

1 Supplementary Methods

1.1 Datasets

1.1.1 Kliegl et al. dataset: word recognition

Human participants' word recognition data are obtained from the prior work[1]. In this study, sixty-five native German university students silently read 144 sentences from the Potsdam Sentence Corpus while their eye movements were monitored with an EyeLink system sampling at 250 Hz. After a standard nine-point calibration, each sentence was presented once, comprehension questions followed 25% of trials, and any trial with blinks, track losses, or fixations outside 80–120 ms was discarded. From the cleaned records the authors derived three first-pass word-based measures that index early word-recognition processes: first-fixation duration (FFD), single-fixation duration (SFD), and, most importantly for the present analysis, gaze duration (GD), which is the sum of all contiguous first-pass fixations on a word before the eyes move away. To predict GD they entered three continuous lexical variables:

- Word length: number of letters.
- Printed frequency: log_{10} of type frequency (tokens per million) taken from the German CELEX database.
- Contextual predictability: cloze probability p obtained from an independent norming study and converted to a bias-corrected logit, i.e., $log[p/(1-p)]$.

The experimental task (natural sentence reading), the eye-movement measures, and these lexical predictors were chosen precisely because they isolate word-recognition difficulty independent of later integration or spill-over effects.

Although the dataset was collected in German, it is well-suited for validating an English reading model: the core drivers of early word recognition (word length, lexical frequency, and contextual predictability) show virtually identical effects across alphabetic scripts. Evaluating our model on the Potsdam corpus therefore provides a rigorous, language-general check without needing to fine-tune on English data. Because the model is grounded in resource-rational principles that capture these shared mechanisms, it can run on German corpora without modification.

1.1.2 Rayner dataset: intraword regression location

Human data on within-word regression behavior are obtained from Rayner's eye-movement study[2]. In this experiment, eight skilled adult readers silently read approximately 1600 words of continuous text while their eye movements were recorded using a high-resolution eye-tracking system with spatial accuracy of about one character position. The analysis focused on fixation locations within words of varying lengths (3-10 letters). Eye movements were classified into forward fixations and regressive fixations, with regressions further divided into interword and intraword regressions. For each word length, the authors computed histograms indicating the percentage of time that fixations landed on each letter position. Concretely, fixation proportion at a given letter position was defined as:

$$\text{Fixation proportion at position } i = \frac{\text{number of fixations landing on position } i}{\text{total number of fixations of that type for the word length}}.$$

For intraword regressions, the data (Fig. 3 in the original paper) show a clear spatial bias: when regressions occur within a word, they are most likely to land near the beginning of the word, with fixation probability decreasing toward later letter positions. This pattern reflects the tendency to revisit earlier, more informative parts of the word during ongoing lexical processing.

In the present work, we extract these normalized fixation distributions over letter positions for intraword regressions. These distributions provide a direct benchmark for evaluating whether the model reproduces the spatial structure of corrective eye movements during word recognition.

1.1.3 McConkie et al. dataset: initial fixation location

Human data on initial fixation locations are obtained from McConkie et al.[3], which examined where readers' eyes land when first fixating a word during natural reading. Participants read continuous text while their eye movements were recorded with high spatial resolution, allowing fixation locations to be mapped onto individual letter positions. The analysis focused on first-fixation landing positions as a function of word length. For each word length, the authors computed the distribution of landing positions across letter indices, expressed as the proportion of first-pass fixations that landed on each letter position. Specifically,

for a given word length, fixation proportion at position i was defined as:

$$\text{Fixation proportion at position } i = \frac{\text{number of first fixations landing on position } i}{\text{total number of first fixations on words of that length}}.$$

The results reveal a robust preferred viewing position (PVP): initial fixations tend to land slightly to the left of the word center, with variability increasing as word length increases. This pattern reflects both oculomotor variability and the strategy of targeting informative regions of the word.

In the present analysis, we reconstruct these first-fixation distributions over letter positions for each word length and normalize them to obtain comparable probability profiles. These data serve as a benchmark for evaluating whether the model reproduces human-like fixation targeting during the initial sampling of a word.

1.1.4 ZuCo dataset: sentence reading

Sentence-level reading behaviour was taken from the ZuCo 1.0 corpus, Task 2 *Normal reading*[4]. Twelve healthy, right-handed native English speakers (5 F, 7 M; 22–54 years, mean LexTALE = 94.7%) silently read 300 English sentences (6386 tokens; 5–62 words per sentence) that describe biographical facts about notable people. Each sentence appeared once, self-paced: readers pressed a button to move to the next sentence and to answer a multiple-choice comprehension question on 68/300 trials (mean accuracy = 88%), ensuring attentive reading.

Eye movements were recorded with an EyeLink 1000 Plus (SR Research) at 500 Hz in a sound-attenuated booth. Stimuli were presented on a 24-inch 100 Hz monitor (800 × 600 px, viewing distance = 68 cm); text was black 20-pt Arial on a light-grey background (letter height = 0.67°). A 9-point calibration preceded each block of 60 sentences, with drift correction whenever error $> 0.5°$ occurred. Fixations shorter than 100 ms were discarded. For every word the corpus supplies (i) first-fixation duration (FFD), (ii) single-fixation duration (SFD), (iii) gaze duration (GD), (iv) go-past time (GPT), (v) total reading time (TRT), plus fixation count and pupil size.

ZuCo offers tightly synchronised eye-tracking under naturalistic, self-paced conditions, providing rich per-sentence timing that aligns with our sentence-reading task. The vocabulary, sentence lengths, and comprehension checks match typical English NLP corpora, letting us evaluate the model's predictions at

the sentence level without additional calibration.

1.1.5 McNamara et al. dataset: text reading

Our comprehension benchmark comes from the *"Are Good Texts Always Better?"* study (Experiment 2) by McNamara et al.[5]. Fifty-six junior-high students (10–15 years, Grades 7–10) were randomly assigned (14 per cell) to one of four versions of an English encyclopedia passage on heart disease that factorially crossed local coherence (high vs. low) with global coherence (macrosignals present vs. absent). Before reading, a 41-point heart-knowledge quiz split readers into *high-* and *low-knowledge* groups (median split). Each participant read the passage twice, giving an oral free-recall after each reading; the two protocols were pooled and propositionalised.

The primary metric is proportional recall of the 70 propositions common to all versions (63 micro 7 macro). Figure 3 of the paper reports these recalls as a 2 (knowledge) $\times$ 2 (coherence) matrix for the most-coherent (CM) and least-coherent (cm) texts. Because only the CM and cm passages are reprinted verbatim in Appendix B, we restrict our simulation to these two text variants while retaining the knowledge split. The resulting four data points, including high-knowledge CM, low-knowledge CM, high-knowledge cm, and low-knowledge cm—constitute the ground truth against which we evaluate the model.

McNamara et al.'s materials are purpose-built to orthogonally manipulate text coherence (by adding or removing local and global cues) and reader knowledge (via an independent pre-test), the very variables our comprehension model claims to capture. Few corpora provide such clean experimental control at the passage level; using these passages therefore offers a benchmark for testing whether our model can predict how coherence and prior knowledge jointly shape what readers remember and comprehend.

1.1.6 Staub and Clifton dataset: regressions due to text ambiguity

Text-level regression behavior was evaluated using the syntactic ambiguity experiment reported by Staub and Clifton[6]. In this study, twenty-four native English speakers participated in an eye-tracking experiment designed to test whether readers make syntactic predictions during sentence comprehension. Participants read sentences containing temporarily ambiguous constructions involving the coordination structure "either...or". In these sentences, readers may initially adopt an incorrect syntactic interpretation that must be revised once the disambiguating region of the sentence is encountered.

Eye movements were recorded while participants read the sentences under normal reading conditions. The critical measure was the probability of initiating a regression from the disambiguating region of the sentence, which reflects the likelihood that readers revisit earlier material when syntactic reanalysis is required. Regression probability was calculated as the proportion of trials in which a regression was launched relative to the total number of trials in each condition.

The experiment compared sentences containing syntactic ambiguity with matched unambiguous controls. The results showed that readers initiated substantially more regressions when processing ambiguous sentences than when reading unambiguous sentences. For the human benchmark used in our evaluation, we extracted the summary statistics reported in Table 1 of the original study, which provide the mean regression probabilities for the ambiguous and unambiguous conditions (24 participants). These values serve as empirical reference points for evaluating whether the model reproduces the qualitative increase in regressions observed under syntactic ambiguity.

1.1.7 Our dataset: reading under time constraints

Participants We recruited 39 adult participants through university mailing lists and online announcements. After data-quality exclusions, 32 participants were retained for comprehension analyses and 28 participants were retained for eye-movement analyses. The 32 participants retained for comprehension analyses included 19 female and 13 male participants (mean age = 24.0 years, SD = 3.7, range = 18–34 years). Participants had normal or corrected-to-normal vision, reported no reading impairments, and met the English-proficiency criteria for the task. All participants provided informed consent before participation and received food coupons worth 50 RMB.

Experimental design The study used a within-subject design with one independent variable: text reading time constraints at 30, 60, and 90 seconds. These intervals were chosen based on a pilot study, which showed that participants typically needed around 70 seconds to read the materials. The selected times are intended to create varying levels of time pressure: 30 seconds (high pressure), 60 seconds (moderate pressure), and 90 seconds (low pressure). This design allows us to explore user behavior under different time-induced pressure conditions.

Task Participants were tasked with silently reading a series of texts presented on a monitor, with each text constituting a single trial conducted under a specific time constraint. During each trial, eye movement data was collected to analyze reading behavior. Following each reading trial, participants completed a 20-second arithmetic task. This was immediately followed by a comprehension test, which included a free-recall task and five multiple-choice questions (MCQs).

Material Our corpus consists of nine distinct stimuli, all generated by ChatGPT-4. We chose to use ChatGPT-4 to create the materials for two key reasons: first, to prevent participants from being influenced by any prior knowledge of existing reading materials, and second, to avoid potential bias in our model's comprehension ability, as current reading materials might have been used as training data for LLMs, including the one we employ for text comprehension. Each reading material contains an average of 139.44 words (SD = 4.03, range 134–146 words per material), aligning with typical lengths used in reading comprehension assessments[7]. The readability of the materials was controlled, with an average Flesch-Kincaid Grade Level of 10.38 (SD = 1.09, range 8.90-11.90), ensuring they are appropriate for English speakers with university-level proficiency. Extended Figure 9 illustrates the stimulus presentation setup. Consistent with the approach by Zermiani et al.[8], each reading material was presented on a single page, formatted in Courier, a black monospaced font at 16px (0.44 DVA) character height, with 2.5-line spacing, left-aligned at 400px, and vertically centered on a white background. The comprehension materials included five multiple-choice questions (MCQs) per reading material, with five options each. Four options were content-related, and one was labeled "cannot remember or do not know." These comprehension tests were also generated by ChatGPT-4. To ensure the validity and effectiveness of these comprehension tests, we conducted extensive sanity checks. We paid close attention to avoid any biases, such as answers being inferable from length, attitude, or other non-content-related cues. We ensured that correct answers could only be derived from careful reading of the corresponding text. Both co-authors and our model confirmed that without reading the text, these questions could not be answered correctly. The arithmetic tasks, involving basic operations such as addition, subtraction, multiplication, and division, were designed to match a primary school level of difficulty. These tasks were sufficient for clearing short-term memory[9]. All sanity check results, materials, and prompts used for generation are available in the supplementary materials.

Procedure First, participants were briefed on the study's objectives and procedures and provided informed consent to share anonymized data with the scientific community. They were then seated at a desk equipped with an adjustable head and chin rest, a monitor, and an eye tracker, while the investigator was seated at another desk behind. Participants were informed that a time reminder would be provided halfway through each trial. They were advised to read attentively, as they would be required to complete a brief comprehension test afterward. Prior to the reading task, we collected demographic data and assessed participants' background knowledge on the content of the text[8]. Participants began with a training trial to familiarize themselves with the task, equipment, and to adjust to a comfortable sitting position with stabilized head posture. The eye tracking recording started with a five-point calibration. The main experiment consisted of nine reading trials, with each trial involving a distinct text and one of three fixed time durations: 30 seconds, 60 seconds, or 90 seconds. The trials were grouped by time constraint, with three trials per duration. The order of these time constraints and corresponding reading materials was counterbalanced using a Latin-square design to control for order effects within each group. After each reading trial, participants completed a 20-second arithmetic task to clear short-term memory[9], followed by a free-recall test and five multiple-choice questions (MCQs) to assess reading comprehension[10]. The entire experiment lasted approximately 40 minutes.

Recording setup The data collection was conducted within a controlled laboratory environment with consistent lighting conditions. We used a Tobii Pro Spectrum screen-based eye tracker2 operating at a sampling frequency of 1200Hz. All stimuli were presented on the native Tobii Pro Spectrum screen (EIZO FlexScan EV2451) with dimensions of 52.8 × 29.7 cm and a resolution of 1920 × 1080 px. The screen was placed 60 cm in front of the participants, who positioned their eyes in the center of the Tobii Pro Spectrum headbox. Both the eye tracker and its monitor were connected to a desktop computer running the recording pipeline. The eye movement data were collected by Tobii Pro Spectrum automatically during the experiment. For the study, we directly used Tobii's built-in software, Tobii Pro Lab version 1.217.49450, to process fixation data using the Tobii I-VT (Fixation) filter to classify fixation and saccade events.

Data process Using the pre-processed eye movement data provided by Tobii I-VT, we directly extracted gaze features. For comprehension analysis, we evaluated participants' performance by scoring their free

recall responses and calculating their accuracy on multiple-choice questions.

Data correction Although the Tobii Pro Spectrum offers advanced and highly accurate eye-tracking capabilities, inherent dynamic eye movement patterns in humans can still cause offset issues in eye movement data. Further adjustments were necessary to better align fixations with the text lines. After data collection, we used Fix8 software[11], employing a combination of time warping and chain filters to correct eye movement data. This correction was applied to both human and simulated data, ensuring improved alignment with the text lines.

Data cleaning Data exclusions were applied according to pre-established data-quality criteria. Of the 39 participants initially recruited, 7 were excluded from comprehension analyses because of invalid or outlying comprehension/gaze measures. For eye-movement analyses, an additional 4 participants were excluded because of excessive eye-tracking data loss or calibration/recording-quality issues, leaving 28 participants with valid eye-movement data. Outliers were identified using the interquartile-range criterion, and full exclusion criteria are reported in the main Methods and accompanying materials.

1.1.8 Vibert et al. dataset: French reading under time constraints

To benchmark our model against established empirical patterns of eye-movement adaptation under time pressure, we drew on findings from the recent study by Vibert et al.[12], which examined how French readers' gaze and comprehension change when reading time is limited. In that within-participant experiment, thirty native French adults read short three-sentence texts ($\approx$ 65 words each) under five viewing durations (6.3 s, 9.5 s, 19.0 s, 38.0 s, and free reading). The study provided a detailed account of how core oculomotor variables, including mean fixation duration, saccade amplitude, saccade rate, gaze velocity, regression proportion, proportion of directly fixated content words, proportion of time devoted to fixations, and the sentence-final "wrap-up" effect varied systematically with reading time.

Rather than reusing Vibert et al.'s materials or time settings, or using their data as baselines for comparison, we analyzed our own English stimuli under corresponding 30 s, 60 s, and 90 s conditions using the same set of eye-movement metrics. This approach allowed us to test whether our model reproduced the *directions of effect* identified in the French dataset, such as shorter fixations, larger saccades, faster

gaze velocity, and fewer directly fixated content words as time pressure increased. The Vibert et al. study therefore served as an external reference for capturing the directional patterns of key metrics and validating the qualitative consistency of the model's adaptive behavior under time constraints.

1.2 Validation metrics and simulation

To assess whether the model reproduces human behavior across the three processing levels it is designed to span. We evaluated it using a complementary set of established eye-movement and comprehension metrics. Gaze duration pinpoints word-level recognition difficulty; skip and regression probabilities capture the effect of individual words' lexical features on eye movements at the sentence level reading; proportional recall tests whether the model reproduces the classic coherence × knowledge interaction in long-term memory. Under time pressure, speed, skipping, and regressions jointly characterize the strategic adjustments of the eye-movement system, while recall similarity and MCQ accuracy reveal the cost of those adjustments for comprehension. Each metric was selected because (1) it has a well-documented role in psycholinguistics, (2) it captures processing at the intended level of analysis, and (3) it can be computed identically for human and simulated data, enabling direct comparison.

1.2.1 Word recognition

At the lexical scale, we focus on gaze duration (GD) – the total time spent on a word during its first pass, from the moment the eyes first enter the word until they move past it. GD is a classic measure of lexical identification difficulty, sensitive to word length, frequency, and predictability effects in both human and computational studies[1]. Formally,

$$\mathrm{GD}_w = \sum_{f=1}^{F_w} t_f \tag{1}$$

where F_w are the first–pass fixations on word w. This measure allows us to test whether the model reproduces established lexical effects reported in the empirical study.

For model's word-level validation, we focused on reproducing effects rather than matching specific fixation sequences. The pretrained word recognizer was run on a synthetic corpus, generating detailed fixation logs specifying where and when the agent fixated on each letter. From these logs, we computed gaze durations and then followed exactly Kliegl's procedure[1] to derive word length, log frequency, and logit

predictability measures. This allowed us to directly plot model-predicted effects alongside those reported in human data. The only tuned parameter at this level was κ (see Parameter Optimization of Methods, main text).

1.2.2 Sentence reading

At the sentence scale, we quantify the skip probability: the proportion of trials in which a word is not fixated at all, and the regression probability: the proportion of trials in which the eyes move back into a word after leaving it. Both measures are sensitive to lexical accessibility and contextual integration difficulty[1]. Skipping indicates that the model deems a word dispensable for ongoing comprehension, while regressions signal the need to revisit earlier material to resolve integration failures. Together, they test whether the model captures the influence of word-level features on sentence-level eye movement control.

For model's sentence-level validation, we compared aggregated recall performance against the McNamara et al. dataset[5]. The text reader agent generated sentence-level regression and fixation decisions for synthetic text environments. An LLM-based parser analyzed read sentence propositions to assess coherence and relevance. Propositions with activation above the tunable threshold $\tau_{\text{H or L}}$ were stored as gists in long-term memory (LTM). Recall percentage was computed as the proportion of stored propositions relative to the total propositions in the text. Due to limited availability of open-source materials, evaluation was conducted on the single publicly released text from the McNamara study.

1.2.3 Sentence ambiguity and its effects on eye movements

To examine whether the model captures sentence-level integration effects, we analyzed how sentence ambiguity relates to eye-movement behaviour during reading. Sentence ambiguity was estimated using an uncertainty-based measure derived from a probabilistic language model. Specifically, for each sentence consisting of words $w_1, \ldots, w_n$, we computed the predictive distribution over the next word given the preceding context using a pretrained causal language model. At each position t, the model estimates the probability distribution

$$P(w_{t+1} \mid w_1, \ldots, w_t) \tag{2}$$

over possible next tokens. Ambiguity was operationalized as the entropy of this predictive distribution:

$$H_t = -\sum_i P(w_i \mid w_1, \ldots, w_t) \log P(w_i \mid w_1, \ldots, w_t) \tag{3}$$

where higher entropy indicates greater uncertainty about the upcoming continuation. Sentence-level ambiguity was defined as the mean predictive entropy across all incremental contexts within the sentence:

$$\text{Ambiguity}(s) = \frac{1}{n-1} \sum_{t=1}^{n-1} H(P(w_{t+1} \mid w_1, \ldots, w_t)) \,. \tag{4}$$

This measure captures the average uncertainty about sentence continuations and serves as a proxy for semantic ambiguity during incremental sentence processing.

To relate sentence ambiguity to eye-movement behaviour, we computed sentence-level skipping and regression probabilities from both human eye-tracking data and model simulations. Word-level skipping and regression probabilities were first obtained as described above: skipping probability for a word was defined as the proportion of trials in which the word received no first-pass fixation, whereas regression probability was defined as the proportion of trials in which gaze returned to the word after it had already been passed. For the sentence-level analysis, these word-level probabilities were aggregated within each sentence. Specifically, for a sentence containing n words, sentence-level regression and skipping probabilities were computed as the mean of the corresponding word-level probabilities across all words in the sentence. To facilitate comparison between human data and model simulations, sentences were partitioned into bins according to their ambiguity values using quantile-based binning. For each bin, we computed the mean ambiguity together with the mean regression and skipping probabilities across sentences in that bin. Linear regression analyses were then used to quantify the relationship between sentence ambiguity and eye-movement behaviour in both human data and model simulations.

1.2.4 Text comprehension

At the text-reading level, we measure proportional recall rate, following[5]. After reading, each participant's (or model's) recall is parsed into micro-structural propositions, and the score is the proportion of target

propositions from the original text successfully recalled:

$$\text{Recall} = \frac{\text{number of propositions recalled}}{\text{number of total propositions}} \tag{5}$$

This metric is sensitive to both text coherence and the reader's prior knowledge, which are two variables directly targeted by our comprehension module.

For model's text-level validation, simulation was conducted on the McNamara et al. dataset[5]. The agent's text reader produced regression and fixation decisions, and an LLM-based parser analyzed read sentence propositions for coherence and relevance. Propositions exceeding the activation threshold $\tau_{\text{H or L}}$ were stored as long-term memory gists. Recall percentage was then computed as the proportion of stored propositions relative to total text propositions.

1.2.5 Regression probability under syntactic ambiguity

To evaluate whether the model reproduces human rereading behaviour under syntactic ambiguity, we measured the probability of initiating regressions when processing ambiguous versus unambiguous sentences, following the experimental comparison reported by Staub and Clifton[6]. We used the same sentence stimuli as in the original study. Sentences were grouped according to the ambiguity manipulation defined in the experiment, which distinguishes sentences that temporarily induce syntactic misanalysis (ambiguous condition) from those in which the correct structure is predicted (unambiguous condition).

Each sentence was processed by the agent across multiple simulated reading episodes. During each episode, the agent sequentially processed the sentence and could choose among forward reading, regressing to previously read text, or terminating the reading episode according to its learned policy. Regression probability was computed as the proportion of simulated reading episodes in which the agent initiated at least one regression while processing the sentence:

$$\text{Regression Probability} = \frac{\text{number of episodes containing at least one regression}}{\text{total number of episodes}} \tag{6}$$

The resulting probabilities were averaged separately for the ambiguous and unambiguous conditions and compared with the empirical regression percentages reported in Table 1 of Staub and Clifton[6].

1.2.6 Reading under time pressure

When time is constrained, we analyze both eye-movement adjustments and comprehension outcomes. Eye movement adjustments are captured by reading speed (words per minute), skip rate (proportion of forward saccades that bypass at least one word), and regression rate (proportion of words that receive at least one regression). These measures have a long tradition in reading research and are also reported in datasets such as ZuCo[4], where omission rates and speed changes reflect strategic adaptation under task constraints.

Comprehension under time pressure was evaluated using two complementary metrics: free-recall similarity and multiple-choice accuracy (MCQ). For free recall, both the participant's recall protocol and the original passage were embedded in a transformer-based language model (Universal Sentence Encoder), and cosine similarity was computed to yield a continuous measure of semantic overlap. Embedding-based similarity has been increasingly recognized in memory research as a robust way to quantify recall fidelity, offering greater consistency and reproducibility than manual coding while capturing semantic content beyond surface word overlap[10]. In contrast, MCQ accuracy serves as a conventional, face-valid measure of passage understanding and facilitates direct comparison with human experimental performance.

For validation, we compared aggregated metrics against human data collected in our dedicated experiment (Sec Datasets of Methods in the main text), including reading speed, skipping and regression rates, MCQ accuracy, and free recall performance. The full hierarchical agent (word → sentence → text) was run as an integrated system on the same experimental texts shown to participants. The agent's LTM gists were used to answer comprehension questions and produce recall summaries, mirroring the human task protocol. Model outputs were aggregated into the same metrics as the human dataset for direct comparison. Tunable parameters at this stage were w_{TP}, w_{IL}, and w_{RP} (see Parameter Optimization of Methods in main text).

To further evaluate the qualitative consistency of the model's adaptive eye-movement control under time constraints, we examined five oculomotor metrics derived from Vibert et al.[12]. These include (i) *mean fixation duration* (average time spent per fixation), (ii) *saccade amplitude* (mean distance between consecutive fixations), (iii) *saccade rate* (number of saccades per second), (iv) *mean left-to-right gaze velocity* (forward eye-movement speed across the text), and (v) *proportion of directly fixated content words* (share of content words receiving at least one fixation). Unlike the original French-language experiment,

we analyzed our own English stimuli under three conditions (30, 60, and 90,s). Our goal was to test whether the model reproduces the *directions of effect* identified in their study.

1.3 Computational principles

1.3.1 Resource rationality

First we frame reading as a *Resource-Rational Control Problem*[13,14] where the reader agent learns a boundedly optimal policy π^* to maximize the expected utility of comprehension and information gain (U), while minimizing the effort cost (C), under constraints imposed by both cognitive (ρ) and temporal (t) resources[13]. Formally:

$$\pi^* = \arg\max_{\pi\in\Pi} \underbrace{\mathbb{E}[\, U(s,a) \,]}_{\text{Expected Utility}} - \underbrace{\mathbb{E}[\, C(s,a;\rho,t) \,]}_{\text{Expected Cost}} . \tag{7}$$

where s is the current reading state, a is an action (e.g., moving the eyes to a new location), $U(s,a)$ denotes the comprehension or information-gain utility of taking a in s, and $C(s,a;\rho,t)$ is the cognitive and temporal cost of that action under the available resources. Expectations are taken over the states and actions encountered when following π. In our implementation, s is only partially observable and thus represented by a belief state b held by agent's internal memory system.

1.3.2 Sequentiality

Secondly, reading is inherently sequential: information is sampled and accumulated fixation by fixation, and decisions of where to read next are made at each step[15]. Due to the limitations of human visual perception[15], the reader only partially observes the environment at any moment, leading to uncertainty about the underlying states of the text and its meaning. We capture this uncertainty by formulating reading as a *Partially Observable Markov Decision Process (POMDP)*[16], where the agent cannot directly access the true state of the environment s but must infer it from noisy observations o. The observation model $P(o' \mid s')$ specifies how partial visual input (letters sampled by fixations) arises from the reading word or sentence state, while the transition model $P(s' \mid s, a)$ defines how the text and memory states evolve after an eye movement a. The agent maintains a belief state b – a probability distribution over possible latent

states – that is updated after each action-observation cycle:

$$b'(s') \propto P(o'|s') \sum_{s} P(s'|s,a) b(s) \tag{8}$$

Here, $b'(s')$ summarizes the agent's updated best estimate of the text and memory state, integrating new sensory evidence with prior expectations. o' is the new observation after the action takes effect (e.g., new letters acquired during fixation). The state s may include both external variables (such as the identity and location of words in the text) and internal variables (such as the reader's memory contents). For example, in lexical processing, $b(s)$ could encode the probabilities of candidate words given the currently visible letters; in sentence processing, it might encode predictions about upcoming words based on context and parafoveal preview. As new evidence is acquired, the belief state $b'(s')$ is iteratively updated becomes the basis for selecting the next action, serving as a compact, computable summary of the reader's knowledge that guides subsequent eye-movement decisions.

1.3.3 Hierarchical Control

Eye-tracking and neural dynamics indicate that reading is coordinated by hierarchical control operating at different time scales[17–19]. For example, readers apply fixations around 200 – 250 ms for lexical decoding and oculomotor execution[15]. MRI then shows two slower steps: the brain needs about eight seconds to assemble the meaning of a single sentence and roughly half a minute to knit sentences into a coherent paragraph[20]. Thus, word recognition, sentence interpretation, and discourse-level integration unfold one after another, each on its own time scale. To model this multi-time-scale structure, we apply *Hierarchical Control* using reinforcement learning[21]. At each scale a controller operates over its own time scale and resource constraints, and can invoke the controller below it in a call–and–return fashion ("invoke and wait"). This yields three levels: 1. Word Recognizer: selects intra-word fixation targets to reduce lexical uncertainty subject to oculomotor cost. 2. Sentence Reader: chooses the next word to fixate, skip, or regress given reading progress, incremental comprehension state, and predictions about upcoming words so as to maximize sentence-level understanding. 3. Text Reader: selects which sentence to read (or re-read) next by evaluating global coherence and sentence-level memory appraisals in order to optimize text-level comprehension. This hierarchy is formalized through a level-indexed Q-value function shown

below, which estimates the expected future reward of taking an action in a given state and continuing to behave optimally thereafter, forming the basis for learning and decision-making[16].

$$Q_\ell(s_\ell, a_\ell) = \sum_{\tau, s'_\ell} \underbrace{F_\ell(\tau \mid s_\ell, a_\ell)}_{\text{Duration distribution}} \underbrace{P_\ell(s'_\ell \mid s_\ell, a_\ell, \tau)}_{\text{State transition}} \underbrace{\left[R_\ell(s_\ell, a_\ell) + \gamma_\ell^\tau V_\ell(s'_\ell)\right]}_{\text{Expected return}}, \tag{9}$$

where $\ell \in \{\mathrm{W}, \mathrm{S}, \mathrm{T}\}$ indexes the level (Word, Sentence, Text). This equation makes explicit that each controller solves its own resource-rational problem: an action a_ℓ produces a reward R_ℓ, occupies a variable duration τ governed by F_ℓ (the distribution function), leads to a new belief state s'_ℓ, and is evaluated in terms of both local objectives and the downstream value of future states. The inclusion of γ_ℓ^τ captures that longer-duration actions at higher levels (e.g., re-reading a paragraph) are discounted differently from rapid lexical fixations. This level-indexed Q-value function formalizes the optimization problem each controller faces at its own temporal and informational granularity. It also infers why a flat reinforcement learning (RL) would fail: if the model were trained end-to-end to map from raw fixations to final comprehension reward, the reward signal would be extremely sparse, requiring the agent to discover thousands of saccade-level decisions before receiving feedback. This "sparse-reward trap"[22] makes credit assignment intractable. Hierarchical decomposition overcomes this by breaking the reading task into temporally extended subtasks, each with its own reward function that delivers immediate feedback for intermediate goals (e.g., accurate word recognition, coherent sentence integration, or successful text comprehension), as detailed in the Methods section.

In our implementation we train the word, sentence, and text controllers separately under their local resource-rational objectives and compose them via call-and-return models, rather than jointly propagating value gradients across levels. This mirrors supervisory and option-based hierarchical RL treatments of temporally extended subtasks[23]. Each level encodes distinct cognitive operations that jointly reproduce realistic reading dynamics. Removing the Word Recognizer would eliminate the fundamental process of lexical identification, thereby degrading temporal fidelity and preventing the model from capturing the parafoveal preview and parallel activation that drive natural eye movement planning. Removing the Sentence Reader would reduce reading to a mechanical feed-forward process, losing adaptive trade-offs between skipping and regressions. Finally, excluding the Text Reader would prevent the model from

monitoring global coherence or initiating targeted rereading. Thus, all three controllers are necessary to reflect the hierarchical organization of human reading.

1.4 Model Implementation

1.4.1 Word recognizer

From action to fixation location At each time step, the agent selects a fixation action that determines where visual information is acquired within the word. The action space is defined over a set of coarse spatial regions: beginning, mid-left, mid-right, and ending, together with a termination action. This coarse-to-fine formulation reflects the limited spatial precision of saccadic planning and reduces the dimensionality of the control problem.

Fixation selection proceeds in three stages. First, the agent selects a coarse region $r \in \{\text{beginning, mid-left, mid-right, ending}\}$ according to its learned policy. Second, given the selected region, a target letter position is sampled uniformly from a region-specific window within the word. The size of this window adapts to word length: shorter words have smaller windows, whereas longer words allow broader sampling ranges. This reflects the fact that spatial uncertainty and saccade amplitude increase with word length. Third, to model oculomotor noise, the final landed fixation location is sampled from a local neighborhood around the intended target position. Specifically, the executed fixation is drawn from a discrete window centered on the target, with the window size increasing with word length. This stochastic mapping captures the variability of human saccades, where intended targets are not executed precisely.

Letter sampling Once a fixation location is determined, the agent acquires visual information from a limited foveal window centered on the fixation. Only letters within this window are observed, while letters outside the window remain unobserved. The sampled letters are integrated with previously observed letters to form a partial representation of the word. Formally, the observation at fixation t consists of the subset of letters:

$$I_t = \{\ell_i \mid i \in \mathcal{F}(x_t)\},$$

where x_t is the fixation location and $\mathcal{F}(x_t)$ denotes the foveal window centered at x_t. Across successive fixations, these observations are accumulated to update the belief distribution over candidate words.

Belief update and uncertainty reduction Given the sampled letters, the agent updates its belief over candidate words using Bayes' rule:

$$b_{t+1}(w) \propto P(I_t \mid w)\, b_t(w).$$

Only the top-k most probable candidates are retained to reflect limitations in working memory. The informativeness of a fixation depends on how much the sampled letters reduce uncertainty over candidate words. Fixations that reveal highly diagnostic letter sequences lead to larger reductions in entropy, whereas less informative observations result in smaller updates. The policy therefore learns to prefer fixation locations that are expected to maximize uncertainty reduction under cost.

Likelihood function The likelihood $P(I \mid w)$ is computed with an ideal observer model that captures three intuitions: (i) fixating letters that uniquely identify a word sharply boosts its likelihood; (ii) words sharing many letters with the target remain plausible early on; (iii) as more of the target word has been viewed, non-targets are discounted more aggressively. It is an approximation function that simulates word identity uncertainty decrease when more letters are sampled. To update the belief distribution b', we retain only the top 5 most highly activated words in lexical memory, in line with the limited short-term memory capacity (C_{STM})[24].

From fixations to first-pass gaze duration The simulator outputs only the sequence of fixation targets; human data, however, are reported in time. We therefore convert each fixation into a duration using the information-gain rule: every extra bit of lexical information acquired during that fixation slows the eyes by a nearly constant amount ($\approx 2-4$ ms/bit) across languages and corpora[25,26]. The resulting gaze measure remains strictly Rayner-style: the sum of first-pass fixations, excluding any intervening saccade time[15].

Lexical processing stage. We model each fixation's duration as proportional to the amount of information processed (entropy loss) in lexical memory. After a fixation, the change in lexical entropy is $\Delta H = H' - H$ with $H = -\sum_w b(w) \log b(w)$, where $b(w)$ refers to the belief distribution over candidate words. The mean duration lexical processing in the fixation is

$$\mu_{lex} \;=\; \text{clip}\Big(d_0 + \kappa\, \Delta H,\; d_{\min},\; d_{\max}\Big) \tag{10}$$

where $d_0 = 200$ ms is the baseline fixation duration (the modal single-fixation time reported for silent English reading[15], with $d_{\min} = 200\ ms, d_{\max} = 250\ ms$). The slope parameter is optimized as $\kappa = 3.50$ ms·bit^{-1} to fit human data. It quantifies the processing cost of lexical uncertainty: each additional bit of entropy reduction adds approximately 3.75 ms to fixation time, within the $2 - 4$ ms bit^{-1} cross-linguisitic range reported by Wilcox et al.[25]. To reproduce realistic fixation-time variability, the observed duration at each time step t is drawn as $d^t_{lex} \sim \Gamma(k{=}2,\ \theta = \mu_{lex}/k)$, introducing the right-skew and long tails characteristic of empirical fixation distributions[27].

First-pass gaze duration. For a word, gaze duration (GD) is defined as the sum of all first-pass fixation durations[15]:

$$\text{GD} = \sum_{t \in \text{first-pass}} d^t_{lex} \tag{11}$$

Saccade duration Although the duration of saccades typically ranges from 20 to 50 ms depending on saccade length, we follow EZ-Reader[28] in simplifying this by setting the saccade duration $d_{saccade}$ to a fixed value at 25 ms. Importantly, this fixed duration is not included in the gaze duration during word recognition, but is added later when computing total reading time for evaluating reading performance under time pressure.

Non-fixation overheads Beyond fixation-based gaze duration (GD), we also account for total time spent on each word. This is necessary for time-sensitive evaluations such as reading under time pressure. Total elapsed time includes fixation durations, saccade durations, and additional intervals when the eyes are not fixating but time is still consumed (e.g., blinks or tracking losses). Classic reading work indicates that eye fixation times contribute about 90% of total reading time, implying $\approx$ 10% non-fixation overhead even with ideal tracking[29]. We therefore include a single overhead fraction ρ that represents all non-fixation and non-saccade intervals (blinks, and tracking losses). If a fixation's lexical time is d_{lex}, its time-accounted value is

$$d_{inflated\ lex} = d_{lex} + \frac{\rho}{1-\rho} d_{lex} \tag{12}$$

This construction guarantees that a proportion ρ of total time is allocated to non-fixation and non-saccade processes. We fit ρ to our human data. Accordingly, the total elapsed time for recognizing the word is

$$D_{\text{word}} = \sum_{t \in \text{first-pass}} (d_{\text{inflated lex}} + d_{\text{saccade}}) . \tag{13}$$

1.4.2 Sentence reader

At the medium time scale, the agent solves a *sentence reading* control problem. Given a sentence presented on the text display, the objective is to comprehend it as accurately and efficiently as possible. This requires deciding which words to fixate in order to update the agent's belief state about sentence meaning stored in the short-term memory. The agent faces a trade-off: reading too quickly (skipping many words) risks information loss or misinterpretation during integration, whereas reading too slowly (excessive regressions) delays progress. Physiologically, sentence reading is further complicated by *parafoveal preview*: when fixating on a word, the agent also receives partial information about adjacent words, represented as probabilistic predictions in the belief state. This preview, when aligned with contextual expectations from memory, influences whether the agent skips, fixates, or regresses. The goal is to deploy eye movements that maintain reading fluency without sacrificing comprehension. We formalize this as a POMDP, in which the agent integrates noisy visual input and evolving contextual memory to choose intra–sentence eye movements (skips, fixations, and regressions) that maximize comprehension while minimizing fixation cost.

Transition T. Eye movements are deterministic: the fixation lands on the word requested by m. The belief over the next word is stochastically updated using a pretrained language model: conditioned on (i) the parafoveally previewed letters (the first two letters of the next word $n_{parafov}$[15]) and (ii) the context $\mathbf{c}$:

$$b'_{\text{next}}(w) \propto P(w \mid \text{letters})\, P_{\text{LM}}(w \mid \mathbf{c}). \tag{14}$$

To implement, we query the pretrained language model Bert[30] twice. To obtain $P(w \mid \text{letters})$, we mask the unread portion of the next word and ask the model to predict completions consistent with the visible prefix (e.g., first three letters); the resulting softmax over the model's output tokens yields a probability

distribution over all candidate words. (2) To obtain $P_{\text{LM}}(w \mid \mathbf{c})$, we provide the preceding context tokens from the short-term memory buffer and extract the model's conditional next-word probabilities from its output logits. The two distributions are then multiplied element-wise and renormalized to form the posterior belief $b'_{\text{next}}(w)$, which guides the agent's subsequent decision to skip, fixate, or regress. Implementation details are provided in codes.

Reward r. Each step incurs an oculomotor cost of -0.1; for a REGRESS move this cost is multiplied by w_{reg}:

$$r_t = \begin{cases} -0.1 & m \in \{\textsc{next}, \textsc{skip}\} \\ -0.1\, w_{\text{reg}} & m = \textsc{regress} \\ 100\, U(\psi_{1:N}) & \beta = \textsc{stop} \text{ and the sentence is finished} \\ -100 & \beta = \textsc{stop} \text{ but the sentence is unfinished}\,, \end{cases} \tag{15}$$

where $U(\psi_{1:N}) = \left(\prod_{i=1}^{N} \psi_i\right)^{1/N}$ is the geometric mean of word-level appraisal scores. During training w_{reg} is resampled uniformly in $[0, 1]$ to encourage the agent to explore a spectrum of regression costs as a part of the reward shaping; at test time we treat w_{reg} as a single tunable parameter aligned to human data.

Computation of integration difficulty. Integration difficulty for each word was quantified using surprisal, defined as the negative log probability of the word given its preceding context. Formally, for a target word w_i with preceding context $w_{1:i-1}$, surprisal is computed as $-\log P(w_i \mid w_{1:i-1})$. We approximate contextual probability using a masked language model (BERT)[30], following prior work linking probabilistic expectations to processing difficulty[26,31].

Specifically, for each word, we construct a masked sentence by replacing the target word with a `[MASK]` token and compute the model's predicted probability for the true word at that position. The resulting probability $P(w_i \mid \text{context})$ is extracted from the softmax distribution over the vocabulary at the masked position. Surprisal is then computed as the negative logarithm of this probability. To obtain a bounded measure for use in the control policy, surprisal is further transformed into a sigmoid function, yielding a normalized integration difficulty score in the range $[0, 1]$. In addition to raw probability and surprisal, we also compute a rank-based integration score by locating the target word within the model's top-k

predictions and mapping its rank to a coarse probability scale. This procedure provides a unified measure that captures both lexical properties (e.g., frequency) and contextual predictability, allowing integration difficulty to reflect combined sources of processing demand.

Computation of sentence-level ambiguity We calculated sentence-level ambiguity as a continuous measure of contextual uncertainty during incremental sentence processing[32]. Specifically, for each sentence, we quantified the uncertainty of predicting upcoming linguistic input using a pretrained autoregressive language model (GPT-2)[33]. At each incremental step, we computed the predictive entropy of this distribution, defined as $H = -\sum_i p_i \log p_i$, where p_i denotes the probability assigned to token i. This entropy captures the model's uncertainty about the upcoming continuation given the current context: higher entropy indicates greater uncertainty and thus greater potential ambiguity in how the sentence may unfold. To obtain a sentence-level measure, we averaged the predictive entropy values across all positions within the sentence, excluding the initial position where no prior context is available. This results in a single scalar value per sentence reflecting the overall degree of contextual uncertainty encountered during incremental processing.

1.4.3 Text reader

At the longest time scale, the agent solves a *text reading* control problem. Given a multi–sentence passage, the objective is to build an accurate and coherent mental representation of the text while reading as efficiently as possible. This requires deciding which sentence to read next in order to update the agent's belief state about text meaning, maintained in short–term memory and later consolidated into long–term memory as gist. The agent faces a strategic trade-off: revisiting sentences can strengthen comprehension and coherence, but slows overall progress; avoiding revisits speeds progress, but risks gaps or inconsistencies in the mental model. Because comprehension tasks instruct participants to understand the entire passage, readers seldom skip full sentences, focusing instead on optimizing rereading and integration. Consistent with this, our model does not include sentence-level skipping.

At each step, the agent chooses the next sentence to read or revisit, aiming to maximize overall comprehension while incurring a cost for each fixation.

Transition T. The text itself is static; eye movements land deterministically on the requested sentence. Appraisal values φ_j are updated according to the Construction–Integration framework[34]. Specifically, a pretrained language model estimates how well the current sentence integrates with the previously read context by computing its conditional probability (or coherence score) given the prior sentences. This value is normalized to $[0, 1]$, and stored as the sentence's appraisal φ_j, representing the ease of semantic integration within the evolving discourse.

Reward r.

$$r_t = \begin{cases} -0.1 & \text{each fixation step} \\ 100\, C_{\text{softmin}}(\varphi_{1:M}) & \beta = \textsc{stop} \text{ and the entire text (} M \text{ sentences) has been read} \\ -100 & \beta = \textsc{stop} \text{ but the text is } \textit{not} \text{ finished,} \end{cases} \tag{16}$$

where the terminal comprehension score is

$$C_{\text{softmin}}(\varphi_{1:M}) = \frac{\sum_{j=1}^{M} e^{-\varphi_j/\tau}\, \varphi_j}{\sum_{j=1}^{M} e^{-\varphi_j/\tau}}, \tag{17}$$

with temperature hyper-parameter $\tau = 0.1$. The soft-minimum emphasises any poorly understood sentences: if one φ_j is low, it pulls the weighted mean down disproportionately, encouraging regressions to weak points.

Memory System At the text level our agent mirrors the core assumptions of Kintsch's Construction–Integration (C–I) theory[34]. Each processing cycle proceeds as follows.

1. Parsing in working memory. The sentence currently being read is parsed into a set of micro-propositions (or concepts) $\{\pi_1, \pi_2, ...\}$ using a pretrained language model.

2. Schema-guided selection. A schema – an abstract knowledge skeleton encoding topic-relevant background information – scores each proposition for relevance and coherence. Propositions whose

activation exceeds a threshold are pushed as gist candidates.

3. Integration into long-term memory. Gist candidates are transferred to long-term memory, where they update the memory and schema. Reading is a cyclically processing concepts, re-encountered propositions receive additional activation, gradually strengthening their memory traces and increasing their later retrievability. We introduce activation thresholds $\tau_{\text{H or L}}$, which determines whether a micro-structural proposition is integrated into the LTM gist based on its relevance, importance, and coherence. This threshold is a tunable parameter, capturing individual differences in prior knowledge and memory selectivity.

1.4.4 Reading under time pressure

To model the speed–accuracy trade-off imposed by time pressures we keep the core POMDP structure described above and introduce three targeted changes – (i) state augmentation, (ii) episode termination, and (iii) reward shaping – at the sentence and text scales.

1. **Word level.** No additional time variable is introduced; fixation-scale behavior is assumed to be governed solely by oculomotor cost, exactly as in the baseline model.

2. **Sentence level.** At this level the agent faces a hard *step budget* N_b that is derived from the global reading deadline (30 s, 60 s, 90 s) and the sentence length N. We first convert the objective deadline into a *time-pressure scalar* x, reflecting how time pressure is nonlinearly propagated to sentence-level information processing, consistent with prior findings on temporal constraints in cognitive tasks[35]:

$$x = \frac{T_{\text{obj}}}{T_{\text{baseline}}}, \qquad T_{\text{baseline}} = N/r_{\text{base}}, \tag{18}$$

and then map it through a one-parameter hyperbola

$$f(x; w_{TP}) = 1 - \frac{w_{TP}}{x+1} \quad \longrightarrow \quad N_b = \lceil f(x; w_{TP})\, N \rceil \tag{19}$$

a parameter that modulates the nonlinearity with which time pressure shapes the step budget. In our implementation, we empirically set w_{TP} to a fixed value of 0.35.

State Compared with the no-pressure model, the Markov state now carries two additional signals $\Delta n_t = N_b - t$ (normalized remaining steps) and a binary flag $\text{overtime}_t = \mathbb{I}[\,t > N_b\,]$

Transition Every eye-movement action decrements Δn_t; when $t = N_b$ the environment does *not* truncate the episode, but sets $\text{overtime}_t = 1$ and continues, it mirrors the fact that human readers rarely abandon a sentence mid-way. Instead, the environment flips a binary flag $\text{overtime}_t = 1$ and keeps running, and applies a small overtime penalty for every subsequent fixation. If the agent skips a word, its integration value is multiplied by a degradation factor w_{IL} (fit on human data), reflecting the information loss that humans experience when they skip.

Reward Time pressure is handled purely through two new terms in r_t

(a) A small step penalty $r_{\text{o/t}} = -1$ whenever $\text{overtime}_t = 1$.

(b) A terminal cost -10 if the agent terminates before reading all words, otherwise the comprehension bonus only.

3. **Text level.** The top-level state now carries the *global* remaining time Δt. A text-level episode terminates automatically when $\Delta t \leq 0$ or when the agent issues the stop action ($\beta = 1$). The terminal reward keeps the soft-minimum comprehension score C_{softmin} (see previous subsection) but is modulated by a coverage bonus: $r = 100 C_{\text{softmin}}(\varphi_{1:M})(\frac{N_{read}}{N_{total}})^{w_{RP}}$, where N_{read} is the number of sentences actually read and N_{total} the total sentences in the passage. The exponent w_{RP} tunes the importance of making progress that balances quality and quantity: when time is tight the agent can gain by reading fewer sentences very well, but under generous time pressures, the same formula rewards thorough coverage. We treat it as a tunable parameter that is used to fit the simulation results to human data.

With these minimal changes the hierarchical agent learns to reproduce the human pattern observed in the time-pressure experiment.

1.5 Training

We trained the three hierarchical controllers – **word recognizer**, **sentence reader**, and **text reader** independently, each in an environment tailored to its functional scope. This modular training scheme enables the agent to operate with high fidelity when reading full texts, directly sampling from detailed letter-level visual input in the external environment.

A flat RL approach (a single policy network trained end-to-end) proved ineffective in our trials: the agent failed to acquire stable high-level reading strategies because the rewards for sentence- and text-level comprehension were both extremely sparse and temporally distant from individual eye-movement actions. The hierarchical decomposition overcomes this sparse-reward trap by assigning each level a local resource-rational objective with denser feedback, allowing the word-level controller to master efficient letter sampling, the sentence-level controller to optimize fixation, skipping, and regression, and the text-level controller to plan revisits for global comprehension.

The environments were either synthetic corpora generated by a stochastic text generator or human corpora drawn from established reading datasets. It is important to note that **no model** was trained on human behavioral data (eye movements, comprehension scores, or memory reports). We used only the same stimuli from which human datasets were collected. This design ensures compatibility for direct model–human comparisons while preserving the model's capacity for prediction of human-like behavior.

1.5.1 Word recognizer

The word-level agent was trained exclusively on synthetic corpora. Human reading datasets typically contain a limited vocabulary and a narrow distribution of lexical features; training directly on them would risk overfitting to specific lexical memories rather than learning a generalizable eye movement policy. By generating randomized words and associated lexical memory states, the agent could explore freely, learning to minimize information uncertainty through efficient fixation strategies. This synthetic setup promotes robust policies that generalize across unseen words and contexts.

1.5.2 Sentence reader

The sentence-level agent was trained on the corpus from the ZuCo dataset[4], using only its textual stimuli rather than human eye movement data. This ensured that training occurred in a realistic linguistic environment while maintaining independence from human behavioral data. To simulate parafoveal preview and build accurate belief states, the agent required three key lexical features for the upcoming word: (1) Likelihood (letter-to-word mapping), (2) Contextual predictability, and (3) Word frequency. Likelihood and predictability values were precomputed offline using a pretrained BERT model[30] for efficiency and consistency. Word frequency was computed from the SUBTLEX-US corpus[36]. The abundance of unique sentences in ZuCo reduces overfitting risk at this level.

1.5.3 Text reader

The text-level agent was trained on synthetic multi-sentence corpora, with sentence appraisals randomized during training. To maintain realism, the randomized sentences were constrained to match the text length and sentence length distributions observed in our experimental corpus. This constraint ensured that training conditions were comparable to evaluation settings, while still benefiting from the diversity of a pseudo-stochastic corpus. This setup allowed the agent to learn a generalized control policy for deciding where and when to read or revisit at the sentence level. Evaluation was performed exclusively on naturalistic human texts to ensure ecological validity.

1.5.4 Models with time-pressure awareness

For agents with time-pressure awareness, we used the same training procedures and corpora as above, with the addition of time-budget constraints in the environment's reward and termination conditions.

1.5.5 Implementation details

For each model's training, hyperparameters followed SB3 defaults unless noted. Training ran on a single Nvidia Tesla V100 GPU, 20 M simulation steps (12 h wall-clock) for the word level; 20 M (12 h) for the sentence level; and 20 M (12 h) for the text level. We first evaluated each controller in isolation, then stack them (Text, Sentence, and Word) for a holistic assessment. All test stimuli are naturalistic and identical to

those shown to human participants.

1.6 Parameters optimization

1.6.1 Parameters determined from literature

Several parameters in our model were set directly based on empirical findings. For visual perception, the foveal window size n_{fov} was set to 8 letters, based on Rayner's report that human readers perceive approximately $7 - 9$ letters with high acuity[15]. For parafoveal preview, we set $n_{parafov} = 2$, following Rayner's finding that up to the first 3 letters of the next word can be reliably processed[15]. Regarding eye movements, the saccade execution time $d_{saccade}$ typically ranges from 20 to 50 ms depending on saccade length, but its effect is minor relative to gaze durations. To keep the model simple, we follow EZ-Reader in setting this value to a constant 25 ms[28]. Lastly, the short-term memory capacity C_{STM}, which determines how many word candidates can be actively maintained, was set to 5, within Miller's classic range of $5 - 9$ items[24].

1.6.2 Parameters optimized

Other parameters were treated as tunable and optimized to fit behavioral patterns. For the processing cost per bit of lexical information gain, κ-which specifies the milliseconds of additional gaze duration incurred per bit-we optimize κ within the empirically established range 2–4 ms/bit[25,26]. Non-fixation overhead fraction: fixation epochs contribute about 90% of reading time[29], while in task-general free viewing fixation periods comprise around 80%[37], suggesting $10 - 20\%$ total non-fixation time. We therefore set $\rho \in [0.10, 0.30]$. For long-term memory integration, we introduce three thresholds τ_{LTM}, τ_{H} and τ_{L} that governs which microstructural propositions are encoded into the long-term gist for normal readers, and readers with high and low prior knowledge readers respectively. This reflects prior work showing that integration depends on a proposition's global relevance, importance, and coherence[34,38]. Since activation scores are normalized to the range $[0, 1]$, $\tau_{\text{LTM or H or L}}$ are optimized within the same interval. Time pressure is modeled using a non-linear mapping, consistent with evidence that perceived pressure does not scale linearly with available time for information processing[35]. To account for information loss due to skipping, we introduce w_{IL}, a degradation factor that down-weights the integration value of skipped

words[39], and optimize it within $[0, 1]$. Finally, we tune two parameters governing reading preferences: w_{reg} controls the penalty for regressions (optimized over $[0, 6]$) and w_{RP} controls the agent's preference for forward reading progress rather than revisiting prior text (optimized over $[0, 3]$).

1.6.3 Discrepancy function

We tune the model parameters $\boldsymbol{\phi}$ by minimizing a composite discrepancy that (i) uses a variance–aware, curve–level loss when only bin means are available (Secs. 2.1–2.2), and (ii) uses a distributional divergence when full empirical distributions are available (Secs. 2.3–2.4). Let M_{curve} be the set of metrics evaluated via conditional bin means and M_{dist} the set with conditional distributions. Our objective is

$$F(\boldsymbol{\phi}) \;=\; \underbrace{\sum_{m\in M_{\text{curve}}} \alpha_m\, \chi^2_m(\boldsymbol{\phi})}_{\text{curve loss on bin means}} \;+\; \underbrace{\sum_{m\in M_{\text{dist}}} \beta_m \sum_{c\in C_m} \omega_{m,c}\; \mathrm{JS}\big(G(m\mid c)\,\|\,H(m\mid c)\big)}_{\text{distributional loss}}, \tag{20}$$

with nonnegative weights $\alpha_m, \beta_m, \omega_{m,c}$ (default $= 1$), and where $\mathrm{JS}(\cdot\|\cdot)$ denotes the Jensen–Shannon divergence.

Curve loss for bin means. For a metric $m \in M_{\text{curve}}$ conditioned on a factor x (e.g., word length L, log frequency f, or logit predictability p), let $\mu^{\text{sim}}_m(x)$ and $\mu^{\text{hum}}_m(x)$ be the simulated and human *bin means*, and let $\sigma^{\text{sim}}_m(x)$ and $\sigma^{\text{hum}}_m(x)$ denote their standard errors (when available). We compare curves over a common set of conditioning values $\mathcal{X}^*_m$ and minimize the reduced chi-square:

$$\chi^2_m(\boldsymbol{\phi}) \;=\; \frac{1}{|\mathcal{X}^*_m|} \sum_{x\in\mathcal{X}^*_m} \frac{\big(\mu^{\text{sim}}_m(x) - \mu^{\text{hum}}_m(x)\big)^2}{\sigma^{2,\text{sim}}_m(x) + \sigma^{2,\text{hum}}_m(x) + \sigma_0^2}, \tag{21}$$

where $\sigma_0 > 0$ is a small stability floor used when standard errors are missing (so that (21) reduces to an inverse-variance–weighted MSE on the ms scale).

Line–matching loss for conditional means. For a metric $m \in M_{\text{curve}}$ conditioned on a factor x (e.g., word length L, log frequency f, or logit predictability p), let $\mu^{\text{hum}}_m(x)$ and $\mu^{\text{sim}}_m(x)$ denote the human and simulated conditional mean curves. We first align the two curves on their common x–domain $\mathcal{X}^*_m$. On this

shared domain, we fit ordinary least–squares (OLS) lines to each curve,

$$\mu_m^{\text{hum}}(x) \approx a_m^{\text{hum}} + b_m^{\text{hum}} x, \qquad \mu_m^{\text{sim}}(x) \approx a_m^{\text{sim}} + b_m^{\text{sim}} x, \tag{22}$$

using the same x–range $\mathcal{X}_m^*$ for both fits (implemented via `numpy.polyfit`). The per–metric loss penalizes slope and intercept discrepancies between these two fitted lines:

$$\mathcal{L}_m(\boldsymbol{\phi}) \;=\; \lambda_s \big(b_m^{\text{sim}} - b_m^{\text{hum}}\big)^2 \;+\; \lambda_a \big(a_m^{\text{sim}} - a_m^{\text{hum}}\big)^2, \tag{23}$$

with nonnegative weights λ_s, λ_a (default $\lambda_s = \lambda_a = 1$; in some experiments we prioritize trend matching by setting $\lambda_s > \lambda_a$). To balance scales across metrics, we optionally apply an affine normalization on the overlap $\mathcal{X}_m^*$ that maps x and $\mu_m^{\text{hum}}(x)$ to $[0, 1]$ before fitting; the same transform is then applied to $\mu_m^{\text{sim}}(x)$. Our overall objective over curve–based metrics is

$$F(\boldsymbol{\phi}) \;=\; \sum_{m \in M_{\text{curve}}} \alpha_m \, \mathcal{L}_m(\boldsymbol{\phi}), \tag{24}$$

with nonnegative weights α_m (default = 1). Terms with fewer than two points per side on $\mathcal{X}_m^*$ are omitted from the sum.

Squared Sum Errors (SSE). When only a small set of conditional *means* is available (here, four cells crossing text coherence and prior knowledge), we tune parameters by minimizing a simple sum of squared errors (SSE) between simulated and human cell means. Let $C = \{\text{FH}, \text{FL}, \text{MH}, \text{ML}\}$ denote the cells (Fully coherent × High knowledge, Fully coherent × Low knowledge, Minimally coherent × High knowledge, Minimally coherent × Low knowledge). For each cell $c \in C$, let $\mu^{\text{hum}}(c)$ be the human mean and $\mu^{\text{sim}}(c; \boldsymbol{\phi})$ the simulator mean under parameters $\boldsymbol{\phi}$. Our objective is

$$\text{SSE}(\boldsymbol{\phi}) \;=\; \sum_{c \in C} w_c \left(\mu^{\text{sim}}(c; \boldsymbol{\phi}) - \mu^{\text{hum}}(c)\right)^2, \tag{25}$$

with nonnegative weights w_c (default $w_c = 1$). In our application, $\boldsymbol{\phi}$ are the LTM activation thresholds $(\tau_{\text{H}}, \tau_{\text{L}})$; we evaluate $\text{SSE}(\boldsymbol{\phi})$ on a uniform grid over $[0, 1]$ for each threshold and select the minimizer.

Distributional loss. For metrics with full conditional distributions, we minimize the Jensen–Shannon divergence across conditioning levels:

$$\sum_{m \in M_{\text{dist}}} \beta_m \sum_{c \in C_m} \omega_{m,c} \ \mathrm{JS}\big(G(m \mid c) \,\|\, H(m \mid c)\big), \tag{26}$$

where $G(m \mid c)$ and $H(m \mid c)$ are the empirical distributions (e.g., histograms or kernel density estimates) of the simulated and human data for metric m at level c.

1.6.4 Metric subsets and tuned parameters.

- **Word recognition.** Metrics $\mathcal{M}_{2.1}$: gaze duration (GD) conditioned on (i) word length L, (ii) log frequency f, and (iii) logit predictability p. Tuned parameter(s): $\boldsymbol{\phi}_{2.1} : \kappa$. Loss: χ^2_m per (21).

- **Sentence reading.** Metrics $\mathcal{M}_{2.2}$: skipping probability P_{skip} conditioned on L, f, p; regression probability P_{reg} conditioned on word difficulty d. Tuned parameter(s): $\boldsymbol{\phi}_{2.2} : w_{\text{reg}}$. Loss: χ^2_m per (21).

- **Text reading.** Metrics $\mathcal{M}_{2.3}$: free-recall proposition proportion R conditioned on text coherence χ and prior knowledge k. Tuned parameter(s): $\boldsymbol{\phi}_{2.3} : \tau_{\text{H or L}}$. Loss: JS per (26).

- **Reading under time pressure.** Metrics $\mathcal{M}_{2.4}$: reading speed S, skipping rate P_{skip}, and regression rate P_{reg}, each conditioned on time budget $T \in \{30, 60, 90\}$ s. Tuned parameter(s): ρ, w_{TP}, w_{IL}, w_{RP}. Loss: JS per (26).

References

[1] Reinhold Kliegl et al. "Length, frequency, and predictability effects of words on eye movements in reading". In: *European journal of cognitive psychology* 16.1-2 (2004), pp. 262–284.

[2] Keith Rayner. "Eye guidance in reading: Fixation locations within words". In: *Perception* 8.1 (1979), pp. 21–30.

[3] George W McConkie et al. "Eye movement control during reading: I. The location of initial eye fixations on words". In: *Vision research* 28.10 (1988), pp. 1107–1118.

[4] Nora Hollenstein et al. “ZuCo, a simultaneous EEG and eye-tracking resource for natural sentence reading”. In: *Scientific data* 5.1 (2018), pp. 1–13.

[5] Danielle S McNamara et al. “Are good texts always better? Interactions of text coherence, background knowledge, and levels of understanding in learning from text”. In: *Cognition and instruction* 14.1 (1996), pp. 1–43.

[6] Adrian Staub and Charles Clifton Jr. “Syntactic prediction in language comprehension: evidence from either... or.” In: *Journal of experimental psychology: Learning, memory, and cognition* 32.2 (2006), p. 425.

[7] Meredyth Daneman and Patricia A Carpenter. “Individual differences in working memory and reading”. In: *Journal of verbal learning and verbal behavior* 19.4 (1980), pp. 450–466.

[8] Francesca Zermiani et al. “InteRead: An Eye Tracking Dataset of Interrupted Reading”. In: *Proceedings of the 2024 Joint International Conference on Computational Linguistics, Language Resources and Evaluation (LREC-COLING 2024)*. 2024, pp. 9154–9169.

[9] Alan Baddeley. “Working memory”. In: *Science* 255.5044 (1992), pp. 556–559.

[10] Can Fenerci et al. “Studying memory narratives with natural language processing”. In: *Trends in Cognitive Sciences* (2025).

[11] Naser Al Madi et al. “Combining automation and expertise: A semi-automated approach to correcting eye-tracking data in reading tasks”. In: *Behavior Research Methods* 57.2 (2025), p. 72.

[12] Nicolas Vibert, Zorha Colas, and Frederic R Danion. “The impact of reading time constraints on text comprehension and eye movements”. In: *Reading and Writing* (2025), pp. 1–23.

[13] Falk Lieder and Thomas L Griffiths. “Resource-rational analysis: Understanding human cognition as the optimal use of limited computational resources”. In: *Behavioral and brain sciences* 43 (2020), e1.

[14] Samuel J Gershman, Eric J Horvitz, and Joshua B Tenenbaum. “Computational rationality: A converging paradigm for intelligence in brains, minds, and machines”. In: *Science* 349.6245 (2015), pp. 273–278.

[15] Keith Rayner. “Eye movements in reading and information processing: 20 years of research.” In: *Psychological bulletin* 124.3 (1998), p. 372.

[16] Richard S Sutton, Andrew G Barto, et al. *Reinforcement learning: An introduction*. Vol. 1. MIT press Cambridge, 1998.

[17] Erez Simony et al. “Dynamic reconfiguration of the default mode network during narrative comprehension”. In: *Nature Communications* 7 (2016), p. 12141.

[18] Christophe Pallier, Anne-Dominique Devauchelle, and Stanislas Dehaene. “Cortical representation of the constituent structure of sentences”. In: *Proceedings of the National Academy of Sciences* 108.6 (2011), pp. 2522–2527.

[19] Lijuan Wang et al. “Fast hierarchical processing of orthographic and semantic parafoveal information during natural reading”. In: *Nature Communications* 16.1 (2025), p. 8893.

[20] Yulia Lerner et al. “Topographic mapping of a hierarchy of temporal receptive windows using a narrated story”. In: *Journal of neuroscience* 31.8 (2011), pp. 2906–2915.

[21] Shubham Pateria et al. “Hierarchical reinforcement learning: A comprehensive survey”. In: *ACM Computing Surveys (CSUR)* 54.5 (2021), pp. 1–35.

[22] Nat Dilokthanakul et al. “Feature control as intrinsic motivation for hierarchical reinforcement learning”. In: *IEEE transactions on neural networks and learning systems* 30.11 (2019), pp. 3409–3418.

[23] Christoph Gebhardt, Antti Oulasvirta, and Otmar Hilliges. “Hierarchical reinforcement learning explains task interleaving behavior”. In: *Computational Brain & Behavior* 4.3 (2021), pp. 284–304.

[24] George A Miller. “The magical number seven, plus or minus two: Some limits on our capacity for processing information.” In: *Psychological review* 63.2 (1956), p. 81.

[25] Ethan G Wilcox et al. “Testing the predictions of surprisal theory in 11 languages”. In: *Transactions of the Association for Computational Linguistics* 11 (2023), pp. 1451–1470.

[26] Nathaniel J Smith and Roger Levy. “The effect of word predictability on reading time is logarithmic”. In: *Cognition* 128.3 (2013), pp. 302–319.

[27] Adrian Staub et al. "Distributional effects of word frequency on eye fixation durations." In: *Journal of Experimental Psychology: Human Perception and Performance* 36.5 (2010), p. 1280.

[28] Erik D Reichle, Keith Rayner, and Alexander Pollatsek. "The EZ Reader model of eye-movement control in reading: Comparisons to other models". In: *Behavioral and brain sciences* 26.4 (2003), pp. 445–476.

[29] Keith Rayner and Martin H Fischer. "Mindless reading revisited: Eye movements during reading and scanning are different". In: *Perception & psychophysics* 58.5 (1996), pp. 734–747.

[30] Jacob Devlin et al. "Bert: Pre-training of deep bidirectional transformers for language understanding". In: *Proceedings of the 2019 conference of the North American chapter of the association for computational linguistics: human language technologies, volume 1 (long and short papers)*. 2019, pp. 4171–4186.

[31] Roger Levy. "Expectation-based syntactic comprehension". In: *Cognition* 106.3 (2008), pp. 1126–1177.

[32] John Hale. "A probabilistic Earley parser as a psycholinguistic model". In: *Second meeting of the north american chapter of the association for computational linguistics*. 2001.

[33] Alec Radford et al. "Language models are unsupervised multitask learners". In: *OpenAI blog* 1.8 (2019), p. 9.

[34] Walter Kintsch and Teun A Van Dijk. "Toward a model of text comprehension and production." In: *Psychological review* 85.5 (1978), p. 363.

[35] Jeanne Lallement. "The effects of time pressure on information processing". In: *Recherche et applications en marketing (English Edition)* 25.4 (2010), pp. 45–69.

[36] Marc Brysbaert, Boris New, and Emmanuel Keuleers. "Adding part-of-speech information to the SUBTLEX-US word frequencies". In: *Behavior research methods* 44.4 (2012), pp. 991–997.

[37] Jorge Otero-Millan et al. "Saccades and microsaccades during visual fixation, exploration, and search: foundations for a common saccadic generator". In: *Journal of vision* 8.14 (2008), pp. 21–21.

[38] Edward J O'Brien and Anne E Cook. "Coherence threshold and the continuity of processing: The RI-Val model of comprehension". In: *Discourse Processes* 53.5-6 (2016), pp. 326–338.

[39] Sonja A Swanson et al. “What are we missing? The costs versus benefits of skip rule designs”. In: *International journal of methods in psychiatric research* 23.4 (2014), pp. 474–485.
