# Supplementary material for: Hierarchical resource rationality explains human reading behaviour
Source: Nat Hum Behav. 2026 Aug 10;10(10):2029–45. doi: 10.1038/s41562-026-02534-0 (PMC13683046; doi:10.1038/s41562-026-02534-0)
Supplement: Supplementary file 2 [file 41562_2026_2534_MOESM2_ESM.pdf]

nature portfolio

Corresponding author(s): Antti Oulasvirta

Last updated by author(s): Jun 19, 2026

Reporting Summary

Nature Portfolio wishes to improve the reproducibility of the work that we publish. This form provides structure for consistency and transparency in reporting. For further information on Nature Portfolio policies, see our Editorial Policies and the Editorial Policy Checklist.

Statistics

For all statistical analyses, confirm that the following items are present in the figure legend, table legend, main text, or Methods section.

n/a	Confirmed	
☐	☒	The exact sample size (n) for each experimental group/condition, given as a discrete number and unit of measurement
☐	☒	A statement on whether measurements were taken from distinct samples or whether the same sample was measured repeatedly
☐	☒	The statistical test(s) used AND whether they are one- or two-sided<br>*Only common tests should be described solely by name; describe more complex techniques in the Methods section.*
☐	☒	A description of all covariates tested
☒	☐	A description of any assumptions or corrections, such as tests of normality and adjustment for multiple comparisons
☐	☒	A full description of the statistical parameters including central tendency (e.g. means) or other basic estimates (e.g. regression coefficient) AND variation (e.g. standard deviation) or associated estimates of uncertainty (e.g. confidence intervals)
☐	☒	For null hypothesis testing, the test statistic (e.g. F, t, r) with confidence intervals, effect sizes, degrees of freedom and P value noted<br>*Give P values as exact values whenever suitable.*
☒	☐	For Bayesian analysis, information on the choice of priors and Markov chain Monte Carlo settings
☐	☒	For hierarchical and complex designs, identification of the appropriate level for tests and full reporting of outcomes
☐	☒	Estimates of effect sizes (e.g. Cohen's d, Pearson's r), indicating how they were calculated

Our web collection on statistics for biologists contains articles on many of the points above.

Software and code

Policy information about availability of computer code

Data collection	Eye-movement data for the new dataset ("Reading under time pressure") were collected using Tobii Pro Lab (v1.217.49450) with a Tobii Pro Spectrum (1200 Hz) eye tracker, as described in Supplementary Information Section 1.1.7. Fixation and saccade events were identified using the Tobii I-VT filter. No custom software was used for raw data acquisition beyond vendor-provided tools.
Data analysis	Data preprocessing and analysis for the "Reading under time pressure" dataset were conducted using Python (≥3.10) with standard scientific libraries (NumPy, SciPy, pandas), as described in Methods and Supplementary Information Section 1.2 and 1.4. Eye-movement correction was performed using Fix8. Model simulations and analyses were implemented using Python (repository: hierarchical-resource-rational-reader, link: https://github.com/BaiYunpeng1949/hierarchical-resource-rational-reader/). Reinforcement learning components used Stable-Baselines3. Text processing and language-model components relied on pretrained transformer models, including BERT and GPT-2, as described in Supplementary Information Section 1.4.

For manuscripts utilizing custom algorithms or software that are central to the research but not yet described in published literature, software must be made available to editors and reviewers. We strongly encourage code deposition in a community repository (e.g. GitHub). See the Nature Portfolio guidelines for submitting code & software for further information.

Data

Policy information about availability of data

All manuscripts must include a data availability statement. This statement should provide the following information, where applicable:

- Accession codes, unique identifiers, or web links for publicly available datasets
- A description of any restrictions on data availability
- For clinical datasets or third party data, please ensure that the statement adheres to our policy

All data supporting the findings of this study are available at the Open Science Framework under accession https://osf.io/q2dm6/ (DOI: 10.17605/OSF.IO/Q2DM6). These include the newly collected eye-movement and comprehension data from the reading-under-time-pressure study, simulation data generated by the model, and processed datasets used for statistical analyses and figure plotting. Publicly available datasets analysed in this study, including ZuCo, were obtained from their original sources under the terms specified by the respective dataset providers; the processed data used in our analyses are included in the OSF repository where permitted. Summary data extracted from previously published studies were used only in processed or aggregated form and are included where needed to support the analyses and figures.

Research involving human participants, their data, or biological material

Policy information about studies with human participants or human data. See also policy information about sex, gender (identity/presentation), and sexual orientation and race, ethnicity and racism.

Reporting on sex and gender	Sex was recorded by participant self-report where available and is reported descriptively. Sex or gender was not treated as an experimental variable and was not included in the study design or analysis because no sex- or gender-based hypotheses were formulated.
Reporting on race, ethnicity, or other socially relevant groupings	No analyses were conducted using race, ethnicity, or other socially relevant groupings. These variables were not used in the study design or analysis because they were not theoretically relevant to the research questions addressed.
Population characteristics	Participants were adult volunteers with normal or corrected-to-normal vision and no reported reading impairments. No clinical populations were involved. Additional task-relevant inclusion criteria are described in the Methods section.
Recruitment	Participants were recruited from a university participant pool and through campus-wide advertisements. Participation was voluntary and compensated. As with most laboratory-based behavioural studies, the sample represents a convenience sample, which may limit generalizability beyond similar populations.
Ethics oversight	At the time of data collection at HKUST Guangzhou, the newly collected reading-under-time-pressure study was conducted in accordance with the applicable local institutional practice for low-risk behavioural research. A separate prospective review by an institutional review board or ethics committee was not obtained for this dataset. The study involved adult participants, used a low-risk behavioural eye-tracking and reading-comprehension protocol, and did not involve deception, clinical intervention, vulnerable populations, biological or genetic material, or collection of personally identifying sensitive information. All participants provided informed consent before participation and consented to the use and sharing of anonymized data for scientific purposes.

Note that full information on the approval of the study protocol must also be provided in the manuscript.

Field-specific reporting

Please select the one below that is the best fit for your research. If you are not sure, read the appropriate sections before making your selection.

☐ Life sciences ☒ Behavioural & social sciences ☐ Ecological, evolutionary & environmental sciences

For a reference copy of the document with all sections, see nature.com/documents/nr-reporting-summary-flat.pdf

Behavioural & social sciences study design

All studies must disclose on these points even when the disclosure is negative.

Study description	This study combines a new quantitative laboratory-based behavioral experiments ("Reading under time pressure") with computational modeling, described in Methods and Supplementary Information Section 1.1.7. Human eye-movement data during reading were collected using eye tracking and analyzed quantitatively. The empirical data were used to evaluate and validate a hierarchical computational model of resource-rational reading behavior, described in Results Section "Speed-accuracy trade-off when reading under time pressure" and "Validating the necessity of hierarchical resource rationality".
Research sample	We recruited 39 adult participants from a university population. After data-quality exclusions, 32 participants were retained for comprehension analyses and 28 participants were retained for eye-movement analyses. The analysed comprehension sample included 19 female and 13 male participants, with mean age = 24.0 years, SD = 3.7, range = 18–34 years. Participants had normal or corrected-to-normal vision and no reported reading impairments. The sample was a laboratory-based convenience sample and was

not intended to be representative of the general population.

Sampling strategy	Participants were recruited through university mailing lists and online announcements. No formal sample-size calculation was performed before data collection. The target sample size was set at over 30 participants, which was considered sufficient to estimate the distributions of eye-movement and comprehension measures and to compare human behavioural patterns with model predictions. We recruited 39 adult participants; after data-quality exclusions, 32 participants were retained for comprehension analyses and 28 participants were retained for eye-movement analyses. Participants were compensated 50 RMB for taking part in the study.
Data collection	Data were collected in a controlled laboratory setting using a desktop-mounted eye tracker to record eye movements during reading. Participants were seated in front of a computer screen and read text passages presented on the screen while their eye movements were recorded. In addition to eye-movement data, participants completed task-related comprehension tests, including multiple-choice questions and free-recall tests. Only participant data and researcher-collected responses were recorded during data collection. The researcher was not blind to experimental condition because all participants completed all conditions in a within-subject design.
Timing	Data collection began on 17 August 2024 and ended on 22 October 2024. Data were collected over nine laboratory sessions during this period. There were no systematic gaps between collection periods that affected the composition of the sample.
Data exclusions	Data exclusions were applied according to pre-established data-quality criteria. Of the 39 participants initially recruited, 7 were excluded from comprehension analyses because of failure to complete the task, invalid behavioural data, or outlying comprehension/gaze measures. The remaining 32 participants were retained for comprehension analyses. For eye-movement analyses, an additional 4 participants were excluded because of excessive eye-tracking data loss or calibration/recording quality issues, leaving 28 participants with valid eye-movement data. No post hoc exclusion criteria were introduced. Full details are reported in the Methods and Supplementary Information Sections 1.1.7 and 1.2.
Non-participation	No participants withdrew after providing informed consent. Details are provided in the Methods section.
Randomization	A within-subject design was employed in which all participants completed reading tasks under three time-pressure conditions (30 s, 60 s, 90 s). Assignment of text stimuli to time-pressure conditions was counterbalanced across participants using a Latin square design to control for order and carryover effects. Details are provided in Supplementary Information Section 1.1.7.

Reporting for specific materials, systems and methods

We require information from authors about some types of materials, experimental systems and methods used in many studies. Here, indicate whether each material, system or method listed is relevant to your study. If you are not sure if a list item applies to your research, read the appropriate section before selecting a response.

Materials & experimental systems

n/a	Involved in the study	
☒	☐	Antibodies
☒	☐	Eukaryotic cell lines
☒	☐	Palaeontology and archaeology
☒	☐	Animals and other organisms
☒	☐	Clinical data
☒	☐	Dual use research of concern
☒	☐	Plants

Methods

n/a	Involved in the study	
☒	☐	ChIP-seq
☒	☐	Flow cytometry
☒	☐	MRI-based neuroimaging

Plants

Seed stocks	*Report on the source of all seed stocks or other plant material used. If applicable, state the seed stock centre and catalogue number. If plant specimens were collected from the field, describe the collection location, date and sampling procedures.*
Novel plant genotypes	*Describe the methods by which all novel plant genotypes were produced. This includes those generated by transgenic approaches, gene editing, chemical/radiation-based mutagenesis and hybridization. For transgenic lines, describe the transformation method, the number of independent lines analyzed and the generation upon which experiments were performed. For gene-edited lines, describe the editor used, the endogenous sequence targeted for editing, the targeting guide RNA sequence (if applicable) and how the editor was applied.*
Authentication	*Describe any authentication procedures for each seed stock used or novel genotype generated. Describe any experiments used to assess the effect of a mutation and, where applicable, how potential secondary effects (e.g. second site T-DNA insertions, mosiacism, off-target gene editing) were examined.*
