# Supplementary material for: Hierarchical resource rationality explains human reading behaviour
Source: Nat Hum Behav. 2026 Aug 10;10(10):2029–45. doi: 10.1038/s41562-026-02534-0 (PMC13683046; doi:10.1038/s41562-026-02534-0)
Supplement: Supplementary file 3 [file 41562_2026_2534_MOESM3_ESM.pdf]

nature portfolio

Peer Review File

Hierarchical Resource Rationality Explains Human Reading Behavior

Corresponding Author: Professor Antti Oulasvirta

This manuscript has been previously reviewed at another journal. This document only contains information relating to versions considered at Nature Human Behaviour.

Version 0:

Decision Letter:

23rd February 2026

Dear Mr Bai,

Thank you once again for your manuscript, entitled "Hierarchical Resource Rationality Explains Human Reading Behavior," and for your patience during the peer review process.

Your manuscript has now been evaluated by 2 reviewers, whose comments are included at the end of this letter. Please note that, while we initially invited a third reviewer, they have informed us that they will not return comments on this manuscript. Although the available reviewers find your work to be of interest, they also raise some important concerns. We are interested in the possibility of publishing your study in Nature Human Behaviour, but would like to consider your response to these concerns in the form of a revised manuscript before we make a decision on publication.

In particular, the reviewers remark on the need to (1) better discuss the current model in the context of existing ones (especially the Über-Reader model proposed by Reichle) and (2) make the paper more accessible to non-experts. We ask that you work to address both concerns in full.

Finally, your revised manuscript must comply fully with our editorial policies and formatting requirements. Failure to do so will result in your manuscript being returned to you, which will delay its consideration. To assist you in this process, I have attached a checklist that lists all of our requirements. If you have any questions about any of our policies or formatting, please don't hesitate to contact me.

In sum, we invite you to revise your manuscript taking into account all reviewer and editor comments. We are committed to providing a fair and constructive peer-review process. Do not hesitate to contact us if there are specific requests from the reviewers that you believe are technically impossible or unlikely to yield a meaningful outcome.

We hope to receive your revised manuscript within two months. I would be grateful if you could contact us as soon as possible if you foresee difficulties with meeting this target resubmission date.

With your revision, please:

• Include a “Response to the editors and reviewers” document detailing, point-by-point, how you addressed each editor and referee comment. If no action was taken to address a point, you must provide a compelling argument. When formatting this document, please respond to each reviewer comment individually, including the full text of the reviewer comment verbatim followed by your response to the individual point. This response will be used by the editors to evaluate your revision and sent back to the reviewers along with the revised manuscript.

• Highlight all changes made to your manuscript or provide us with a version that tracks changes.

• EXTENDED DATA FIGURES
When re-submitting your manuscript, please ensure that any supplementary figures and tables that are crucial to the manuscript’s conclusions are converted into Extended Data figures and tables to increase visibility of these data. Extended Data figures and tables are online-only (present in the online PDF and full-text HTML versions of the paper), peer-reviewed display items that provide essential background to the article but are not included in the main article due to space constraints. A maximum of ten Extended Data display items (figures and tables) is permitted.

Please use the link below to submit your revised manuscript and related files:

Link Redacted

Note: This URL links to your confidential home page and associated information about manuscripts you may have submitted, or that you are reviewing for us. If you wish to forward this email to co-authors, please delete the link to your homepage.

We look forward to seeing the revised manuscript and thank you for the opportunity to review your work. Please do not hesitate to contact me if you have any questions or would like to discuss these revisions further.

Sincerely,

[redacted]
Nature Human Behaviour

Reviewer expertise:

Reviewer #2: Eye movements, reading, language comprehension

Reviewer #3: Computational modelling, resource rationality, cognitive science

REVIEWER COMMENTS:

Reviewer #2 (Remarks to the Author):

This paper presents a computational model of eye-movement behaviour during reading that aims to integrate previous models of eye-movement behaviour and text comprehension processes. The paper is interesting and presents a new model that I believe will be of interest to researchers in the fields of reading and beyond. However, I have a few comments about the manuscript and the model that I think need to be addressed.

1. The introduction states that this is the first attempt to integrate models of eye-movement behaviour (e.g., EZ-Reader) and reading comprehension (e.g., construction-integration model), however this is not entirely the case. Firstly, more recent versions of EZ-Reader do include post-lexical processes (e.g. sentence-level processing) and their influence on eye-movement behaviour (e.g., Reichle et al., 2009). More recently, the Über-Reader model proposed by Reichle in 2021 integrates word-identification models of eye-movement behaviour with models of sentence and text comprehension. The model aims to explain and simulate eye movements during reading as well as comprehension outcomes, and considers word-, sentence- and text-level processes. I think a discussion of this model in relation to the one presented in the current article would be a good addition to the introduction/discussion, as their aims are similar.
2. The word identification part of the model states that the models makes a decision on "which letter to fixate" and "how to sample the letters" but it is a little bit unclear what this means exactly. I think some information about this would be useful in the methods section at least. Most importantly, I think it would be good to include model simulations for where the model decides to fixate a word as no such data is currently provided in or with the paper. How does the model decide on which letter to fixate, and how do model simulations compare to humans in this decision ? The results mention fixation locations but no data is shown about this as only gaze durations are reported for this part of the model. Do the model fixation locations resemble human data, and critically do they follow the optimal viewing position locations as other models of eye-movement behaviour and humans do ?
3. The sentence-level simulations presented here look at linguistic features that also influence word recognition, which I think should be discussed. Indeed, the current argument is that the model makes a decision about word skips based on sentence-level comprehension, however research shows that these are also influenced by word-identification processes. For example, skipping can be due to that word being identified in the parafovea, particularly short, frequent, and predictable words as shown in the simulations (Brysbaert & Vitu, 1998; Driegue et al., 2004; Blanchard et al., 1989). A discussion on why these are used as sentence-level metrics and the role of word identification processes in making these decisions should be included.
4. The sentence-level simulations also look at regressions to "lexically or contextually difficult words". Definitions for what lexically and/or contextually difficult words is meant in this particular instance is needed - is it merely frequency/length/predictability ? The authors should also specify whether between- and/or within-word regressions are included in these simulations (also in the Methods). As with the skipping rates, regressions (particularly within-word regressions) are also linked to word identification processes which should be discussed.
5. I was surprised that no other sentence-level processing was included, as other benchmark findings would provide a good test of how well the model simulates human-like reading behaviour. For example, the wrap-up effects may more directly reflect sentence-level processing than the measures currently included which can also reflect word-level processes. Other possible examples would be ambiguous or implausible sentences in which sentence-level integration influences comprehension and eye-movement behaviour.
6. For the text-level simulations, eye-movement behaviour is discussed but only simulations related to comprehension outcomes are given. Would it be possible to also include eye-movement behaviour data/simualtions as an illustration of the behaviour described in the last paragraph of this section (lines 213-221) ? It would be good for this section to be backed-up by data/simulations if possible.

7. Discussion: How does the model account for differences in task demands over and above differences in the “value of comprehension”? Does the model always assume a reading for “optimal” comprehension as default ? How does it allow for differences not only across groups/individuals but also between tasks which have been shown in the eye-tracking literature ? Does the “value of comprehension” also include factors such as motivation for example ?

References:
Reichle, E. D., Warren, T., & McConnell, K. (2009). Using EZ Reader to model the effects of higher level language processing on eye movements during reading. Psychonomic bulletin & review, 16(1), 1-21.
Reichle, E. D. (2021). Computational models of reading: A handbook. Oxford University Press.
Brysbaert, M., & Vitu, F. (1998). Word skipping: Implications for theories of eye movement control in reading. In G. Underwood (Ed.), Eye guidance in reading and scene perception (pp. 125–147). Elsevier.
Drieghe, D., Brysbaert, M., Desmet, T., & De Baecke, C. (2004). Word skipping in reading: On the interplay of linguistic and visual factors. European Journal of Cognitive Psychology, 16(1–2), 79–103.
Blanchard, H. E., Pollatsek, A., & Rayner, K. (1989). The acquisition of parafoveal word information in reading. Perception & Psychophysics, 46(1), 85–94.

Reviewer #3 (Remarks to the Author):

This paper presents an analysis of reading behavior from the perspective of resource rationality, implementing a hierarchical POMDP that allocates effort to processing the components of text at different levels in a way that is intended to be adaptive. The model produces predictions that align with human reading behavior.

I'm not an expert on the reading literature so I will focus my comments on the modeling component of the paper. In my view, the modeling made sense and was carried out well. The formulation of the problem as a POMDP was novel and interesting, but is likely to be unfamiliar to researchers coming from a more traditional psychology background. I would encourage the authors to spend more time explaining the key ideas behind the model in the paper itself at least in a way that would allow readers without technical expertise in reinforcement learning and planning to develop intuitions about what the model predicts and why. I would also suggest more clearly defining the relevant POMDPs in the paper rather than in the supplement, so that more technical readers can understand the key ideas behind the model.

One interesting choice in the model is that coordination between levels is essentially done by deference, where the policy indicates that it needs information at another level, hands off, and then waits until the process at the other level terminates and returns information. I can imagine a more sophisticated version of this where there is more sophisticated communication between levels about the kind of information that is sought or how much information is needed, which might affect the policy or stopping criterion used at the other level. It would be nice to see more comparison of how the choices made about the levels, their interaction, and their stopping rules influence model performance.

Given the kinds of models that exist in this space already, it might be good to have more detailed consideration of how the current approach relates to existing Bayesian models (e.g. the Bayesian Reader). There is some discussion of this, but I wonder whether that model might be viewed as some kind of approximation to the policies developed in the POMDP setting. Making such a connection explicit might help readers understand the key theoretical innovations in the new model.

Version 1:

Decision Letter:

Our ref: NATHUMBEHAV-26010345A

28th May 2026

Dear Dr Bai,

Thank you for submitting your revised manuscript "Hierarchical Resource Rationality Explains Human Reading Behavior" (NATHUMBEHAV-26010345A). It has now been seen by the original referees and their comments are below. As you can see, the reviewers find that the paper has improved in revision. We will therefore be happy in principle to publish it in Nature Human Behaviour, pending minor revisions to comply with our editorial and formatting guidelines.

We are now performing detailed checks on your paper and will send you a checklist detailing our editorial and formatting requirements within two weeks. Please do not upload the final materials and make any revisions until you receive this additional information from us.

Please do not hesitate to contact me if you have any questions.

Sincerely,

[redacted]
Nature Human Behaviour

Reviewer #2 (Remarks to the Author):

Thank you for sending the revisions, I think all my comments have been addressed adequately and the paper is also clearer for readers with less expertise in the type of modeling used in the article.

Reviewer #3 (Remarks to the Author):

The authors submitted a responsive revision to their manuscript, and my concerns (which were primarily about the presentation and clarity of relationship to previous work) have been addressed through their revisions.

Version 2:

Decision Letter:

Dear Professor Oulasvirta,

We are pleased to inform you that your Article "Hierarchical Resource Rationality Explains Human Reading Behavior", has now been accepted for publication in Nature Human Behaviour.

Authors may need to take specific actions to achieve compliance with funder and institutional open access mandates. If your research is supported by a funder that requires immediate open access (e.g. according to Plan S principles or the NIH public access policy) then you should select the gold OA route, and we will direct you to the compliant route where possible. Because authors warrant under our subscription licensing terms that they haven't committed to licensing any version of their article under a licence inconsistent with the terms of our agreement – including the applicable embargo period – publication under the subscription model isn't suitable for authors whose funders require no embargo.

Once your manuscript is typeset and you have completed the appropriate grant of rights, you will receive a link to your electronic proof via email with a request to make any corrections within 48 hours. If, when you receive your proof, you cannot meet this deadline, please inform us at rjsproduction@springernature.com immediately. Once your paper has been scheduled for online publication, the Nature press office will be in touch to confirm the details.

Acceptance of your manuscript is conditional on all authors' agreement with our publication policies (see http://www.nature.com/nathumbehav/info/gta). In particular your manuscript must not be published elsewhere and there must be no announcement of the work to any media outlet until the publication date (the day on which it is uploaded onto our web site).

If you have posted a preprint on any preprint server, please ensure that the preprint details are updated with a publication reference, including the DOI and a URL to the published version of the article on the journal website.

An online order form for reprints of your paper is available at https://www.nature.com/reprints/author-reprints.html. All co-authors, authors' institutions and authors' funding agencies can order reprints using the form appropriate to their geographical region.

We welcome the submission of potential cover material (including a short caption of around 40 words) related to your manuscript; suggestions should be sent to Nature Human Behaviour as electronic files (the image should be 300 dpi at 210 x 297 mm in either TIFF or JPEG format). Please note that such pictures should be selected more for their aesthetic appeal than for their scientific content, and that colour images work better than black and white or grayscale images. Please do not try to design a cover with the Nature Human Behaviour logo etc., and please do not submit composites of images related to your work. I am sure you will understand that we cannot make any promise as to whether any of your suggestions might be selected for the cover of the journal.

You can now use a single sign-on for all your accounts, view the status of all your manuscript submissions and reviews, access usage statistics for your published articles and download a record of your refereeing activity for the Nature journals.

To assist our authors in disseminating their research to the broader community, our SharedIt initiative provides you with a unique shareable link that will allow anyone (with or without a subscription) to read the published article. Recipients of the link with a subscription will also be able to download and print the PDF.

As soon as your article is published, you will receive an automated email with your shareable link.

In approximately 10 business days you will receive an email with a link to choose the appropriate publishing options for your paper and our Author Services team will be in touch regarding any additional information that may be required.

If you have any questions about our publishing options, costs, Open Access requirements, or our legal forms, please contact ASJournals@springernature.com

We look forward to publishing your paper.

With best regards,

[redacted]
Nature Human Behaviour

P.S. Click on the following link if you would like to recommend Nature Human Behaviour to your librarian http://www.nature.com/subscriptions/recommend.html#forms

** Visit the Springer Nature Editorial and Publishing website at www.springernature.com/editorial-and-publishing-jobs for more information about our career opportunities. If you have any questions please click here.**

Open Access This Peer Review File is licensed under a Creative Commons Attribution 4.0 International License, which permits use, sharing, adaptation, distribution and reproduction in any medium or format, as long as you give appropriate credit to the original author(s) and the source, provide a link to the Creative Commons license, and indicate if changes were made.
In cases where reviewers are anonymous, credit should be given to 'Anonymous Referee' and the source.
The images or other third party material in this Peer Review File are included in the article's Creative Commons license, unless indicated otherwise in a credit line to the material. If material is not included in the article's Creative Commons license and your intended use is not permitted by statutory regulation or exceeds the permitted use, you will need to obtain permission directly from the copyright holder.
To view a copy of this license, visit https://creativecommons.org/licenses/by/4.0/

Response Letter

We thank the Editor and the reviewers for their constructive and insightful feedback. We have substantially revised the manuscript to address all concerns and to strengthen both the conceptual positioning and empirical support of our work.

While all comments have been addressed, the most substantive improvements concern:

- **Stronger positioning relative to prior models**: clearer contrast with Über-Reader and Bayesian Reader (Introduction; Results: "Deciding when and where to fixate in a word," "Deciding where to fixate in a sentence"; Discussion), emphasizing policy-based control over hand-crafted control rules.
- **Clarification of hierarchical POMDP and core concepts**: refined description of belief, difficulty, cross-level interaction, and intuitions about how the model works (Computational simulation of reading; Results; Discussion; Methods).
- **Addition of new experiments and results (new analyses)**:
    - Fixation landing positions and preferred viewing position effects (McConkie et al's dataset.; Fig. 3a)
    - Within-word (intraword) regressions (Rayner's dataset; Fig. 3a)
    - Sentence-level ambiguity effects on skipping and regression behavior (ZuCo 1.0 dataset, Fig. 3b-c)
    - Text-level regression under ambiguity (Staub and Clifton's dataset; Fig. 3d)
- **Improved accessibility and clarity**: revised throughout for non-RL background readers.
- **Updated figures and Extended Data:** We include 5 Extended Data figures (numbered Fig. 6–10 following the main figure sequence).
- **Updated Supplementary Information**: Expanded methodological and implementation details.

As a result, the paper now presents a clearer and stronger contribution: a hierarchical resource-rational account in which eye-movement control and comprehension are jointly governed by policies that optimize behavior under uncertainty. Rather than relying on predefined processing stages or control heuristics, both when and where to fixate emerge from a unified control principle. The revised manuscript provides comprehensive validation across word-, sentence-, and text-level reading, including new human-model comparisons and analyses of model components and hierarchical interactions, and outlines how the approach can be extended to capture variation across tasks, individuals, and reading conditions.

Corresponding changes in the revised manuscript are highlighted in blue for ease of review. The revisions have increased the main text length to approximately 6,000 words; we would be happy to shorten the manuscript if requested by the editor.

Editor

In particular, the reviewers remark on the need to (1) better discuss the current model in the context of existing ones (especially the Über-Reader model proposed by Reichle) and (2) make the paper more accessible to non-experts. We ask that you work to address both concerns in full.

Response:

We thank the Editor and both reviewers for the clear guidance on strengthening the manuscript. We have carefully revised the paper to address all points raised. In particular:

- We have expanded the discussion of prior computational models of reading in the Introduction, Results, and Discussion in the manuscript, and also the Supplementary Information. Specifically, we provide a more explicit comparison between our hierarchical resource-rational model and the Über-Reader / EZ-Reader family of models (Reichle et al.), clarifying both shared commitments and key differences.
- We have revised the manuscript throughout to improve clarity and accessibility to non-expert audiences.
- We have conducted four more studies to answer reviewer's questions, and updated figures as well for better clarifications about the confusions.

Acknowledging Über-Reader Model (R2)

The introduction states that this is the first attempt to integrate models of eye-movement behaviour (e.g., EZ-Reader) and reading comprehension (e.g., construction-integration model), however this is not entirely the case. Firstly, more recent versions of EZ-Reader do include post-lexical processes (e.g. sentence-level processing) and their influence on eye-movement behaviour (e.g., Reichle et al., 2009). More recently, the Über-Reader model proposed by Reichle in 2021 integrates word-identification models of eye-movement behaviour with models of sentence and text comprehension. The model aims to explain and simulate eye movements during reading as well as comprehension outcomes, and considers word-, sentence- and text-level processes. I think a discussion of this model in relation to the one presented in the current article would be a good addition to the introduction/discussion, as their aims are similar.

Response:

We thank the reviewer for this important clarification. We agree that the Über-Reader (Reichle, 2021) represents a significant step toward integrating word identification, sentence processing, and discourse-level comprehension within a unified architectural framework, and we have revised the manuscript to explicitly acknowledge and discuss this work.

While both models aim to explain eye movements and comprehension across multiple levels, they differ fundamentally in how control over reading behavior is defined.

In the Über-Reader, eye-movement behavior arises from cascaded processing stages governed by parameterized control mechanisms (e.g., rules determining when to initiate saccades or trigger regressions), inherited from earlier models such as E-Z Reader, which are specified a priori. Control is implemented through these predefined mechanisms, without framing control as an explicit optimization over alternative actions.

In contrast, our model formulates reading as a sequential decision-making problem bounded by resources. Actions, i.e., where to fixate, whether to skip, and when to stop are selected by policies that optimize expected comprehension utility under cognitive and temporal constraints. Both information sampling and termination emerge from this optimization process, rather than being determined by predefined control mechanisms.

This difference is consequential: whereas the Über-Reader explains behavior through the interaction of predefined rules, our model explains the mechanisms of behavior as the emergent outcome of optimizing a unified control objective under uncertainty.

- To Introduction (page 2 and 3), we have revised our contribution as: “The first resource-rational account of reading that unifies control of eye movements and comprehension under a single optimal-control principle” and added a discussion of the Über-Reader to clarify its relationship to our work.
- To Results (page 10, 13, 15), we have added comparisons situating our model relative to existing approaches (including Über-Reader), highlighting differences in underlying principles.

- To the Discussion (page 19 and 20), we have clarified the key distinction between our model as a control formulation grounded in an explicit optimization principle, and the Über-Reader as an integrative architectural framework.

Within-word fixation mechanism and landing position validation (R2)

The word identification part of the model states that the models makes a decision on “which letter to fixate” and “how to sample the letters” but it is a little bit unclear what this means exactly. I think some information about this would be useful in the methods section at least. Most importantly, I think it would be good to include model simulations for where the model decides to fixate a word as no such data is currently provided in or with the paper. How does the model decide on which letter to fixate, and how do model simulations compare to humans in this decision ? The results mention fixation locations but no data is shown about this as only gaze durations are reported for this part of the model. Do the model fixation locations resemble human data, and critically do they follow the optimal viewing position locations as other models of eye-movement behaviour and humans do?

Response:

We thank the reviewer for this comment, and have addressed this concern in two ways:

(1) Clarifying the word-level fixation mechanism in the Methods. (page 29-31)
We now explicitly describe how the word recognizer selects fixation locations. At each step, the agent maintains a belief distribution over candidate word identities. Given this belief, it evaluates potential fixation positions in terms of their expected information gain (i.e., expected reduction in lexical entropy) relative to the associated time and oculomotor costs. The selected fixation position is the one that maximizes expected utility, defined as uncertainty reduction minus cost. This formulation clarifies what is meant by “sampling letters”: fixation positions are chosen to optimally reduce uncertainty about word identity under resource constraints. As a consequence, positions near the center of the word tend to provide higher expected information gain than peripheral positions, while also being more robust to oculomotor noise. This pattern is consistent with classic empirical findings on fixation landing positions, including the preferred viewing position effect (e.g., Rayner, 1979; Reichle et al., 2003), which we evaluate quantitatively in the Results.

(2) Adding new simulation results for fixation landing positions. (page 10)
Following the reviewer’s suggestion, we have **added new analyses** comparing simulated fixation locations to human data on first-landing position and preferred viewing position (PVP) effects. We focus on PVP, rather than the optimal viewing position (OVP), because PVP reflects the empirically observed distribution of landing positions during natural reading, whereas OVP corresponds to an idealized recognition optimum. This makes PVP the appropriate benchmark for evaluating whether the model reproduces human eye-movement behavior (Reichle et al., 2003).

The results, reported in the Results section (Fig. 3a), show that **the model successfully reproduces the characteristic human pattern** in which initial fixations are biased slightly to the left of word center, consistent with classic findings (e.g., McConkie et al., 1988). We illustrate this comparison for words of length 8 as a representative case, where the simulated distribution closely matches the human data.

Interaction between word features and sentence-level skipping decisions (R2)

The sentence-level simulations presented here look at linguistic features that also influence word recognition, which I think should be discussed. Indeed, the current argument is that the model makes a decision about word skips based on sentence-level comprehension, however research shows that these are also influenced by word-identification processes. For example, skipping can be due to that word being identified in the parafovea, particularly short, frequent, and predictable words as shown in the simulations (Brysbaert & Vitu, 1998; Driegue et al., 2004; Blanchard et al., 1989). A discussion on why these are used as sentence-level metrics and the role of word identification processes in making these decisions should be included.

Response:

We thank the reviewer for this insightful comment. We agree that skipping behavior during sentence reading is influenced not only by sentence-level comprehension processes but also by word-identification mechanisms, including parafoveal preview, lexical frequency, and contextual predictability.

In our model, these influences are explicitly incorporated within the sentence-reading controller through the agent's belief about the identity of the upcoming word. This belief represents prediction confidence and integrates three sources of information: lexical frequency, contextual expectations from previously read words, and parafoveal visual input. As a result, lexical properties such as word length, frequency, and predictability influence skipping behavior by shaping the agent's belief state.

Importantly, while lexical information affects the agent's internal beliefs, it does not directly determine eye-movement decisions. Instead, skipping emerges from optimizing a sentence-level objective: maximizing expected comprehension utility while minimizing time and oculomotor costs. When the upcoming word can be inferred with high confidence from context and preview, the agent skips it because the expected information gain from fixation is low relative to its cost.

Thus, the lexical effects reported in Fig. 3b reflect the interaction between word-identification processes and sentence-level control. We use skipping and regression as sentence-level metrics because they correspond to decisions about which word to process next, governed by the sentence-level controller and serving the integration of words into a coherent sentence representation, even though these decisions are informed by lower-level perceptual and lexical processes.

We have clarified this interaction in both the Methods (page 32 and 33) and Results (page 13) sections. In addition, we have expanded the explanation in Extended Data Fig. 7 to illustrate how prediction belief and comprehension appraisal jointly guide skipping and regression decisions.

Definition of difficulty and regression types in sentence-level simulations (R2)

The sentence-level simulations also look at regressions to “lexically or contextually difficult words”. Definitions for what lexically and/or contextually difficult words is meant in this particular instance is needed - is it merely frequency/length/predictability? The authors should also specify whether between- and/or within-word regressions are included in these simulations (also in the Methods). As with the skipping rates, regressions (particularly within-word regressions) are also linked to word identification processes which should be discussed.

Response:

We thank the reviewer for this helpful comment. We have clarified the definition of lexical and contextual difficulty, as well as the types of regressions analyzed in the manuscript.

(1) **Definition of lexical and contextual difficulty**. (page 11)
In our model, lexical and contextual difficulty are operationalized using an integration difficulty measure based on surprisal, defined as the negative log probability of a word given its preceding sentence context. This formulation is grounded in psycholinguistic theory, where surprisal has been shown to predict human reading difficulty and processing effort (e.g., Levy, 2008; Smith & Levy, 2013).

We estimate this probability using a masked language model (e.g., BERT), which predicts the likelihood of the target word given its context. Higher surprisal indicates that the word is less expected and therefore more difficult to integrate. Because surprisal reflects both intrinsic lexical properties (e.g., frequency) and contextual compatibility (e.g., predictability and semantic fit), it provides a unified and psychologically grounded operationalization of lexical and contextual difficulty. We have clarified this definition in the Methods section.

(2) **Types of regressions analyzed**. (page 12, 32)
In the sentence-reading evaluations reported in the Results section, we analyze between-word regressions. This choice is determined by the eye-tracking datasets used for evaluation, which provide reliable word-level fixation sequences but do not consistently distinguish within-word eye-movement patterns.

We have added clarification in both the Results and Methods sections specifying that the reported regression analyses refer to between-word regressions.

(3) **Relation to word-identification processes**. (page 10)
We agree that regressions, particularly within-word (intraword) regressions, are closely linked to word-identification difficulty. In our model, such behaviors arise from the interaction between word-recognition uncertainty and sentence-level control.

Although the empirical evaluations focus on between-word regressions, the model itself can generate within-word regressions during the word-recognition process. To illustrate this capability, **we have added a new simulation** showing regression behavior during word recognition (Fig. 3a). The results show that **the model successfully reproduces the**

characteristic human pattern whereby regressions are more likely to target earlier parts of a word, consistent with classic findings (e.g., Rayner, 1979).

Additional evaluation of sentence-level integration effects (R2)

I was surprised that no other sentence-level processing was included, as other benchmark findings would provide a good test of how well the model simulates human-like reading behaviour. For example, the wrap-up effects may more directly reflect sentence-level processing than the measures currently included which can also reflect word-level processes. Other possible examples would be ambiguous or implausible sentences in which sentence-level integration influences comprehension and eye-movement behaviour.

Response:

We thank the reviewer for this helpful suggestion. We agree that additional sentence-level phenomena provide important tests of whether the model captures higher-level integration processes beyond lexical influences.

Following the reviewer's recommendation, **we have added a new analysis targeting sentence-level integration** (page 12). In particular, we focused on sentence-level ambiguity, which directly affects integration difficulty and has well-established effects on eye-movement behavior.

To quantify sentence-level ambiguity, we estimated an uncertainty measure reflecting the predictability of sentence continuations in context (Hale, 2001), and examined how this predictor relates to regression and skipping probabilities across sentences. We evaluated this effect using human eye-tracking data from the ZuCo 1.0 corpus, which provides naturalistic sentence-reading behavior. As shown in the revised Results (Fig. 3b and c), **the model successfully reproduces the human pattern**: as ambiguity increases, regression probability increases and skipping probability decreases, reflecting greater integration difficulty and rereading during sentence processing.

We chose ambiguity as a primary test case because it provides a direct and reliable manipulation of sentence-level integration difficulty. We also examined wrap-up effects in the same dataset; however, the expected pattern was not clearly observed in the human data. A quantitative analysis indicated no reliable relationship between sentence position and regression probability in this dataset (Human: R^2 = 0.001). This suggests that wrap-up effects are not robustly captured in this corpus using regression-based measures. We have clarified this analysis and its interpretation in the revised Methods and Results sections.

Adding eye-movement simulations to text-level evaluation (R2)

For the text-level simulations, eye-movement behaviour is discussed but only simulations related to comprehension outcomes are given. Would it be possible to also include eye-movement behaviour data/simualtions as an illustration of the behaviour described in the last paragraph of this section (lines 213-221) ? It would be good for this section to be backed-up by data/simulations if possible.

Response:

We thank the reviewer for this helpful suggestion. Following this recommendation, we have **added a new analysis** (page 14) of text-level eye-movement behavior to complement the comprehension results.

Specifically, we now include simulations of regression behavior and compare them with established empirical findings from the eye-movement literature. For the human benchmark, we draw on results reported by Staub and Clifton (2006), who showed that readers are more likely to initiate regressions when sentences are more ambiguous (approximately 19% regressions in the ambiguous condition compared with about 6–7% in the unambiguous condition). Using the same stimuli and condition distinctions, we examined the agent's regression behavior across relatively ambiguous versus unambiguous texts. As shown in the revised Results (Fig. 3d), **the model successfully reproduces** this empirical pattern: higher ambiguity leads to increased regression probability, reflecting greater rereading during integration.

These results provide direct simulation evidence supporting the text-level eye-movement behavior described in the manuscript. Additional details are provided in the Methods and Supplementary Information.

Modeling task demands, individual differences, and motivation via reward structure (R2)

Discussion: How does the model account for differences in task demands over and above differences in the "value of comprehension"? Does the model always assume a reading for "optimal" comprehension as default ? How does it allow for differences not only across groups/individuals but also between tasks which have been shown in the eye-tracking literature ? Does the "value of comprehension" also include factors such as motivation for example?

Response:

We thank the reviewer for raising this important point. In our model, differences in task demands, individual behavior, and motivation are captured through the specification of the reward function and its associated parameters.

First, the model does not assume a fixed objective of "optimal" comprehension. Instead, behavior is governed by the reward function, which defines the agent's goal in a given task. For comprehension-driven reading (the primary focus of this paper), the reward is based on overall comprehension utility, leading the agent to balance comprehension against time and eye-movement costs. However, this objective can be modified to reflect different task demands. Specifically, different reading tasks are modeled by redefining the reward function. For example, in proofreading, rewards are tied to detecting errors, whereas in visual search tasks (e.g., locating a specific word), rewards depend on successfully identifying the target. Importantly, this allows different tasks to be modeled without introducing task-specific or hand-crafted control rules: behavior adapts through changes in the objective, while the underlying control mechanism remains the same. In this way, the same decision-making framework generalizes across tasks without requiring changes to the underlying architecture.

In addition, variability across individuals and contexts is captured by weighting parameters within the reward function. These parameters determine the relative importance of comprehension versus efficiency, and can therefore reflect differences in reader goals, time pressure, or motivation. For instance, higher weighting on comprehension leads to more careful, information-seeking behavior, whereas lower weighting produces faster, more effort-efficient reading that tolerates partial understanding.

We have clarified these mechanisms in the revised Discussion section. (page 21 and 22)

Improving accessibility and clarity of the POMDP formulation (R3)

This paper presents an analysis of reading behavior from the perspective of resource rationality, implementing a hierarchical POMDP that allocates effort to processing the components of text at different levels in a way that is intended to be adaptive. The model produces predictions that align with human reading behavior.

I'm not an expert on the reading literature so I will focus my comments on the modeling component of the paper. In my view, the modeling made sense and was carried out well. The formulation of the problem as a POMDP was novel and interesting, but is likely to be unfamiliar to researchers coming from a more traditional psychology background. I would encourage the authors to spend more time explaining the key ideas behind the model in the paper itself at least in a way that would allow readers without technical expertise in reinforcement learning and planning to develop intuitions about what the model predicts and why. I would also suggest more clearly defining the relevant POMDPs in the paper rather than in the supplement, so that more technical readers can understand the key ideas behind the model.

Response:

We thank the reviewer for this constructive suggestion. We agree that improving the accessibility of the model for readers without a reinforcement learning background is important.

In the revised manuscript, we have added an intuitive description of the POMDP formulation in the main text (Computational Simulation of Reading, page 5-8), including a concrete example illustrating how skipping and regressions emerge from belief-based decision-making under uncertainty. In addition, we have incorporated brief, high-level explanations of the modeling framework in the Methods section (page 29-35) to further support accessibility for non-technical readers.

We have also moved and clarified the core POMDP definition in the main Methods section (page 30-34), explicitly specifying the state, observation, action, reward, and belief components at each level of the hierarchy, rather than presenting these details only in the Supplementary Information.

Hierarchical coordination and policy-driven control across levels (R3)

One interesting choice in the model is that coordination between levels is essentially done by deference, where the policy indicates that it needs information at another level, hands off, and then waits until the process at the other level terminates and returns information. I can imagine a more sophisticated version of this where there is more sophisticated communication between levels about the kind of information that is sought or how much information is needed, which might affect the policy or stopping criterion used at the other level. It would be nice to see more comparison of how the choices made about the levels, their interaction, and their stopping rules influence model performance.

Response:

We thank the reviewer for this insightful comment. We agree that richer forms of communication between hierarchical levels, such as explicitly specifying the type or amount of information required, are an interesting direction for extending the model.

In our current formulation, coordination between levels is intentionally implemented through a policy-driven mechanism rather than explicit messaging or predefined stopping rules. At each level, the agent selects actions to maximize expected utility while minimizing associated costs (e.g., time and eye-movement effort). As a result, decisions about when to continue sampling information and when to terminate a sub-process emerge from the policy itself, rather than being governed by externally specified thresholds. Control is transferred across levels when the policy selects termination.

For example, during sentence reading, the agent processes the current word while forming predictions about upcoming words based on contextual information and parafoveal preview. These beliefs guide decisions such as whether to fixate, skip, or regress. When uncertainty remains high, the agent may regress to gather additional information; when sufficient confidence is reached, processing advances. At the text level, a similar mechanism governs whether further rereading is required before proceeding. In this way, coordination across levels arises from interacting policies optimized under a shared resource-rational objective.

We chose this design to maintain a unified control principle across levels, allowing both stopping behavior and inter-level coordination to emerge from the same optimization process rather than being specified through additional mechanisms. We agree that more explicit communication between levels could provide greater flexibility, and exploring such extensions would be a valuable direction for future work.

To address this, we have revised the manuscript to more explicitly describe the hierarchical structure, information flow, and policy-driven termination in the main text (Computational Simulation of Reading, page 7-8; Results: Validating the necessity of hierarchical resource rationality, page 19).

Connection to the Bayesian Reader model (R3)

Given the kinds of models that exist in this space already, it might be good to have more detailed consideration of how the current approach relates to existing Bayesian models (e.g. the Bayesian Reader). There is some discussion of this, but I wonder whether that model might be viewed as some kind of approximation to the policies developed in the POMDP setting. Making such a connection explicit might help readers understand the key theoretical innovations in the new model.

Response:

We thank the reviewer for this insightful suggestion. We agree that clarifying the relationship between our model and existing Bayesian approaches, such as the Bayesian Reader, can help highlight the theoretical contribution of our model.

The Bayesian Reader provides a principled account of word recognition as probabilistic inference under uncertainty, modeling how perceptual evidence is accumulated until a decision threshold is reached. However, this formulation assumes that visual input is given, and the primary decision concerns when to stop accumulating evidence. Accordingly, the model does not explicitly account for the control of eye movements or the selection of fixation locations.

Our model extends this framework by treating reading as an active, sequential decision-making problem bounded by resources. Rather than assuming a fixed sampling process, the agent **actively controls how information is acquired**, selecting where to fixate and what information to sample based on current uncertainty and task demands. Both information acquisition and stopping decisions are governed by policies that optimize expected comprehension utility under resource constraints.

This leads to several key advances over the Bayesian Reader. First, it enables **active control of information sampling**, rather than passive evidence accumulation. Second, it provides a **unified account of perception and action**, in which inference and eye-movement control are jointly optimized. Third, it generalizes beyond word recognition to **hierarchical reading behavior**, allowing the same control principle to operate across word-, sentence-, and text-level processes.

From this perspective, the Bayesian Reader corresponds to a setting in which information acquisition is externally determined and control is limited to stopping decisions. From this perspective, the Bayesian Reader can be interpreted as a special case or approximation of our framework in which the action space is restricted to stopping decisions and information sampling is fixed. By contrast, our model treats both sampling and termination as part of a unified decision problem, allowing information acquisition to be optimized under uncertainty and resource constraints. This shift from inference alone to integrated inference and control enables a more general account of reading behavior across levels.

We have added clarifications in the Results subsection “Deciding when and where to fixate in a word” to make this relationship more explicit (page 11).
